# NOTCH1 reverses immune suppression in small cell lung cancer through reactivation of STING

**DOI:** 10.1172/JCI185423

**Published:** 2025-07-08

**Authors:** Yoo Sun Kim, Barzin Y. Nabet, Briana N. Cortez, Nai-Yun Sun, Robin Sebastian, Christophe E. Redon, Anagh Ray, Liang Liu, Afeez A. Ishola, Sarah Loew, Anjali Dhall, Sivasish Sindiri, Velimir Gayevskiy, Min-Jung Lee, Shraddha Rastogi, Nahoko Sato, Noemi Kedei, Thorkell Andresson, Sudipto Das, Suresh Kumar, Alan E. Bers, Hongliang Zhang, Alberto Chiappori, Priyanka Gopal, Mohamed E. Abazeed, Haobin Chen, Mirit I. Aladjem, Yves Pommier, Moises J. Velez, David S. Shames, Nitin Roper

**Affiliations:** 1 Developmental Therapeutics Branch, Center for Cancer Research, National Cancer Institute, Bethesda, Maryland, USA.; 2 Genentech Inc., South San Francisco, California, USA.; 3 Surgery Branch, Center for Cancer Research, National Cancer Institute, Bethesda, Maryland, USA.; 4 Rancho Biosciences, San Diego, California, USA.; 5 Collaborative Protein Technology Resource, Center for Cancer Research, and; 6 Protein Characterization Laboratory, National Cancer Institute, Bethesda, Maryland, USA.; 7 Thoracic Oncology Program, Moffitt Cancer Center, Tampa, Florida, USA.; 8 Department of Radiation Oncology, Northwestern University Feinberg School of Medicine, Chicago, Illinois, USA.; 9 Division of Oncology, Department of Medicine, Washington University in St. Louis, St. Louis, Missouri, USA.; 10 Department of Pathology and Laboratory Medicine, University of Rochester, Rochester, New York, USA.

**Keywords:** Cell biology, Immunology, Oncology, Antigen, Biomarkers, Cancer immunotherapy

## Abstract

Downregulation of antigen presentation and lack of immune infiltration are defining features of small cell lung cancer (SCLC), limiting response to immune checkpoint blockade (ICB). While a high–MHC class I, immune-inflamed subset benefits from ICB, underlying mechanisms of immune response in SCLC have yet to be elucidated. Here we show that in the IMpower133 clinical trial, high, but not low, *NOTCH1* expression was significantly associated with longer survival with the addition of ICB to chemotherapy among approximately 80% of SCLC patients with NE-enriched tumors (*ASCL1*-enriched, HR 0.39, *P* = 0.0012; *NEUROD1*-enriched, HR 0.44, *P* = 0.024). Overexpression or pharmacologic activation of NOTCH1 in ASCL1 and NEUROD1 SCLC cell lines dramatically upregulated MHC class I through epigenetic reactivation of STING. In syngeneic mouse models, Notch1 activation reprogrammed SCLC tumors from immune-excluded to immune-inflamed, facilitating durable, complete responses with ICB combined with a STING agonist. *STING1* expression was significantly enriched in high- compared with low-*NOTCH1-*expressing tumors in IMpower133, validating our proposed mechanism. Our data reveal a previously undiscovered role for NOTCH1 as a critical driver of SCLC immunogenicity and a potential predictive biomarker for ICB in SCLC. NOTCH1 activation may be a therapeutic strategy to unleash antitumor immune responses in SCLC and other neuroendocrine cancers in which NOTCH1 is typically suppressed.

## Abstract 2

In this work, the authors uncover NOTCH1 as a critical driver of immune response and survival with immunotherapy in small cell lung cancer.

## Graphical abstract

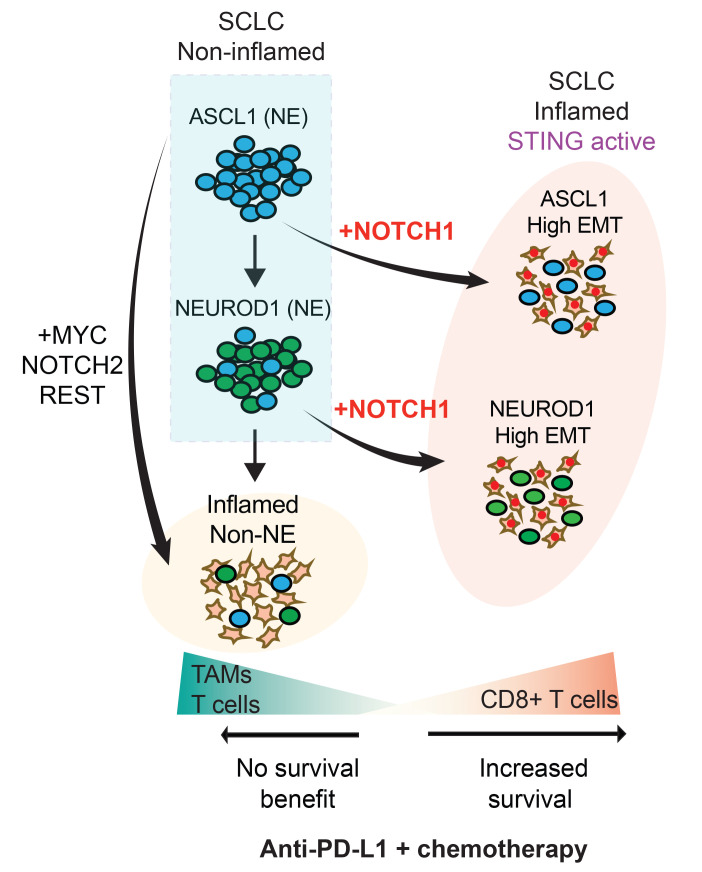

## Introduction

Tumor infiltration and recognition of cell-surface tumor antigens presented with MHC class I molecules by host T cells are critical mechanisms by which cancers are detected and ultimately destroyed (1). Small cell lung cancer (SCLC), a highly aggressive neuroendocrine (NE) neoplasm, has long been thought to evade immune response through tumor-intrinsic mechanisms, namely, silencing of antigen presentation by MHC class I downregulation (2, 3). In addition, most SCLC tumors are either devoid of tumor-infiltrating immune cells (i.e., immune desert) or contain immune cells located within the stroma or the outer margins of the tumor (i.e., immune-excluded) (4). Tumor immune infiltration (i.e., immune inflamed), while uncommon, is a determinant of long-term survival in SCLC (5). With the emergence of anti–PD-1/L1 therapy, i.e., immune checkpoint blockade (ICB) (6, 7), there has been renewed interest in developing strategies to upregulate antigen presentation and recruit immune cells into tumors, as both features have been associated with ICB response and survival in SCLC (8, 9). In addition, identification of predictive biomarkers to guide ICB treatment is considered to be a crucial step for improving SCLC clinical outcomes (10), as biomarkers such as PD-L1 expression and tumor mutation burden (TMB) are not predictive of survival with ICB in SCLC (6, 11).

Despite the lack of clinically actionable biomarkers, multiple studies have begun to elucidate the heterogeneity of the SCLC tumor microenvironment. Increased immune infiltration was observed in a group of atypical SCLC tumors characterized by low expression of NE genes, referred to as non-NE, compared with tumors with classic NE features (12). Similarly, immune response genes were found to be upregulated in non-NE SCLC but repressed in classic NE SCLC tumors and pulmonary NE cells (13). Non-NE SCLC tumors defined by the expression of YAP1 were also associated with high expression of T cell–inflamed genes (14). In regard to heterogeneity impacting ICB clinical response, patients with relapsed SCLC with non-NE tumors were found to preferentially benefit from ICB (15). Among first-line SCLC patients, there was numerically longer survival with the addition of ICB to chemotherapy in a non-NE immune-inflamed subset (SCLC-I) compared with NE subsets SCLC-A, driven by achaete-scute homologue 1 (ASCL1), and SCLC-N, driven by neurogenic differentiation factor 1 (NEUROD1); and another non-NE subset, SCLC-P, driven by POU class 2 homeobox 3 (POU2F3) (16). Last, increased immunogenicity of non-NE compared with NE SCLC has also been demonstrated using in vitro and in vivo models (17).

Recently, an unbiased transcriptomic analysis of tumors from the randomized IMpower133 clinical trial of atezolizumab in first-line SCLC reported a more nuanced relationship between ICB survival and NE status (18). This work identified an immune-inflamed, NE subset (SCLC-I-NE) that derives a statistically significant benefit from the addition of atezolizumab to chemotherapy compared with chemotherapy alone. In contrast, an immune-inflamed, non-NE subset (SCLC-I-non-NE), composed of the previously described SCLC-P subset and additional non-NE tumors, did not benefit from the addition of atezolizumab to chemotherapy, likely due to an increased presence of immune-suppressive macrophages. An additional report (19) showed no survival difference between patients with NE and non-NE tumors in the randomized CheckMate 032 clinical trial of nivolumab (ClinicalTrials.gov, NCT01928394) in relapsed SCLC. Thus, a better understanding of the mechanisms driving SCLC immune response is necessary to improve patient selection with ICB and ultimately survival in SCLC.

We previously demonstrated an association between Notch signaling and clinical benefit to ICB in relapsed SCLC (20). Notch signaling was first reported in SCLC to regulate cell growth (21) and NE differentiation through downregulation of ASCL1 (22). SCLC genetically engineered mouse models (GEMMs) subsequently demonstrated a tumor-suppressive role for Notch signaling (23). Heterogeneity of Notch signaling was also identified in SCLC GEMM models with the presence of both NE, Notch-low cells and non-NE, Notch-high cells (24). Moreover, Notch signaling was shown to be a key regulator of the NE–to–non-NE state switch that is at least partially mediated through the transcriptional repressor REST (24, 25). Additionally, through transition from the NE to the non-NE state, Notch signaling enabled the formation of vascular mimicry (26).

In this study, we sought to elucidate the potential role of Notch signaling in the SCLC immune response. Using clinical trial data, and in vitro and in vivo models, we uncovered NOTCH1, through epigenetic reactivation of STING, as a key driver of SCLC immunogenicity and survival with ICB.

## Results

### High NOTCH1 expression is significantly associated with longer overall survival with the addition of an anti–PD-L1 inhibitor to first-line chemotherapy among NE subsets of extensive-stage SCLC patients.

**Figure 1:**
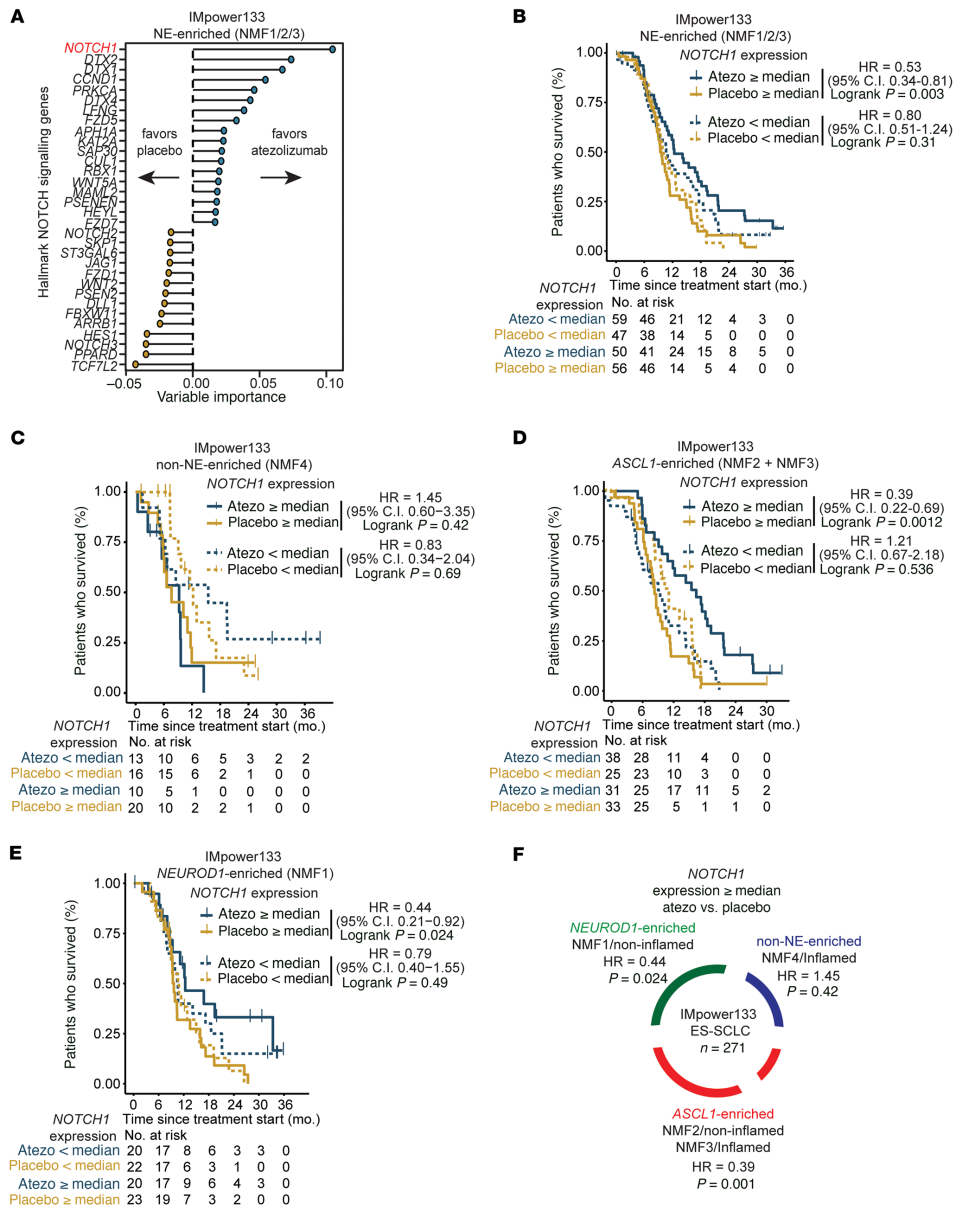
High *NOTCH1* expression is significantly associated with longer OS with the addition of atezolizumab (anti–PD-L1 inhibitor) to first-line chemotherapy among NE subsets of patients with extensive-stage SCLC in the IMpower133 clinical trial. (**A**) Unbiased generalized random forest OS analysis comparing atezolizumab with placebo using the 32 genes of the Hallmark Notch signaling gene set within the NE-enriched (NMF1/2/3) subset of the IMpower133 clinical trial. Kaplan-Meier estimates of OS among the atezolizumab and placebo treatment groups of (**B**) NE-enriched, (**C**) non-NE-enriched (NMF4), (**D**) *ASCL1*-enriched (NMF2/3), and (**E**) *NEUROD1*-enriched (NMF1) IMpower133 subsets stratified by high (greater than or equal to median) and low (less than median) *NOTCH1* expression. (**F**) Summary of OS hazard ratios, comparing atezolizumab with placebo based on high *NOTCH1* expression among the main IMpower133 subsets. Vertical lines in survival graphs represent censored patients. *P* values were calculated using a log-rank test. *P* values were unadjusted, and values less than 0.05 were considered significant. Atezo, atezolizumab; HR, hazard ratio.

Given our previous work demonstrating an association between Notch signaling and clinical benefit with ICB in relapsed SCLC (20), we hypothesized that there may be a relationship between Notch signaling and overall survival (OS) among patients with first-line ICB-treated extensive-stage SCLC. To test this hypothesis, we performed an unbiased generalized random forest analysis using the 32 genes of the Hallmark Notch signaling gene set (https://www.gsea-msigdb.org/gsea/msigdb) within the NE-enriched subset (NMF1/2/3) of IMpower133 previously shown to have longer OS with the addition of atezolizumab (anti–PD-L1 inhibitor) to chemotherapy than with placebo plus chemotherapy (18). Among the Hallmark Notch signaling genes, the model identified *NOTCH1* as the top gene that may be predictive of OS with atezolizumab over placebo (Figure 1A). Further analysis demonstrated that in this NE-enriched subset, high (defined as greater than or equal to median) *NOTCH1* expression was associated with significantly longer OS with atezolizumab compared with placebo (HR 0.53; 95% CI, 0.34–0.81; unadjusted *P* = 0.003) (Figure 1B), whereas low *NOTCH1* expression was not (HR 0.80; 95% CI, 0.51–1.24; unadjusted *P* = 0.31) (Figure 1B). In contrast, in the non-NE-enriched subset previously shown to lack an OS benefit with the addition of atezolizumab to chemotherapy (18), there were no significant differences in OS between the atezolizumab and placebo groups stratified by *NOTCH1* expression (Figure 1C). Importantly, OS among the atezolizumab and placebo treatment groups was similar irrespective of *NOTCH2* or *REST* expression in both the NE and non-NE-enriched subsets (Supplemental Figure 1, A–D; supplemental material available online with this article; https://doi.org/10.1172/JCI185423DS1). We also analyzed long-term survival (LTS; defined as ≥18-month OS) (27) and found a nonsignificant trend toward higher *NOTCH1* expression in LTS compared with non-LTS patients in the atezolizumab but not in the placebo arm (Supplemental Figure 2).

We next analyzed the relationship between *NOTCH1* expression within the individual NE subsets: *ASCL1*-enriched (NMF2/3) and *NEUROD1*-enriched (NMF1). In the *ASCL1*-enriched subset with high *NOTCH1* expression, median OS was nearly doubled with atezolizumab (16.4 months; 95% CI, 10.8–21.6) compared with placebo (8.3 months; 95% CI, 7.5–10.7) (Figure 1D). Strikingly, the HR for death was 0.39 (95% CI, 0.22–0.69; unadjusted *P* = 0.0012), and the OS rate was more than 3 times higher at 1 year with atezolizumab (61.3%) compared with placebo (17.3%) (Figure 1D). However, in the *ASCL1*-enriched subset with low *NOTCH1* expression, median OS was 2 months shorter with atezolizumab (10.6 months; 95% CI, 7.4–15.9) than with placebo (12.7 months; 95% CI, 10.0–17.3), and the 1-year OS rate was lower with atezolizumab (39.1%) than with placebo (50.6%) (Figure 1D). In the *NEUROD1*-enriched subset, high *NOTCH1* expression was also significantly associated with longer OS with atezolizumab compared with placebo (HR 0.44; 95% CI, 0.21–0.92; unadjusted *P* = 0.024), whereas low *NOTCH1* expression was not (HR 0.79; 95% CI, 0.40–1.55; unadjusted *P* = 0.49) (Figure 1E). A summary of the relationship between high *NOTCH1* expression and OS across the main subsets of IMpower133 is shown in Figure 1F.

**Figure 2:**
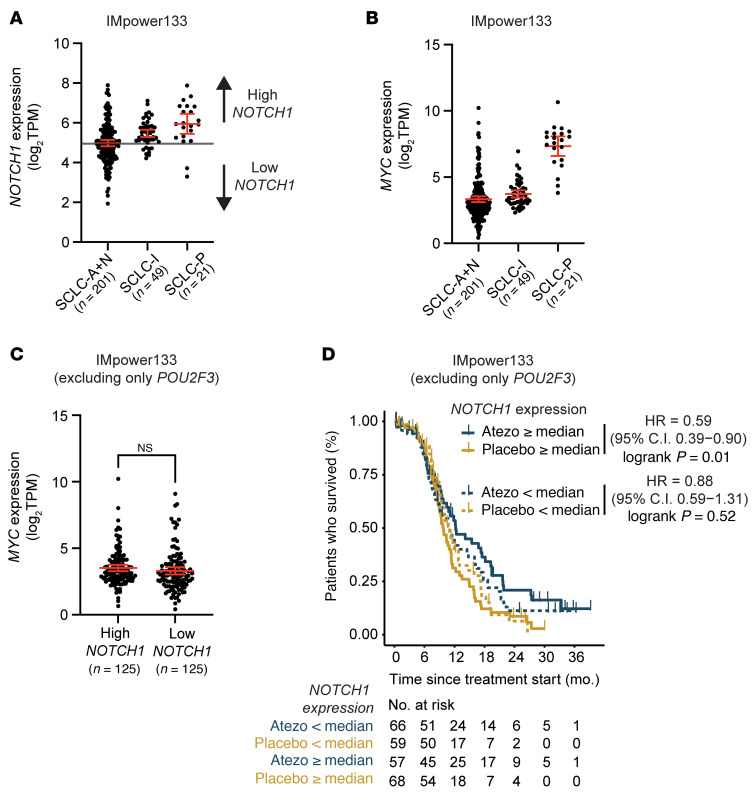
High *NOTCH1* expression is significantly associated with longer OS with the addition of atezolizumab to first-line chemotherapy among all extensive-stage SCLC patients in the IMpower133 clinical trial, except those with high-*POU2F3*-expressing tumors. (**A**) *NOTCH1* expression and (**B**) *MYC* expression among IMpower133 subsets defined by Gay et al. (16). (**C**) *MYC* expression among high- and low- *NOTCH1*-expressing tumors in IMpower133, excluding only *POU2F3*-expressing tumors. (**D**) Kaplan-Meier estimates of OS stratified by *NOTCH1* expression among the atezolizumab and placebo treatment groups of the IMpower133 trial, excluding only *POU2F3*-expressing tumors. *P* values were calculated using a log-rank test. *P* values were unadjusted, and values less than 0.05 were considered significant.

Given the differences in survival based on *NOTCH1* expression using NMF-defined subsets, we next sought to validate our results using previously defined subsets: Rudin et al. (28) (*ASCL1*, *NEUROD1*, *POU2F3*, *YAP1*) and Gay et al. (16) (SCLC-A, SCLC-N, SCLC-I, SCLC-P). Among tumors defined by high expression of *ASCL1* or *NEUROD1* (i.e., NE), high *NOTCH1* expression was significantly associated with longer OS with atezolizumab compared with placebo (HR 0.58; 95% CI, 0.38–0.87; unadjusted *P* = 0.009), whereas low *NOTCH1* expression was not (HR 0.90; 95% CI, 0.60–1.34; unadjusted *P* = 0.60) (Supplemental Figure 3A). Similarly, among SCLC-A and SCLC-N tumors, high *NOTCH1* expression was significantly associated with longer OS with atezolizumab compared with placebo (HR 0.52; 95% CI, 0.33–0.83; unadjusted *P* = 0.005), whereas low *NOTCH1* expression was not (HR 1.12; 95% CI, 0.72–1.74; unadjusted *P* = 0.62) (Supplemental Figure 3B). There were no significant differences in OS between the atezolizumab and placebo groups stratified by *NOTCH1* expression among tumors defined by high expression of *POU2F3* and *YAP1* (i.e., non-NE) or within the SCLC-P subset (Supplemental Figure 4, A and B). Within the SCLC-I subset, we observed prolonged OS with atezolizumab compared with placebo in both low- and high-*NOTCH1*-expressing tumors (Supplemental Figure 4C). Despite the stark differences in OS between SCLC-I and SCLC-P in the atezolizumab arm (Supplemental Figure 4, B and C), we observed nearly all SCLC-I (82%, *n* = 40 of 49) and SCLC-P (90%, *n* = 19 of 21) tumors to have high expression of *NOTCH1* (Figure 2A). As MYC has been shown to be a driver of Notch signaling in SCLC (29) and may impair response to ICB in lung cancer (30), we examined *MYC* expression across these subsets and found very high *MYC* expression in SCLC-P, but not in SCLC-I or SCLC-A/N (Figure 2B), and no difference in *MYC* expression between NE-enriched tumors stratified by *NOTCH1* expression (Figure 2C). Consequently, after exclusion of SCLC-P tumors, high *NOTCH1* expression was associated with significantly longer OS with atezolizumab compared with placebo (HR 0.59; 95% CI, 0.39–0.90; unadjusted *P* = 0.01) among the remaining IMpower133 dataset, whereas low *NOTCH1* expression was not (HR 0.88; 95% CI, 0.59–1.31; unadjusted *P* = 0.51) (Figure 2D). Importantly, we found no significant association between *NOTCH1* expression and OS among NE-enriched (NMF1/2/3) SCLC limited-stage tumors (23, 31) demonstrating that *NOTCH1* expression is not prognostic in SCLC (Supplemental Figure 5). Together, our data suggest that *NOTCH1* expression is predictive of OS among NE subsets of patients with SCLC treated with first-line ICB plus chemotherapy.

### Regulation and expression of NOTCH1 is distinct from those of NOTCH2 and REST in SCLC.

**Figure 3:**
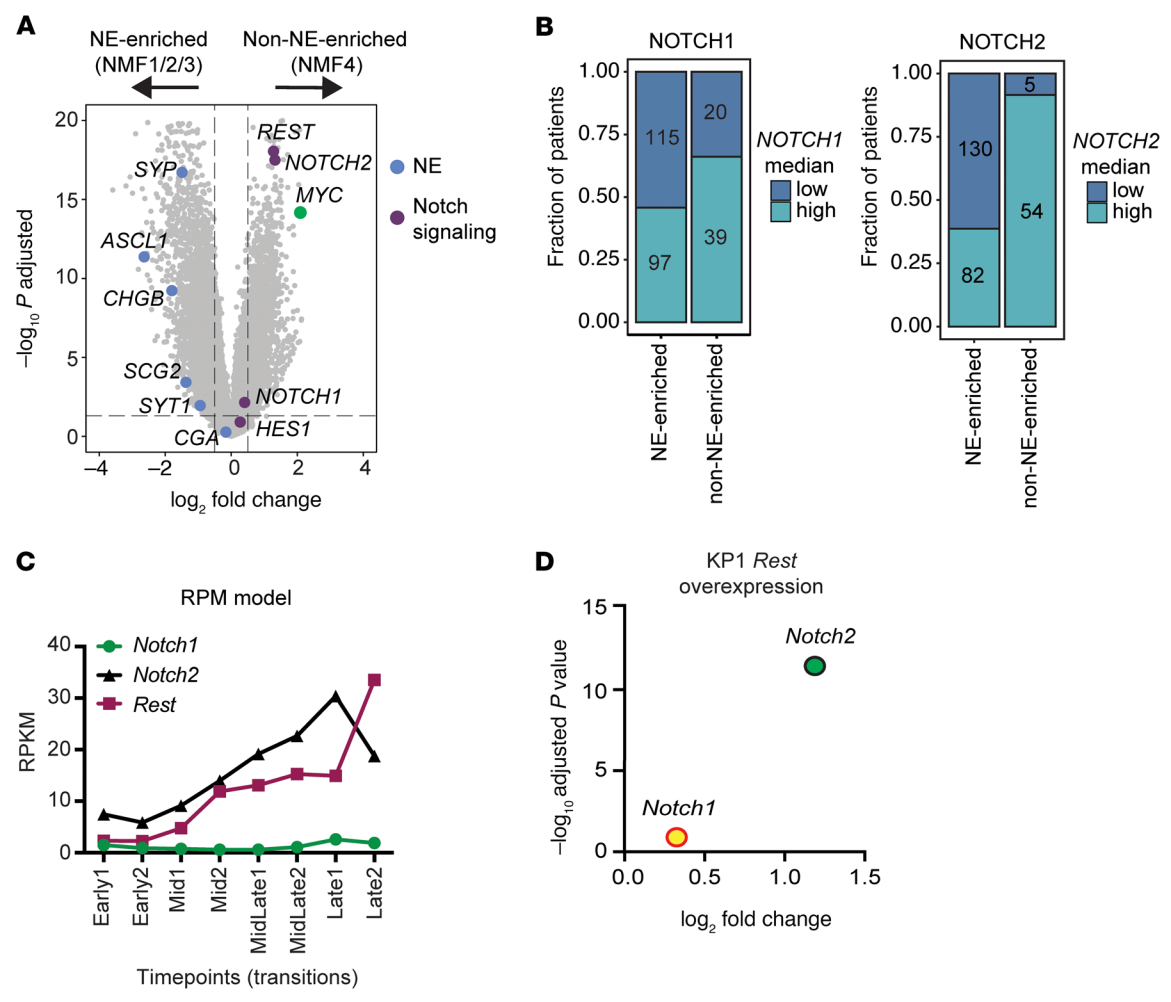
*NOTCH1* exhibits a regulatory and expression pattern distinct from those of *NOTCH2* and *REST*. (**A**) Volcano plot showing Notch signaling, NE, and MYC genes differentially expressed between NE-enriched and non-NE-enriched tumors in IMpower133. (**B**) Stacked box plots showing fraction of patients with high and low *NOTCH1* or *NOTCH2* tumors among NE-enriched and non-NE-enriched subsets in IMpower133. (**C**) Reanalysis of RNA-Seq data from Ireland et al. (29) showing expression of *Notch1*, *Notch2*, and *Rest* at multiple time points in RPM cells grown in culture. RPM cells were derived from a *Myc*-driven SCLC mouse model (*Rb1^fl/fl^*;*Trp53^fl/fl^*; Lox-Stop-Lox [LSL]-*Myc^T58A^*). (**D**) Volcano plot highlighting *Notch1* and *Notch2* with KP1 *Rest* overexpression; data from Shue et al. (25).

Given our data indicating a specific association between *NOTCH1* expression*,* but not *NOTCH2* expression, and ICB survival in SCLC, we next sought to elucidate potential differences between *NOTCH1* and *NOTCH2*, as these Notch paralogs have been previously reported to have similar functions in SCLC as tumor suppressors (23) and drivers of NE to non-NE transdifferentiation (24, 25). Using the IMpower133 dataset, we first compared expression of *NOTCH1* and *NOTCH2* in the NE-enriched (NMF1/2/3) and non-NE-enriched (NMF4) subsets. We found *NOTCH*2 to be one of the most significantly enriched genes within the non-NE-enriched subset (Figure 3A), along with *MYC* and *REST*, as previously reported by Nabet et al. (18). Surprisingly, compared with *NOTCH2*, *NOTCH1* was less upregulated in the non-NE-enriched subset (Figure 3, A and B). NE genes were also less downregulated than expected among *NOTCH1*-high NE-enriched tumors (Supplemental Figure 6A) compared with the complete downregulation of NE genes evident in our NOTCH1-activated preclinical models (Supplemental Figure 6B). The fraction of high-*NOTCH2*-expressing tumors in the non-NE-enriched subset was also greater than the fraction of high-*NOTCH1-*expressing tumors (Figure 3B). To validate these results, we performed differential gene expression analysis between NE-enriched and non-NE-enriched subsets among a combined cohort of limited-stage SCLC tumors (23, 31) and similarly found *NOTCH2*, but not *NOTCH1*, to be enriched among the non-NE-enriched subset (excluding *POU2F3*-high tumors) (Supplemental Figure 6C). We next reanalyzed RNA-Seq data generated from Ireland et al. (29), who showed that Myc activation reprograms NE cell fate through Notch signaling in a SCLC murine model. Upon Myc activation in this model, we observed little to no upregulation of *Notch1*, whereas *Notch2* and *Rest* were highly upregulated (Figure 3C). Similarly, reanalysis of RNA-Seq data of *Rest* overexpression in the KP1 SCLC murine cell line (25) showed significant upregulation of *Notch2*, but not *Notch1* (Figure 3D). In sum, these data suggest that *NOTCH1* has a distinct pattern of regulation and expression apart from *NOTCH2* and *REST* in SCLC.

### NOTCH1 reverses silencing of MHC class I and antigen presentation in SCLC.

**Figure 4:**
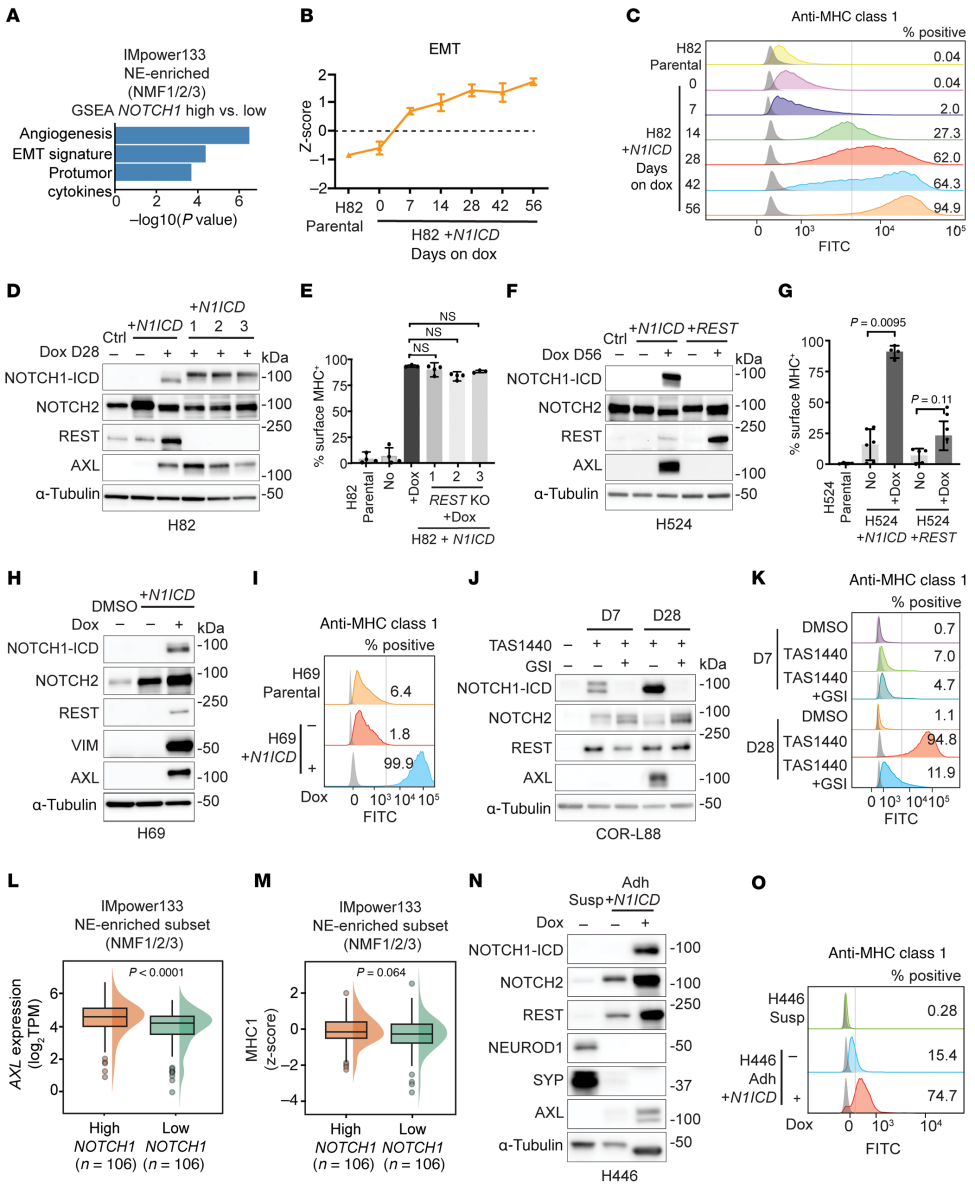
NOTCH1 reverses silencing of MHC class I and antigen presentation in SCLC. (**A**) Gene set enrichment analysis of high- compared with low-*NOTCH1*-expressing tumors in the NE-enriched subset of IMpower133. (**B**–**E**) *N1ICD* overexpression time course (0 to ≤56 days) in H82 cells with or without *REST* KO. (**B**) EMT signature (*z* scored) at the indicated time points as determined by RNA-Seq. (**C**) Flow cytometry histograms assessing cell-surface MHC class I expression at the indicated time points. (**D**) Immunoblot analysis of the indicated proteins. Three single-cell KO clones are shown. (**E**) Quantification of cell-surface MHC class I expression (data representative of *n* = 3 independent experiments). (**F** and **G**) Long-term (56 days) *N1ICD* and *REST* overexpression in H524 cells. (**F**) Immunoblot analysis of the indicated proteins. (**G**) Quantification of cell-surface MHC class I expression (data representative of *n* = 3 independent experiments). (**H** and **I**) Long-term (>56 days) overexpression of *N1ICD* in H69 cells. (**H**) Immunoblot analysis of the indicated proteins. (**I**) Flow cytometry assessing cell-surface MHC class I expression (data representative of *n* = 3 independent experiments). (**J** and **K**) Short-term (7 days) and/or long-term (28 days) treatment of COR-L88 cells with DMSO, TAS1440, and TAS1440 plus GSI (BMS-708163, 2 μM) as indicated (data representative of *n* > 3 independent experiments). (**J**) Immunoblot analysis of the indicated proteins. (**K**) Flow cytometry assessing cell-surface MHC class I expression. (**L**) *AXL* expression and (**M**) MHC class I signature (*HLA-A*, *HLA-B*, *HLA-C*, *B2M*, *TAP1*, *TAP2*, *TAPBP*) stratified by *NOTCH1* expression among the NE-enriched subset of IMpower133. (**N**) Immunoblot analysis of the indicated proteins in H446 suspension, adherent, and H446 adherent *N1ICD-*overexpressed cells (56 days). (**O**) Flow cytometry assessing cell-surface MHC class I expression. For flow cytometry graphs, shaded gray histograms represent unstained controls for each condition. Positive cells are shifted to the right of the gray vertical line. *P* values were calculated using an unpaired 2-tailed Student’s *t* test. *P* values less than 0.05 were considered significant.

Given the significant association between high *NOTCH1* expression and first-line ICB survival, we next assessed for potential mechanisms by which NOTCH1 signaling may mediate immune response by performing gene set enrichment analysis between high- and low-*NOTCH1*-expressing tumors within the NE-enriched subset of IMpwer133. Using signatures developed to predict pan-cancer response to immunotherapy (32), we found angiogenesis, epithelial-mesenchymal transition (EMT), and protumor cytokines to be the most significantly enriched pathways in high compared with low-*NOTCH1*-expressing tumors (Figure 4A). We next explored the relationship between NOTCH1 and EMT by performing RNA-Seq across multiple time points in our previously described H82 (NEUROD1) SCLC cell line model, in which HLAs and antigen presentation machinery (APM) genes are upregulated by *NOTCH1* intracellular domain (*N1ICD*) overexpression (20). We found that *N1ICD* overexpression increased EMT over time in H82 cells (Figure 4B), consistent with a model of EMT as a transitional, rather than binary, process (33). Cell-surface MHC class I expression also increased over time with *N1ICD* overexpression in concordance with EMT (Figure 4C). To further understand how NOTCH1 signaling might regulate EMT, APM and cell-surface MHC class I expression, we knocked out *REST* — a downstream Notch signaling gene known to regulate cell fate in SCLC — in H82 cells (24, 25). However, with *REST* KO and *N1ICD* overexpression, we did not observe significant differences in EMT by RNA-Seq (Supplemental Figure 7A) or the EMT marker AXL (Figure 4D), nor was there a significant change in cell-surface MHC class I expression (Figure 4E) or APM gene expression (Supplemental Figure 7B). We then directly compared NOTCH1 with REST in driving EMT and APM in SCLC by individually overexpressing *N1ICD* and *REST* in H524 (NEUROD1) cells with minimal endogenous expression of either of these proteins. As in H82 cells, long-term overexpression of *N1ICD* in H524 cells induced EMT and AXL expression, but long-term overexpression of *REST* did not (Figure 4F and Supplemental Figure 7C). H524 *N1ICD*-overexpressed cells also had significantly higher cell-surface MHC class I expression (Figure 4G) and higher APM gene expression (Supplemental Figure 7D) than H524 *REST* overexpressed cells indicating that NOTCH1 was more effective in driving EMT and upregulating antigen presentation than REST. Further supporting these data, *N1ICD* overexpression in H69 (ASCL1) cells led to significant upregulation of EMT as well as increased cell-surface MHC class I and APM gene expression (Figure 4, H and I, and Supplemental Figure 7, E and F).

Given that Notch signaling is dose dependent and *N1ICD* overexpression may not represent normal physiologic N1ICD levels (34), we next used pharmacologic activation of Notch signaling through LSD1 inhibition (35) as an orthogonal approach to assess the relationship among NOTCH1, EMT, and antigen presentation in SCLC. Consistent with prior reports by Hiatt et al. (36) and Nguyen et al. (37), short-term (7 days) treatment with a potent, reversible LSD1 inhibitor, TAS1440 (Machida et al., manuscript in preparation), broadly activated Notch signaling (i.e., expression of NOTCH1, NOTCH2, and REST) and modestly upregulated cell-surface MHC class I but did not substantially induce AXL in COR-L88 (ASCL1) cells (Figure 4, J and K). Gamma-secretase inhibition (GSI), which has been used to block oncogenic NOTCH1 signaling in T cell acute lymphoblastic leukemia (29, 35), did not alter the modest upregulation of cell-surface MHC class I with short-term TAS1440 treatment (Figure 4K). In contrast, we observed significant induction of EMT and profound upregulation of surface MHC class I with long-term (28 days) Notch activation (Figure 4, J and K, and Supplemental Figure 7G). Blocking NOTCH1 signaling with concurrent GSI and LSD1 treatment led to partial induction of EMT (Supplemental Figure 7G) and only modest upregulation of cell-surface MHC class I (Figure 4, J and K). Bulk and single-cell RNA-Seq similarly showed strong upregulation of APM gene transcription with long-term Notch activation (Supplemental Figure 7, H and I). MHC class I mass spectrometry analysis demonstrated a significant increase in cell-surface MHC–bound peptides in long-term TAS1440- compared with long-term TAS1440 plus GSI–treated cells (Supplemental Figure 7J). Consistent with our preclinical models, we observed significantly higher expression of *AXL* and higher expression of MHC class I–related genes among high- compared with low-*NOTCH1*-expressing NE-enriched tumors in IMpower133 (Figure 4, L and M).

Last, we analyzed expression of NOTCH1, NOTCH2, and REST within NE and non-NE populations of the H446 (NEUROD1) cell line (38, 39) to assess whether these proteins may be coregulated. As expected, we observed little to no expression of NOTCH1, NOTCH2, or REST and high expression of NE proteins in H446 suspension cells (Figure 4N). Interestingly, NOTCH2 and REST, rather than NOTCH1-ICD, were highly expressed in non-NE H446 adherent cells, with low concurrent expression of AXL and cell-surface MHC class I (Figure 4, N and O). Overexpression of *N1ICD* in the non-NE H446 adherent cells led to upregulation of AXL and cell-surface MHC class I, consistent with our previously described *N1ICD* overexpression models (Figure 4, N and O). Thus, our results demonstrate that NOTCH1 signaling was a key driver of MHC class I and antigen presentation in SCLC.

### Notch signaling drives the immunogenicity of SCLC.

**Figure 5:**
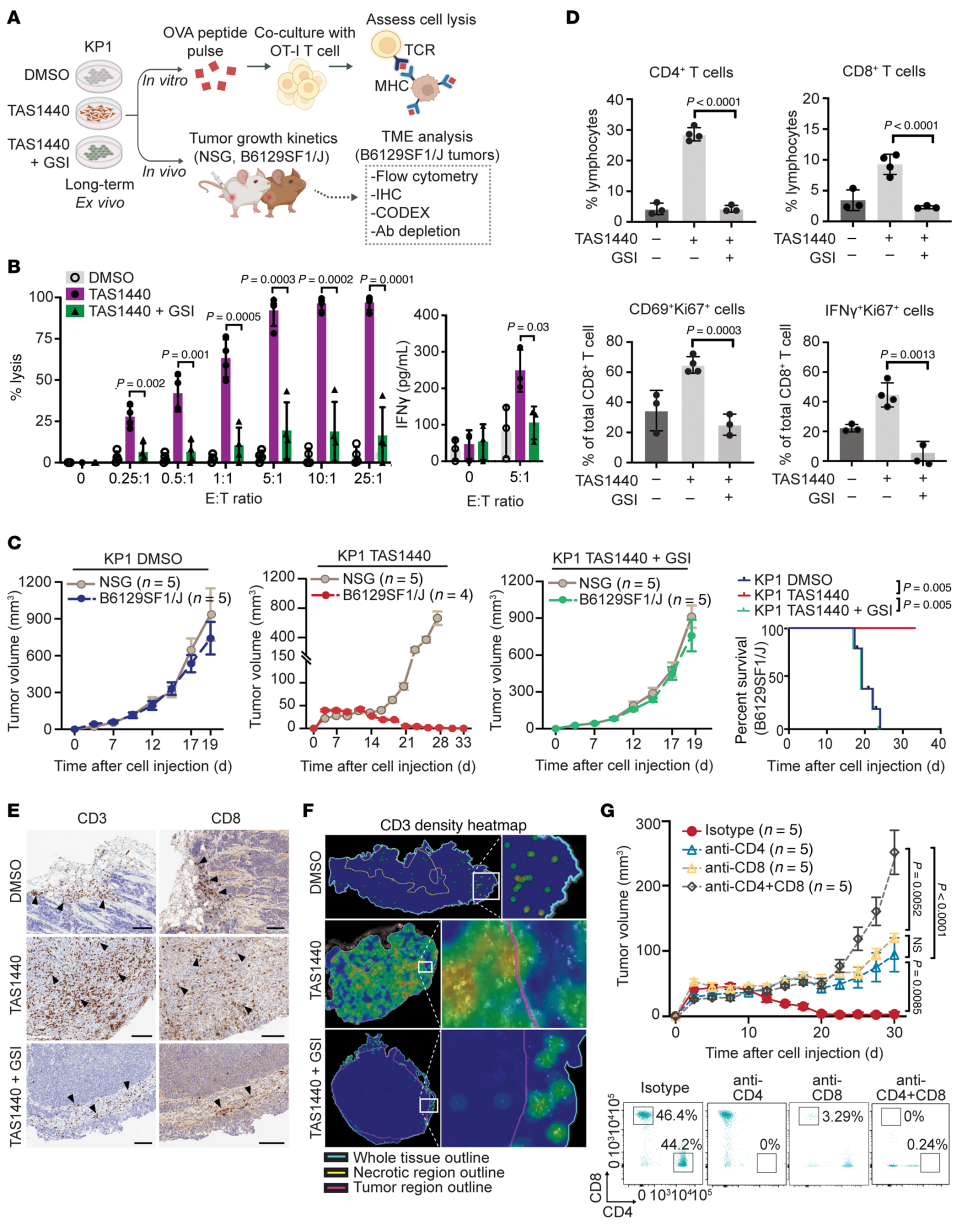
Notch signaling reprograms SCLC tumors from immune-excluded to immune-inflamed through increased T cell infiltration and activation. (**A**) Schematic of in vitro and in vivo experiments. (**B**) Percentage lysis and IFN-γ concentration in supernatants of KP1 cells cocultured with OT-I T cells for 3 days after pulsing with OVA peptide. E, effector (OT-I T cells); T, target (KP1 cells) (data representative of *n* = 3 independent experiments). (**C**) Tumor growth curves and survival of KP1 allografts in B6129SF1/J immunocompetent and NSG immunocompromised mice (data representative of *n* = 2 independent experiments). (**D**–**F**) Tumor microenvironment analysis of KP1 allograft tumors in B6129SF1/J immunocompetent mice 11 days after subcutaneous inoculation. (**D**) Flow cytometry assessing tumor T cells. (**E**) CD3^+^ and CD8^+^ T cell IHC. Arrowheads point to T cell clusters. Scale bars: 100 μm. (**F**) Spatial heatmap of CD3^+^ T cells analyzed by CODEX. (**G**) Tumor growth curves of KP1 TAS1440 allografts in B6129SF1/J immunocompetent mice with T cell depletion (upper panel). Isotype, CD4^+^, and CD8^+^ T cell depletion (*n* = 1 independent experiment). Combined CD4^+^ and CD8^+^ T cell depletion (*n* = 2 independent experiments). Flow cytometric analysis confirming T cell depletion in splenocytes (lower panel). *P* values were calculated using an unpaired 2-tailed Student’s *t* test or using a log-rank test. *P* values less than 0.05 were considered significant. Error bars in tumor growth curves (**C** and **G**) represent SEM.

Next, we sought to determine whether NOTCH1 could drive antitumor immune response in SCLC. To do this, we treated the well-established KP1 SCLC syngeneic mouse cell line (40–42) long-term ex vivo with and without TAS1440 and TAS1440 plus GSI (Figure 5A). We first measured cell growth after TAS1440 treatment at 7 days and 28 days and found no significant growth inhibition compared with the DMSO-, TAS1440 plus GSI–, and *Notch1*-KO–treated cells (Supplemental Figure 8, A and B). As in our human SCLC cell line model, long-term KP1 TAS1440–treated cells upregulated Notch signaling, induced EMT based on increased expression of Vim and the cell surface-marker Cd44, increased cell-surface MHC class I, and increased APM gene expression (Supplemental Figure 9, A–C). Blocking active Notch signaling with addition of a GSI to TAS1440 attenuated these observed phenotypes (Supplemental Figure 9, A–C). Given the strong increase in cell-surface MHC class I expression with Notch activation, we assessed whether Notch activation could induce T cell–mediated cytotoxicity by pulsing KP1 cells with OVA peptide (SIINFEKL), then coculturing them with OVA peptide–specific, i.e., OT-I, T cells. TAS1440-treated KP1 cells showed significantly greater cell lysis compared with TAS1440 plus GSI–treated cells (Figure 5B). Moreover, OT-I T cell coculture with TAS1440–treated cells induced greater T cell activation, as evidenced by higher T cell cytokine IFN-γ production, than coculture with TAS1440 plus GSI–treated cells (Figure 5B).

We next assessed the immunogenicity of Notch-driven SCLC by subcutaneously inoculating ex vivo treated KP1 cells (DMSO, TAS1440, and TAS1440 plus GSI) into both immunocompromised NSG and immunocompetent B6129SF1/J mice (Figure 5C). All KP1 cells induced tumors in immunocompromised mice. In contrast, tumors formed from TAS1440-treated KP1 cells (hereafter referred to as KP1 TAS1440 tumors) regressed over time in immunocompetent mice. However, tumors formed from DMSO and TAS1440 plus GSI–treated KP1 cells (hereafter referred to as KP1 DMSO and TAS1440 plus GSI tumors) continued to grow (Figure 5C). To validate these results, we repeated this experiment using KP3 cells, another well-validated SCLC syngeneic mouse model (40–42). Like KP1 cells, KP3 TAS1440 cells regressed over time in immunocompetent mice, whereas they induced tumors in immunocompromised mice (Supplemental Figure 9D). KP3 DMSO and TAS1440 plus GSI cells grew in both immunocompetent and immunocompromised mice, but they grew more slowly in immunocompetent mice, suggesting a partial immune response (Supplemental Figure 9D). Overall, these data underscore the role of Notch signaling in regulating SCLC in vivo immunogenicity.

Given these data, we next hypothesized that active Notch signaling may also be an underlying mechanism for in vivo tumor regression of adherent SCLC syngeneic mouse cells, as reported by Mahadevan et al. (17). To test this possibility, we generated adherent KP1 cells (KP1-A cells) by long-term culture, which we confirmed were of the same origin as parental suspension KP1 cells (Supplemental Figure 9E). As expected, KP1-A cells showed strong evidence of EMT (based on high Cd44 cell-surface expression) as well as high MHC class I cell-surface expression (Supplemental Figure 9F). In contrast, concurrent long-term culture of KP1-A cells with a GSI, which blocked Notch1 signaling (Supplemental Figure 9G), hindered upregulation of EMT and cell-surface MHC class I (Supplemental Figure 9F). Crucially, tumors formed from KP1-A cells, but not from KP1-A plus GSI cells, regressed in immunocompetent mice (Supplemental Figure 9H). Taken together, these data demonstrate that Notch signaling is a key mechanism driving in vivo SCLC antitumor immune responses.

### Notch signaling reprograms SCLC tumors from immune excluded to immune inflamed through increased T cell infiltration and activation.

The robust antitumor immune responses induced by Notch signaling in our SCLC syngeneic mouse models prompted us to evaluate the tumor microenvironment of KP1 DMSO, TAS1440, and TAS1440 plus GSI tumors (Figure 5A). Using flow cytometry, we found significant enrichment of CD4^+^ and CD8^+^ T cells in KP1 TAS1440 compared with KP1 TAS1440 plus GSI tumors (Figure 5D). Although there was less robust enrichment of CD8^+^ compared with CD4^+^ T cells, KP1 TAS1440 tumors had significantly more activated effector CD8^+^ T cells than KP1 TAS1440 plus GSI tumors (Figure 5D). Strikingly, KP1 DMSO and KP1 TAS plus GSI tumors were immune excluded, with CD3^+^ and CD8^+^ T cells restricted predominantly to the tumor margin, whereas KP1 TAS1440 tumors were immune inflamed, with abundant infiltration of CD3^+^ and CD8^+^ T cells within the interior of the tumor (Figure 5E), which was also evident in the KP1-A model (Supplemental Figure 9I). CODEX analysis concordantly revealed a large increase in CD3^+^ T cell density deep in the tumor core in KP1 TAS1440 tumors compared with KP1 DMSO and TAS1440 plus GSI tumors (Figure 5F).

As the effector functions of CD8^+^ T cells are known to be supported by the presence of CD4^+^ T cells (43), we performed in vivo antibody depletion of CD4^+^ and/or CD8^+^ T cell subsets in mice with KP1 TAS1440 tumors. Depletion of either CD4^+^ or CD8^+^ T cells resulted in tumor growth, whereas isotype-treated KP1 TAS1440 tumors regressed (Figure 5G). Depletion of both T cell subsets led to pronounced tumor growth (Figure 5G), providing evidence that tumor-infiltrating CD4^+^ and CD8^+^ T cells both have a critical role in driving antitumor immune responses of Notch-driven SCLC tumors.

### Notch1 is critical for the immunogenicity of SCLC.

**Figure 6:**
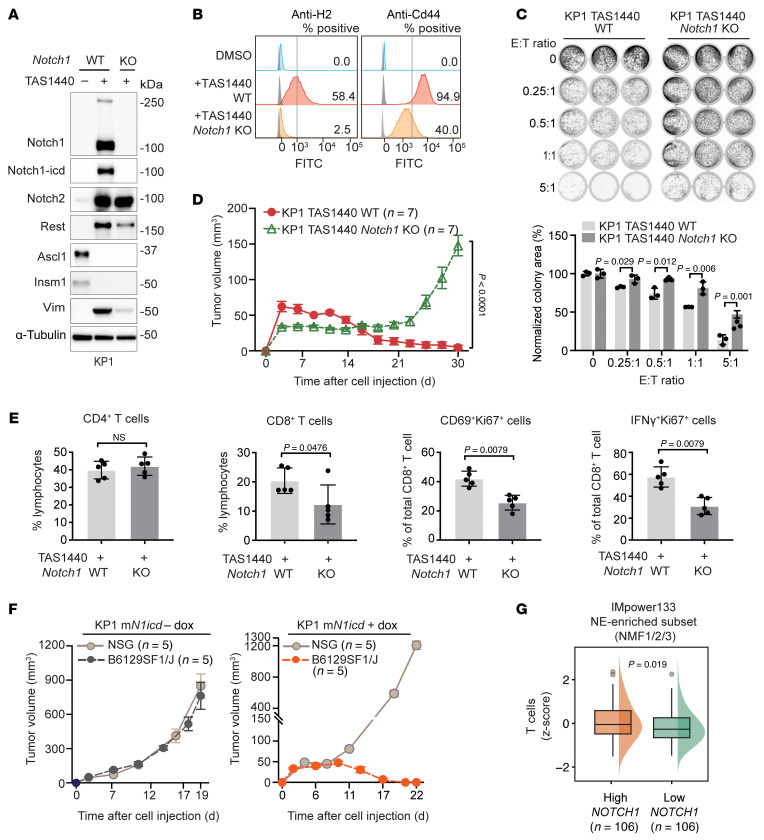
Notch1 is the critical driver of the immunogenicity of SCLC. (**A**–**E**) KP1 SCLC mouse cells with or without *Notch1* KO treated long-term (>28 days) with TAS1440. (**A**) Immunoblot analysis of Notch signaling, NE, and EMT proteins. (**B**) Flow cytometry histograms assessing cell-surface H2 and Cd44 expression. Shaded gray histograms represent unstained controls for each condition. Positive cells have an H2 or Cd44 signal higher than the referenced gray vertical line. Data representative of *n* = 3 independent experiments. (**C**) T cell–mediated killing assay showing remaining tumor cells assessed by crystal violet staining after coculture of KP1 cells with OT-I T cells for 3 days following OVA peptide pulsing. E, effector (OT-I T cells); T, target (KP1 cells). Colony area for each E:T condition was quantified and normalized to the no–T cell control (E:T = 0) within each group. (**D**) Tumor growth curves of KP1 TAS1440 allografts in B6129SF1/J immunocompetent mice and (**E**) flow cytometry T cell analysis 11 days after subcutaneous inoculation. (**F**) Notch1-icd overexpression in KP1 cells treated with doxycycline ex vivo long-term (>28 days) before subcutaneous inoculation into mice. Tumor growth curves of KP1 m*N1icd* allografts in immunocompetent and immunocompromised mice. (**G**) T cells signature stratified by *NOTCH1* expression among NE-enriched tumors in IMpower133. Error bars in tumor growth curves (**D** and **F**) represent SEM. *P* values were calculated using an unpaired 2-tailed Student’s *t* test. *P* values less than 0.05 were considered significant.

Although GSIs have been used extensively to block Notch signaling in SCLC (29, 35), these drugs have also been shown to target other membrane proteins (44, 45). Therefore, to assess the specific relationship between Notch1 and antitumor immune response in SCLC, we knocked out *Notch1* in KP1 cells and treated these cells long-term ex vivo with TAS1440 (Figure 6A). Despite similarly high expression of Notch2 and Rest and downregulation of NE proteins (Figure 6A), KP1 TAS1440 *Notch1*-KO cells had lower cell-surface MHC class I expression and decreased EMT, as evidenced by lower Vim and cell-surface Cd44 expression compared with TAS1440-treated *Notch1* WT cells (Figure 6B). Consistent with these findings, OT-I T cell killing assays demonstrated reduced cytotoxicity against KP1 *Notch1*-KO cells compared with *Notch1* WT cells following TAS1440 treatment, further supporting a critical role for Notch1 in enhancing antigen presentation and T cell–mediated killing (Figure 6C). Moreover, in immunocompetent mice, KP1 TAS1440 *Notch1*-KO cells induced tumor growth, whereas tumors induced from *Notch1* WT cells regressed (Figure 6D). Using flow cytometry, we found significant depletion of both total CD8^+^ T cells and activated CD8^+^ T cells in KP1 TAS1440 *Notch1*-KO tumors compared with *Notch1* WT tumors (Figure 6E). Moreover, tumors formed from KP1 cells with *N1icd* overexpression (Supplemental Figure 9J) regressed over time in immunocompetent mice, whereas such tumors grew in immunocompromised mice (Figure 6F). These data demonstrate that Notch1 was required to reverse silencing of antigen presentation and induce a robust CD8^+^ T cell–mediated response in SCLC. Concordantly, we observed significant enrichment of a T cell signature (32) in high- compared with low-*NOTCH1*-expressing NE-enriched tumors in IMpower133 (Figure 6G).

### NOTCH1 reverses silencing of antigen presentation in SCLC through reactivation of STING.

**Figure 7:**
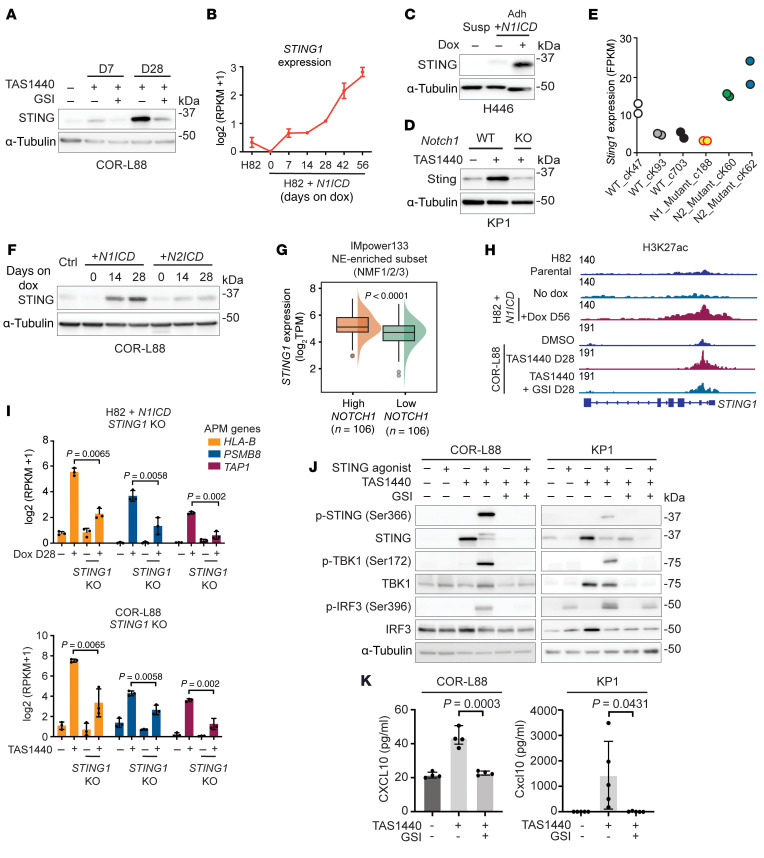
NOTCH1 reverses silencing of antigen presentation in SCLC through reactivation of STING. (**A**) Immunoblot analysis in COR-L88 cells treated either short-term (7 days) or long-term (28 days) with DMSO, TAS1440, and TAS1440 plus GSI (BMS-708163, 2 μM). (**B**) RNA-Seq expression of *STING1* at the indicated time points in H82 cells overexpressing *N1ICD*. Immunoblot analysis showing Sting expression in (**C**) H446 cells overexpressing *N1ICD* and (**D**) mouse KP1 SCLC cells treated long-term with TAS1440, with or without *Notch1* KO. (**E**) RNA-Seq expression of *Sting1* in WT, *Notch1*-KO, and *Notch2*-KO tumors from the Hong et al. (47) dataset. (**F**) Immunoblot analysis of the indicated proteins in COR-L88 cells after NOTCH1-ICD or human NOTCH2-ICD overexpression. (**G**) *STING1* expression stratified by *NOTCH1* expression among NE*-*enriched tumors in IMpower133. (**H**) Visualization of H3K27ac peaks across the 5′ *STING1* locus in H82 cells overexpressing *N1ICD* and COR-L88 cells treated with DMSO, TAS1440, and TAS1440 plus GSI (BMS-708163, 2 μM). Normalized total reads are shown in top left of each condition shown (data representative of *n* = 2 independent experiments). (**I**) RNA-Seq expression of APM genes in H82 cells overexpressing *N1ICD* and COR-L88 cells treated with DMSO, TAS1440, and TAS1440 plus GSI (BMS-708163, 2 μM) with or without *STING1* KO. (**J** and **K**) COR-L88 and/or KP1 cells treated long-term (≥28 days) with DMSO, TAS1440, and TAS1440 plus GSI (COR-L88: BMS-708163, 2 μM; KP1: DBZ, 10 μM). Data are representative of *n* = 3 independent experiments. (**J**) Immunoblot analysis of STING pathway proteins with STING agonist treatment conditions as shown (COR-L88: diABZi, 500 nM for 4 hours; KP1: MSA-2, 30 μM for 1.5 hours). (**K**) CXCL10 quantification in cell supernatants by ELISA. *P* values were calculated using an unpaired 2-tailed Student’s *t* test. *P* values less than 0.05 were considered significant.

We next sought to decipher potential mechanism(s) by which NOTCH1 reverses immune suppression in SCLC by performing bulk RNA-Seq and gene set enrichment analysis (GSEA) between TAS1440- and TAS1440 plus GSI–treated COR-L88 and KP1 cells. The immune system gene set was a top differentially enriched pathway, with interferon-inducible genes highly upregulated in TAS1440- compared with TAS1440 plus GSI–treated cells (Supplemental Figure 10A). Findings were similar in H82 cells with and without *N1ICD* overexpression (Supplemental Figure 10B). We therefore postulated that expression of STING, a known regulator of interferon and cytokine production (46), may be higher in NOTCH1-driven cells. Indeed, we observed upregulation of STING in long-term (28 days) NOTCH1-driven COR-L88 cells (Figure 7A and Supplemental Figure 10C) but minimal STING upregulation in short-term (7 days) NOTCH1-driven COR-L88 cells (Figure 7A). *STING1* expression increased concurrently over time with EMT in H82 cells with *N1ICD* overexpression with or without *REST* KO (Figure 7B and Supplemental Figure 10D). We also observed upregulation of STING after *N1ICD* overexpression in non-NE H446 adherent cells (Figure 7C) and lower expression of Sting in KP1 TAS1440 *Notch1*-KO compared with WT cells (Figure 7D). Reanalysis of RNA-Seq data from Hong et al. (47) similarly showed low *Sting1* expression among murine SCLC tumors with *Notch1* KO (N1_Mutant_c188) in contrast to *Notch2-*KO tumors (N2_Mutant_cK60 and cK62) (Figure 7E). To further investigate potential differences between NOTCH1 and NOTCH2, we overexpressed *N1ICD* and *N2ICD* in COR-L88 cells (Supplemental Figure 10E) and found that *N1ICD* overexpression led to more robust STING protein upregulation than did *N2ICD* overexpression (Figure 7F). Additionally, *N1ICD* overexpression upregulated the EMT marker VIM to a greater extent than *N2ICD* overexpression, suggesting distinct roles for NOTCH1 and NOTCH2 in regulating EMT and tumor-intrinsic STING expression (Supplemental Figure 10F). Importantly, there was significantly higher *STING1* expression in high- compared with low-*NOTCH1*-expressing NE-enriched tumors in IMpower133 (Figure 7G).

Next, as *STING1* expression has been shown to be repressed across many cancers through epigenetic mechanisms (48), we used CellMiner-SCLC (49) and found a significant correlation between *STING1* expression and enrichment of H3K27ac at the *STING1* promoter region (Supplemental Figure 10G). We then performed H3K27ac ChIP-Seq and found enhancement of H3K27ac occupancy at the 5′ end of the *STING1* locus in NOTCH1-driven H82 and TAS1440-treated COR-L88 cells (Figure 7H). Moreover, APM gene expression was lower in both NOTCH1-driven H82 and TAS1440-treated COR-L88 cells with *STING1* KO compared with *STING1* WT, suggesting that *STING1* expression is critical for NOTCH1-induced antigen presentation (Figure 7I and Supplemental Figure 10H).

In addition to STING upregulation, we also observed activation of the STING pathway in both TAS1440-treated COR-L88 and KP1 cells with STING agonism, as evidenced by serine 366 phosphorylation of STING and phosphorylation of the STING downstream molecules TBK1 and IRF3 (Figure 7J). CXCL10, a downstream STING pathway chemokine, was also significantly elevated in TAS1440-compared with TAS1440 plus GSI–treated COR-L88 and KP1 cells (Figure 7K). Collectively, these data support NOTCH1 as a key mechanism driving epigenetic upregulation of STING and STING pathway activation in SCLC.

### STING agonism combined with anti–PD-L1 therapy induces durable, complete antitumor immune responses in Notch-driven SCLC.

**Figure 8:**
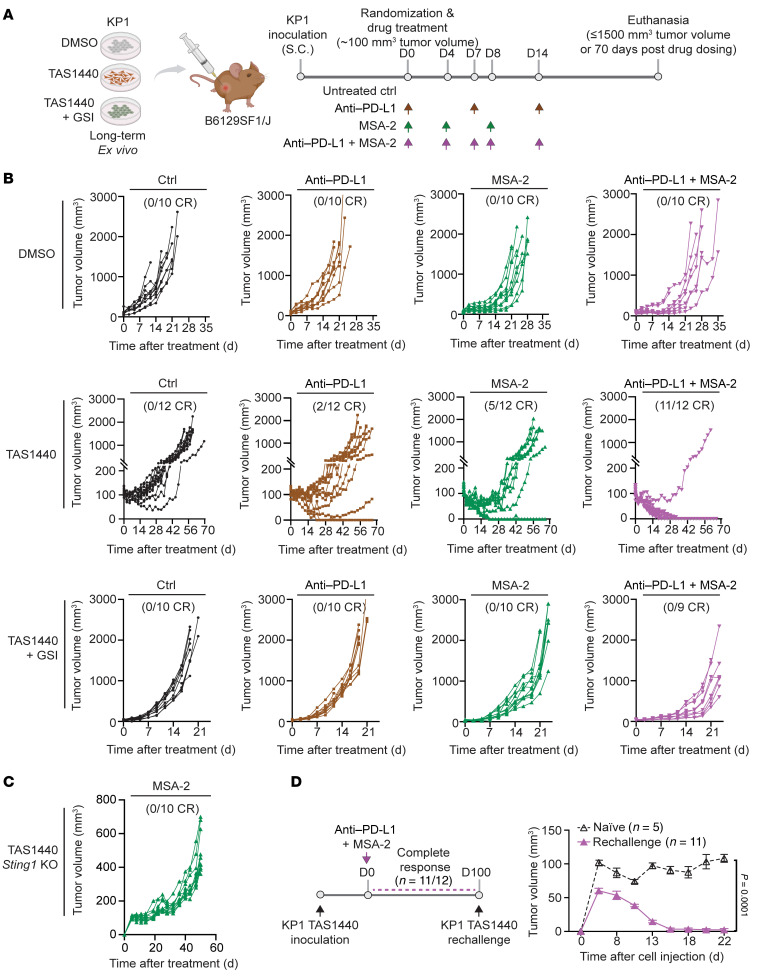
STING agonism combined with anti–PD-L1 therapy induces durable, complete antitumor immune responses in Notch-driven SCLC. (**A**) Schematic of in vivo experiment. (**B**) Tumor growth curves of KP1 allografts treated in vivo with control (Ctrl; black), anti–PD-L1 (brown), MSA-2 (green), or anti–PD-L1 + MSA-2 (pink). Each line represents an individual mouse within a given experiment. The number of mice with complete responses within each cohort is shown in parentheses. (**C**) Tumor growth curves of KP1 allografts inoculated with KP1 TAS1440 *Sting1*-KO cells treated in vivo with MSA-2. Each line represents an individual mouse within a given experiment. The number of mice with complete tumor regressions within the overall cohort size is shown in parentheses above the curves. (**D**) Tumor growth curves of KP1 TAS1440 allografted mice with complete responses to anti–PD-L1 + MSA-2 combination treatment rechallenged with KP1 TAS1440 cells. Error bars in growth curves represent SEM.

As NOTCH1 activation increased *STING1* expression and STING pathway sensitivity, we next hypothesized that STING agonism may augment in vivo antitumor immune responses in Notch-driven SCLC tumors. To this end, we administered MSA-2, a non-nucleotide STING agonist (50), with and without anti–PD-L1 therapy, to immunocompetent B6129SF1/J mice after subcutaneously inoculating ex vivo treated KP1 cells (DMSO, TAS1440, and TAS1440 plus GSI) (Figure 8A). For the KP1 TAS1440 cohort, we used a cell number higher than in prior experiments in order to consistently induce tumor growth (Supplemental Figure 11A).

Administration of MSA-2 led to complete tumor regression in the subset of mice bearing KP1 TAS1440 tumors (*n* = 5 of 12), but not in the mice bearing KP1 DMSO (*n* = 0 of 10) or KP1 TAS1440 plus GSI (*n* = 0 of 10) tumors (χ^2^ value, *P* = 0.04) (Figure 8B), suggesting that only Notch-driven tumors are sensitive to STING agonism. Furthermore, mice bearing KP1 TAS1440 *Sting1*-KO tumors treated with MSA-2 did not show complete tumor regression (*n* = 0 of 10), suggesting that tumor intrinsic STING is critical for sensitivity to STING agonism in Notch-driven tumors (Figure 8C and Supplemental Figure 11B).

Mice bearing KP1 TAS1440 tumors also showed a better response to anti–PD-L1 therapy alone, with a significant reduction in average tumor volume, compared with untreated KP1 TAS1440 tumor–bearing mice (Supplemental Figure 11C). There was no significant reduction in average tumor volume between anti–PD-L1–treated and untreated mice with KP1 DMSO– and KP1 TAS1440 plus GSI–bearing tumors (Supplemental Figure 11C). Although the difference was not significant, a subset of mice bearing KP1 TAS1440 tumors (*n* = 2 of 12) had complete responses with anti–PD-L1 therapy, whereas no complete responses were seen with anti–PD-L1 therapy in mice bearing KP1 DMSO (*n* = 0 of 10) or KP1 TAS1440 plus GSI tumors (*n* = 0 of 10) (χ^2^ value, *P* = 0.48) (Figure 8B). Strikingly, MSA-2 treatment combined with anti–PD-L1 therapy led to durable and complete responses in nearly all mice with KP1 TAS1440–bearing tumors (*n* = 11 of 12) (Figure 8B), which were not evident in mice with KP1 DMSO– (*n* = 0 of 10) or KP1 TAS1440 plus GSI–bearing (*n* = 0 of 9) tumors (χ^2^ value, *P* < 0.0001) (Figure 8B). Mice with KP1 TAS1440–bearing tumors treated with MSA-2 and anti–PD-L1 also developed enduring antitumor immunity, as they rejected tumor rechallenge (Figure 8D). Therefore, we conclude that STING agonism greatly potentiated the effects of PD-L1 blockade in Notch-driven SCLC tumors.

### Intertumor and intratumor heterogeneity of active NOTCH1 signaling in SCLC.

**Figure 9:**
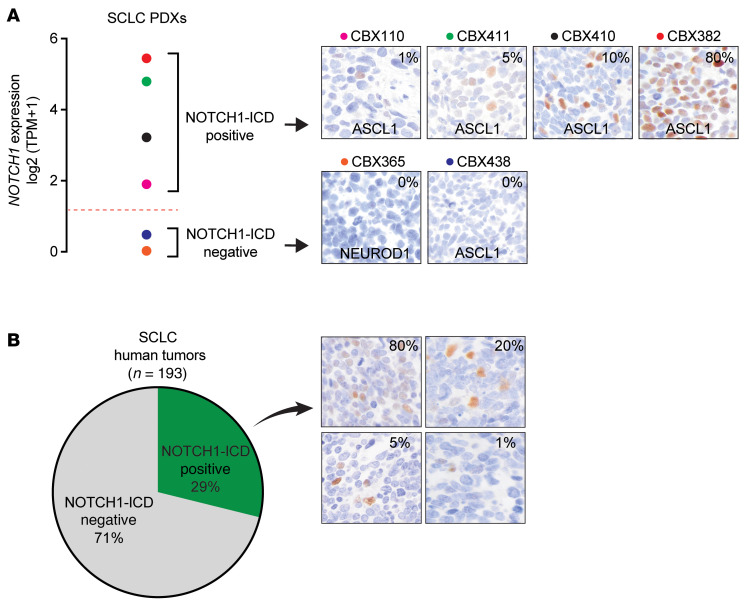
NOTCH1 signaling is active in SCLC. (**A**) *NOTCH1* expression by RNA-Seq and NOTCH1-ICD by IHC in 6 SCLC PDXs. Percentage of positive NOTCH1-ICD tumor cells (1%, 5%, 10%, or 80%) and defined subset (ASCL1 or NEUROD1) are shown. (**B**) The percentage of NOTCH1-ICD IHC-positive samples in a cohort of 193 SCLC human tumors. IHC images were taken at ×40 and show tumors with variable percentages of positive NOTCH1-ICD tumor cells.

Last, we sought to determine the prevalence of active NOTCH1 signaling in SCLC and assess for the potential utility of NOTCH1 as a clinical biomarker through IHC staining of the intracellular domain (ICD) of NOTCH1 in SCLC preclinical models and patient tissues. NOTCH1-ICD was present by immunoblotting in 24% (*n* = 10 of 42) of ASCL1^+^ SCLC cell lines (Supplemental Figure 12, A and B) and by IHC in a sample of ASCL1^+^ treatment–naive SCLC patient–derived xenografts with high, but not low, *NOTCH1* expression (Figure 9A). As there was insufficient tissue for IHC staining in the IMpower133 cohort, we performed NOTCH1-ICD IHC on 193 primary SCLC tissues with associated molecular subtyping (51). We found positive NOTCH1-ICD IHC staining in 29% (*n* = 56 of 193) of SCLC tissues (Figure 9B), including 29% (*n* = 44 of 154) of ASCL1^+^ SCLC tumors (Supplemental Figure 12C), demonstrating intertumor heterogeneity of active NOTCH1 signaling in SCLC. Moreover, we found substantial intratumor heterogeneity of NOTCH1-ICD expression, ranging from 1% and 80% of tumor cells (Figure 9B), consistent with data from a prior IHC study of NOTCH1 in SCLC (52) and the known intratumor heterogeneity of Notch signaling in SCLC mouse models (24). In non-ASCL1 SCLC cell lines and tumors, positive NOTCH1-ICD staining was also evident (Supplemental Figure 12, A–C), but limited sample size precluded definitive assessment of active NOTCH1 signaling in these subsets. Last, we reanalyzed available scRNA-Seq data from 2 cohorts of SCLC human tumors (53, 54) and found additional evidence of intratumor heterogeneity of *NOTCH1* expression in both *ASCL1*- and *NEUROD1*-enriched tumors (Supplemental Figure 13, A–C). In sum, we propose a model by which MYC-NOTCH2-REST can promote the evolution of an immune-inflamed, non-NE-enriched, ICB-nonresponsive subset, whereas NOTCH1 activation induces intratumor heterogeneity of ASCL1 and NEUROD1 NE-enriched SCLC with high EMT, STING, and CD8^+^ T cell infiltration, thereby favoring survival with ICB.

## Discussion

The recent elucidation of SCLC heterogeneity through transcriptomic profiling has raised the possibility of therapeutically targeting subsets of SCLC patients (16, 18, 28, 31). Nonetheless, SCLC is currently treated as a single disease entity with no predictive biomarkers available in the clinic to guide first-line ICB treatment. In this study, we show that high expression of *NOTCH1* was strongly associated with ICB survival among SCLC patients with *ASCL1*- and *NEUROD1*-enriched tumors, the most common subsets constituting approximately 80% of all SCLC tumors. Thus, our results suggest that NOTCH1-ICD, the active signaling component of NOTCH1, should be evaluated as a predictive biomarker to guide ICB treatment in SCLC. Specifically, our results suggest that patients with SCLC patients with NOTCH1-ICD–positive ASCL1 and NEUROD1 tumors by IHC may benefit from first-line ICB with chemotherapy, whereas SCLC patients with NOTCH1-ICD–negative ASCL1 and NEUROD1 tumors by IHC may benefit from first-line chemotherapy alone or additional combinatorial strategies. Congruent with these data, unselected SCLC patients with NOTCH1-positive tumors by IHC were previously shown to have shorter survival with chemotherapy than unselected SCLC patients with NOTCH1-negative tumors (55). Practically, our data suggest that a NOTCH1-ICD IHC assay could be implemented in the clinic, as it is tumor specific, and NOTCH1-ICD is expressed in a sizable percentage (~30%) of SCLC patient tumors.

Given that all SCLC patients without contraindications receive first-line ICB combined with chemotherapy, predictive biomarkers may ultimately be most useful to select for patients with SCLC who could benefit from additional immunotherapy agents combined with ICB. The addition of either anti-TIGIT (56) or anti–CTLA-4 (7) immunotherapy to ICB did not show additional benefit, demonstrating the challenge of conducting large trials in SCLC without a biomarker-selected population. Our data suggest that SCLC patients with ASCL1 and NEUROD1 NOTCH1-ICD–positive tumors may be an immunotherapy-sensitive population and that the addition of a STING agonist with anti–PD-L1 therapy may enhance antitumor immune response in this population. Although cyclic dinucleotide STING agonists administered by intratumoral injection have not elicited strong clinical responses (57), newer-generation, noncyclic dinucleotide STING agonists with intravenous injection are more promising and are currently in early clinical development. Encouragingly, a recent biomarker-driven SCLC clinical trial demonstrates the feasibility of conducting future investigational studies in selected SCLC populations (58).

Our clinical analysis also uncovered a distinct relationship among NOTCH1, tumor heterogeneity, and ICB survival in SCLC. Despite the predominant NE features of both *ASCL1*- and *NEUROD1*-enriched tumors, we show that high *NOTCH1* expression delineates a subset of these tumors with higher EMT than tumors with low *NOTCH1* expression. These data demonstrate that *ASCL1*- and *NEUROD1*-enriched tumors are more heterogenous than previously appreciated and suggest NOTCH1 signaling as a new mechanism underlying the ICB survival benefit in these subsets. Supporting our findings, Nabet et al. (18) observed greater EMT among the ICB-responsive, immune-inflamed, *ASCL1*-enriched NE (NMF3) subset than the ICB-nonresponsive, immune-inflamed, non-NE-enriched (NMF4) subset. The association we observed between *NOTCH1* expression and ICB survival in the *NEUROD1*-enriched subset is particularly notable, as this subset has previously been characterized as immune “cold” and immunosuppressive (53). Additionally, the lack of association between ICB survival and expression of Notch signaling genes such as *NOTCH2* and *REST* highlights the specificity of *NOTCH1* expression in predicting ICB survival in SCLC. *REST* expression may not predict ICB survival, as it is at least partially driven by MYC (29) and enriched in the SCLC-I–non-NE subset that derives limited benefit from the addition of ICB to chemotherapy (18). Likewise, Notch2 is downstream of Myc in SCLC mouse models (29), and our analysis demonstrates *NOTCH2*, similar to *REST*, is enriched in the ICB-nonresponsive SCLC-I–non-NE subset. In total, these data demonstrate the unique role of NOTCH1 and raise the possibility of additional downstream effectors that remain to be elucidated.

We also support the clinical findings of this study by elucidating the specific role of NOTCH1 in driving EMT and immune response in ASCL1 and NEUROD1 SCLC preclinical models. Our in vitro models demonstrate that while NOTCH2 and REST could induce partial EMT, NOTCH1 upregulated EMT and APM genes including MHC class I. In vivo*,* we show that *Notch1* KO abrogated EMT, MHC class I upregulation, and CD8^+^ T cell–mediated antitumor response induced by broad activation of Notch signaling. Our reanalysis of data from Ireland et al. (29) demonstrate that the Myc-mediated cell fate switch preferentially upregulates *Notch2* and *Rest*, rather than *Notch1*. Similarly, our reanalysis of data from Shue et al. (25) suggests potential differences between Notch1 and Notch2 signaling proteins, as *Rest* overexpression upregulated *Notch2* rather than *Notch1*. Ouadah et al. (59) also demonstrated *Notch2*, not *Notch1*, as a primary marker of a NE–stem cell population that can undergo self-renewal after lung injury. Thus, our data, in the context of previous work, suggest a distinct role for NOTCH1 in driving immune response in SCLC.

We propose STING as one mechanism by which NOTCH1 drives immune response in SCLC. While STING is a known mediator of SCLC immune response (42), tumor expression of STING is low in SCLC (60), potentially limiting therapeutic targeting of this pathway. However, our finding that NOTCH1 could epigenetically restore STING pathway activity suggests that therapeutic NOTCH1 activation may be a strategy to convert typically cold immune-excluded or immune-desert SCLC tumors into “hot,” or immune-inflamed, tumors. We postulate that mesenchymal cells induced by NOTCH1 activation, though less abundant than typical epithelial cells within a given SCLC tumor, can promote an immune-inflamed tumor microenvironment through STING pathway activation. Indeed, our finding of increased *STING1* expression among high- compared with low-*NOTCH1*-expressing tumors in IMpower133 supports this concept. Furthermore, long-term survivors from both the atezolizumab and placebo arms of the IMpower133 trial were observed to have enrichment of downstream STING pathway chemokines such as CXCL10 (27). As our IHC data suggest that NOTCH1-ICD is broadly downregulated in SCLC, with only 2%–6% of SCLC patients harboring loss-of-function NOTCH1 alterations (23, 61), deciphering potential mechanisms restricting NOTCH1 expression in SCLC will be important. While our work focuses on the role of NOTCH1 in inducing tumor-intrinsic STING expression and activation, NOTCH1 has been shown to inhibit STING activation in CD4^+^ T cells via binding to the cyclic dinucleotide binding site (62). Furthermore, Hong et al. (47) observed high *Sting1* expression in SCLC mouse tumors with genetic loss of *Notch2*, but not *Notch1*. Therefore, further investigation is warranted to decipher the relationship among STING, NOTCH1, and NOTCH2, particularly in the context of different cell types.

Our study also demonstrates the importance of NOTCH1 in driving EMT in SCLC. Although NOTCH1 can drive EMT across some cancer model systems (63), the relationship between NOTCH1 and EMT in SCLC has not been well-defined. For example, one study showed that NOTCH1 activation suppresses the EMT genes Snail and Twist in SCLC but did not broadly examine the effect of NOTCH1 on EMT signatures or gene sets (64). More importantly, to our knowledge, the relationship among NOTCH1, EMT, and immune response in SCLC is not known. Rather, prior work on NOTCH1 in SCLC has largely focused on the role of NOTCH1 as a tumor suppressor (23) and as a driver of NE to non-NE transdifferentiation (24, 25). Interestingly, one study found SCLC-A and SCLC-N subsets to have mesenchymal features distinct from the non-NE subset (65), which corresponds with our finding that NOTCH1-driven EMT is specific to *ASCL1*- and *NEUROD1*-enriched tumors, rather than non-NE-enriched tumors. Further supporting our data, EMT has been associated with an immune-inflamed tumor microenvironment in SCLC (16, 18, 66) and across many other cancers (67). Given that EMT is highly context dependent (68) and composed of transition and hybrid states (33), further elucidation of the relationship between NOTCH1 and the EMT transcriptional response that may impact antitumor immunity and ultimately ICB-mediated survival in SCLC will be important.

There are several limitations to our study. As we did not directly assess the relationship between NOTCH1-ICD IHC and survival with ICB, additional retrospective and prospective data will be required to establish NOTCH1-ICD as a predictive biomarker with ICB in SCLC. Our clinical analyses also used OS as the primary outcome measure, which may not account for therapies after first-line ICB with chemotherapy. Furthermore, the lack of significant association between *NOTCH1* expression and long-term survival with ICB suggests additional therapies or variables after first-line ICB with chemotherapy may be important. Given data that inflamed cells may be enriched in chemotherapy-resistant tumors (16), further work will be required to assess whether *NOTCH1* expression changes over time in response to treatment. While we provide evidence that NOTCH1 is specifically required to upregulate antigen presentation and drive immune response in SCLC, it is possible that differences in the signal strength and duration of NOTCH1 compared with NOTCH2 may influence our NOTCH1-specific findings (69). Last, our study did not address the relationship between NOTCH1 and Notch signaling ligands such DLL3, an emerging immunotherapy target (70), in inducing EMT, STING, and immune response in SCLC.

SCLC has long been observed to have minimal expression of APM complex genes such as MHC class I (2, 3) and lack significant tumor immune infiltration (4). SCLC also has limited benefit from ICB, despite being a smoking-related cancer with a high TMB (71). In this work, we discover NOTCH1 as a potential predictive biomarker for ICB and show that NOTCH1 can drive both antigen presentation and tumor T cell infiltration in SCLC by reexpression of STING. Our results suggest that the downregulation of NOTCH1 in SCLC, previously attributed to its tumor suppressor functions (23), may be a mechanism by which SCLC avoids immune surveillance. As *NOTCH1* expression is suppressed in many NE cancers (72), activation of NOTCH1 may be a broader therapeutic strategy to elicit antitumor immune response beyond SCLC.

## Methods

### Sex as a biological variable.

Both male and female mice were used in this study. Seven-week-old B6129SF1/J female mice and OT-I transgenic male and female mice C57BL/6-Tg (TcraTcrb) 1100Mjb/J were obtained from The Jackson Laboratory. Seven-week-old male and female NSG mice were obtained from the CCR Animal Research Program. No sex-specific differences were observed in experiments that included both male and female mice. For experiments using female B6129SF1/J mice, sex as a biological variable was not directly assessed.

### Statistics.

All statistical tests between groups were unpaired 2-tailed Student’s *t* tests, unless otherwise stated, and *P* values less than 0.05 were considered statistically significant. Survival analyses were conducted using Cox proportional hazard models using the R survival package (v3.1.7). Log-rank values were reported for survival analyses. For box plots, the horizontal line represents the median, the lower and upper boundaries correspond to the first and third quartiles, and the lines extend up to 1.5 above or below the IQR (where IQR is the interquartile range, or distance between the first and third quartiles).

### Study approval.

All animal procedures reported in this study were approved by the NCI Animal Care and Use Committee (ACUC) and in accordance with federal regulatory requirements and standards. All components of the intramural NIH ACU program are accredited by AAALAC International. The IMpower133 randomized clinical trial protocol was approved by the institutional review board or independent ethics committee for each study site.

### Data availability.

Bulk RNA-Seq, scRNA-Seq, and ChIP-seq data generated in this study have been deposited in the NCBI Gene Expression Omnibus (GEO) database under accession number GSE244947. Previously published datasets reanalyzed in this study can be accessed at GEO GSE149180, GEO GSE164404, and the Human Tumor Atlas https://data.humantumoratlas.org IMpower133 clinical and RNA-Seq data are available under controlled access as described in Nabet et al. (18). Raw data for all points in graphs are reported in the Supporting Data Values file.

## Author contributions

YSK and NR designed research studies. YSK, BNC, NYS, RS, CER, LL, AAI, SL, MJV, SD, SR, NS, MJL, NK, AEB, and NR conducted experiments. YSK, BNC, NYS, RS, CER, AR, SS, MJL, NK, TA, SD, SK, HZ, MIA, YP, MV, and NR acquired and analyzed experimental data. BN, AD, VG, AC, DSS, and NR acquired and analyzed clinical data. PG, MEA, HC, MV, and NR analyzed and interpreted immunohistochemistry. YSK and BYN contributed equally to the work as YSK conducted the experiments and BYN performed the clinical analyses. In addition, YSK designed the study and wrote the manuscript with NR, and therefore is listed as first co-author. All authors reviewed the results and approved the final version of the manuscript.

## Supplementary Material

Supplemental data

Unedited blot and gel images

Supporting data values

## Version 1 07/08/2025

In-Press Preview

## Version 2 09/16/2025

Electronic publication
