# Supplementary material for: NOTCH1 reverses immune suppression in small cell lung cancer through reactivation of STING
Source: J Clin Invest. 2025 Jul 8;135(18):e185423. doi: 10.1172/JCI185423 (PMC12435836; doi:10.1172/JCI185423)
Supplement: Supplemental data [file jci-135-185423-s306.pdf]

NOTCH1 reverses immune suppression in small cell lung cancer through reactivation of STING

Yoo Sun Kim[1], Barzin Y. Nabet[2], Briana N. Cortez[1], Nai-Yun Sun[1], Robin Sebastian[1], Christophe E. Redon[1], Anagh Ray[1], Liang Liu[1], Afeez Ishola[1], Sarah Loew[1], Anjali Dhall[1], Sivasish Sindiri[3], Velimir Gayevskiy[2,4], Min-Jung Lee[1], Shraddha Rastogi[1], Nahoko Sato[1], Noemi Kedei[5], Thorkell Andresson[6], Sudipto Das[6], Suresh Kumar[1], Alan E. Bers[1], Hongliang Zhang[1], Alberto Chiappori[7], Priyanka Gopal[8], Mohamed E. Abazeed[8], Haobin Chen[9], Mirit I. Aladjem[1], Yves Pommier[1], Moises J. Velez[10], David S. Shames[2], and Nitin Roper[1]

[1]Developmental Therapeutics Branch, Center for Cancer Research, National Cancer Institute, Bethesda, MD, USA

[2]Genentech Inc., South San Francisco, CA, USA

[3]Surgery Branch, Center for Cancer Research, National Cancer Institute, Bethesda, MD, USA

[4]Rancho Biosciences, San Diego, CA, USA

[5]Collaborative Protein Technology Resource, Center for Cancer Research, National Cancer Institute, Bethesda, MD, USA

[6]Protein Characterization Laboratory, National Cancer Institute, Bethesda, MD, USA

[7]Thoracic Oncology Program, Moffitt Cancer Center, Tampa, FL, USA

[8]Department of Radiation Oncology, Northwestern University, Feinberg School of Medicine, Chicago, IL, USA

[9]Division of Oncology, Department of Medicine, Washington University, St. Louis, MO, USA

[10]Department of Pathology and Laboratory Medicine, University of Rochester, Rochester, NY, USA

This PDF file includes:

Supplemental Figures 1-14

Supplemental Methods

Supplemental Figures

Supplemental Figure 1

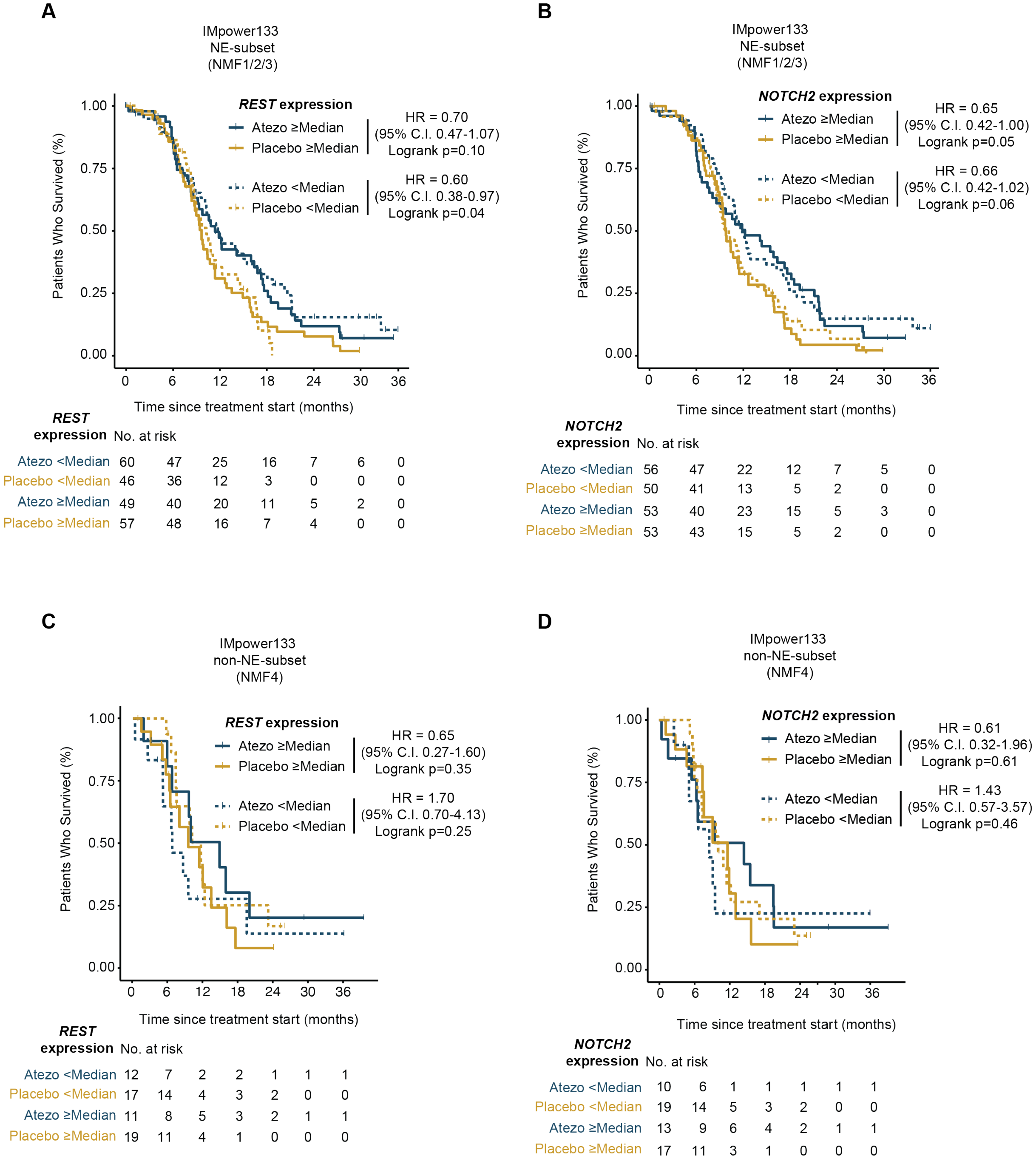

Supplemental Figure 1. No significant differences in overall survival with the addition of atezolizumab to first-line chemotherapy among NE-enriched and non-NE-enriched subsets of extensive-stage SCLC patients in the IMpower133 clinical trial based on *NOTCH2* and *REST* expression. Kaplan–Meier estimates of overall survival among NE-enriched (NMF1/2/3) SCLC patients in the atezolizumab and placebo treatment groups of IMpower133 stratified by **(A)** *REST* and **(B)** *NOTCH2* expression. Kaplan–Meier estimates of overall survival among non-NE-

enriched (NMF4) SCLC patients in the atezolizumab and placebo treatment groups of IMpower133 stratified by **(C)** *REST* and **(D)** *NOTCH2* expression. Vertical lines on survival graphs represent censored patients. P-values were calculated using a log-rank test. P-values were unadjusted and <0.05 were considered significant.

Supplemental Figure 2

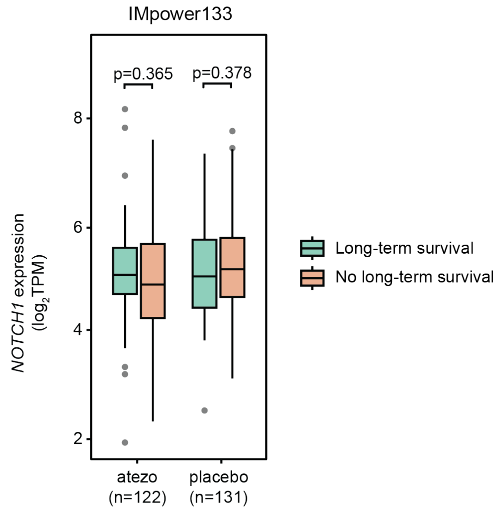

Supplemental Figure 2. *NOTCH1* expression among long-term survivors (≥18-month overall survival) and non-long-term survivors (<18 month overall survival) in atezolizumab and placebo treatment groups of the IMpower133 clinical trial. P-values were calculated using an unpaired two-tailed Student's t-test. P-values <0.05 were considered significant.

Supplemental Figure 3

A

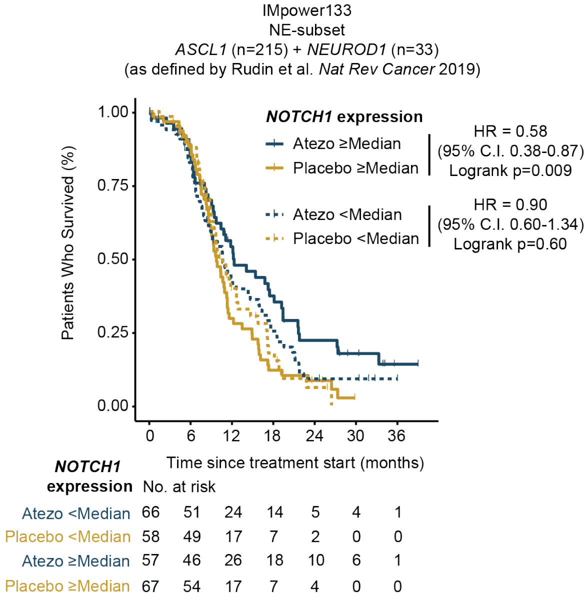

B

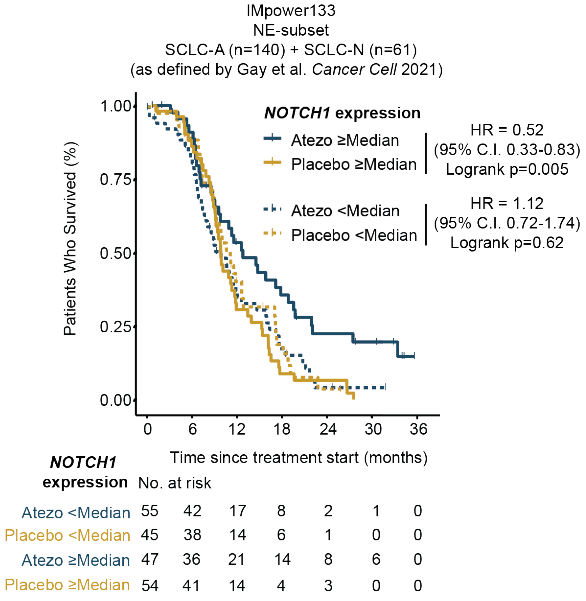

Supplemental Figure 3. High *NOTCH1* expression is significantly associated with longer overall survival with the addition of atezolizumab to first-line chemotherapy among *ASCL1* and *NEUROD1* subsets of extensive-stage SCLC patients in the IMpower133 clinical trial. Kaplan–Meier estimates of overall survival in the atezolizumab and placebo treatment groups of IMpower133 stratified by *NOTCH1* expression among NE subsets **(A)** *ASCL1* and *NEUROD1* as defined by Rudin et al. and **(B)** SCLC-A and SCLC-N as defined by Gay et al. Vertical lines on survival graphs represent censored patients. P-values were calculated using a log-rank test. P-values were unadjusted and <0.05 were considered significant.

Supplemental Figure 4

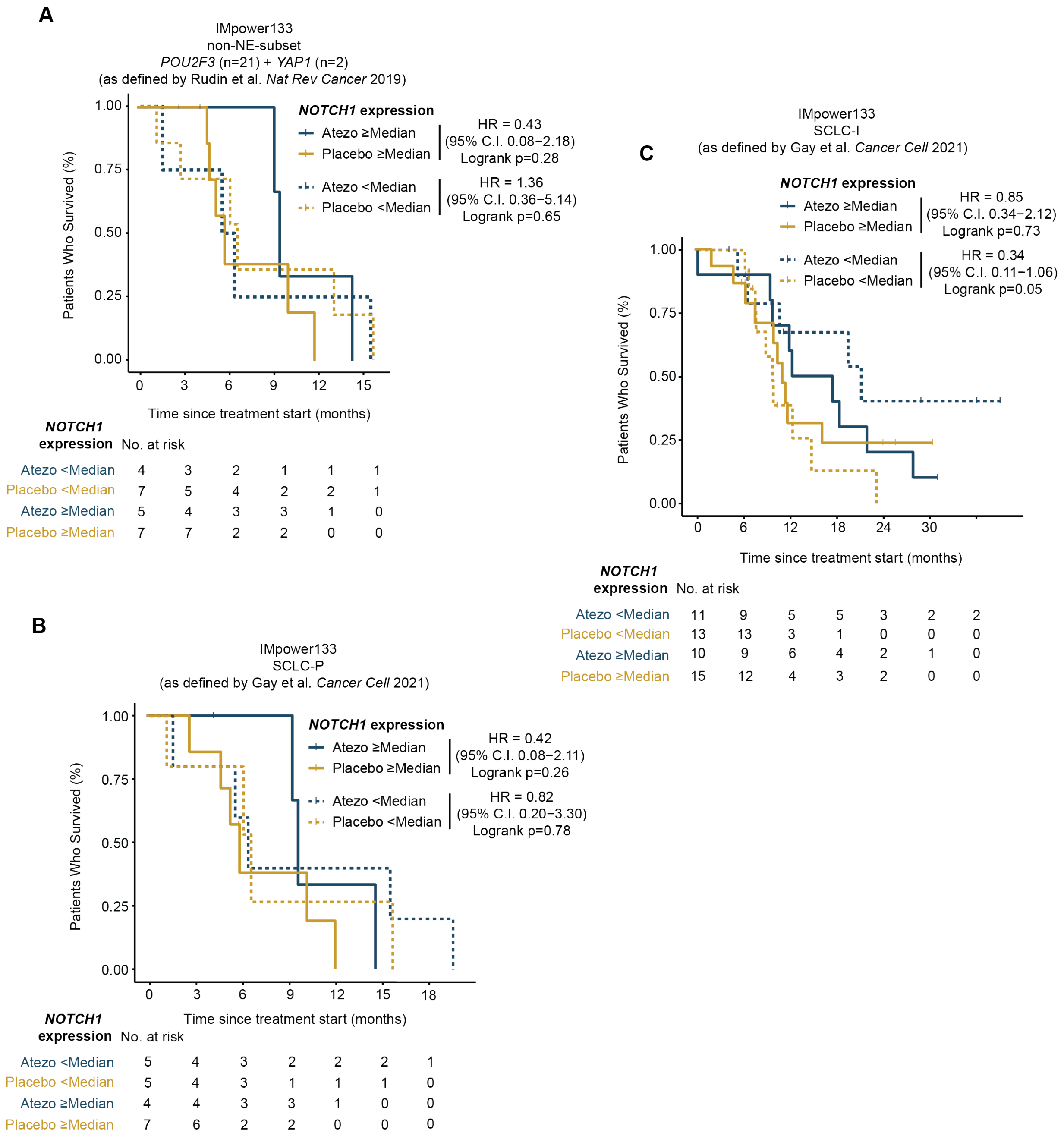

Supplemental Figure 4. High *NOTCH1* expression is not associated with longer overall survival with the addition of atezolizumab to first-line chemotherapy among non-NE and SCLC-I subsets of extensive-stage SCLC patients in the IMpower133 clinical trial. Kaplan–Meier estimates of overall survival in the atezolizumab and placebo treatment groups of IMpower133 stratified by *NOTCH1* expression among non-NE subsets **(A)** *POU2F3* and *YAP1* as defined by Rudin et al. and **(B)** SCLC-P as defined by Gay et al. Kaplan–Meier estimates of overall survival in the atezolizumab and placebo treatment groups of IMpower133 stratified by *NOTCH1* expression among the **(C)** SCLC-I subset as defined by Gay et al. Vertical lines on

survival graphs represent censored patients. P-values were calculated using a log-rank test. P-values were unadjusted and <0.05 were considered significant.

Supplemental Figure 5

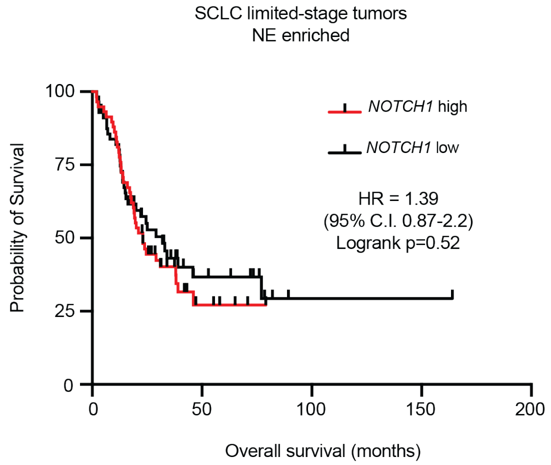

Supplemental Figure 5. Overall survival stratified by *NOTCH1* expression among NE-enriched (NMF1/2/3) patients in a combined cohort of limited-stage SCLC patients.

Supplemental Figure 6

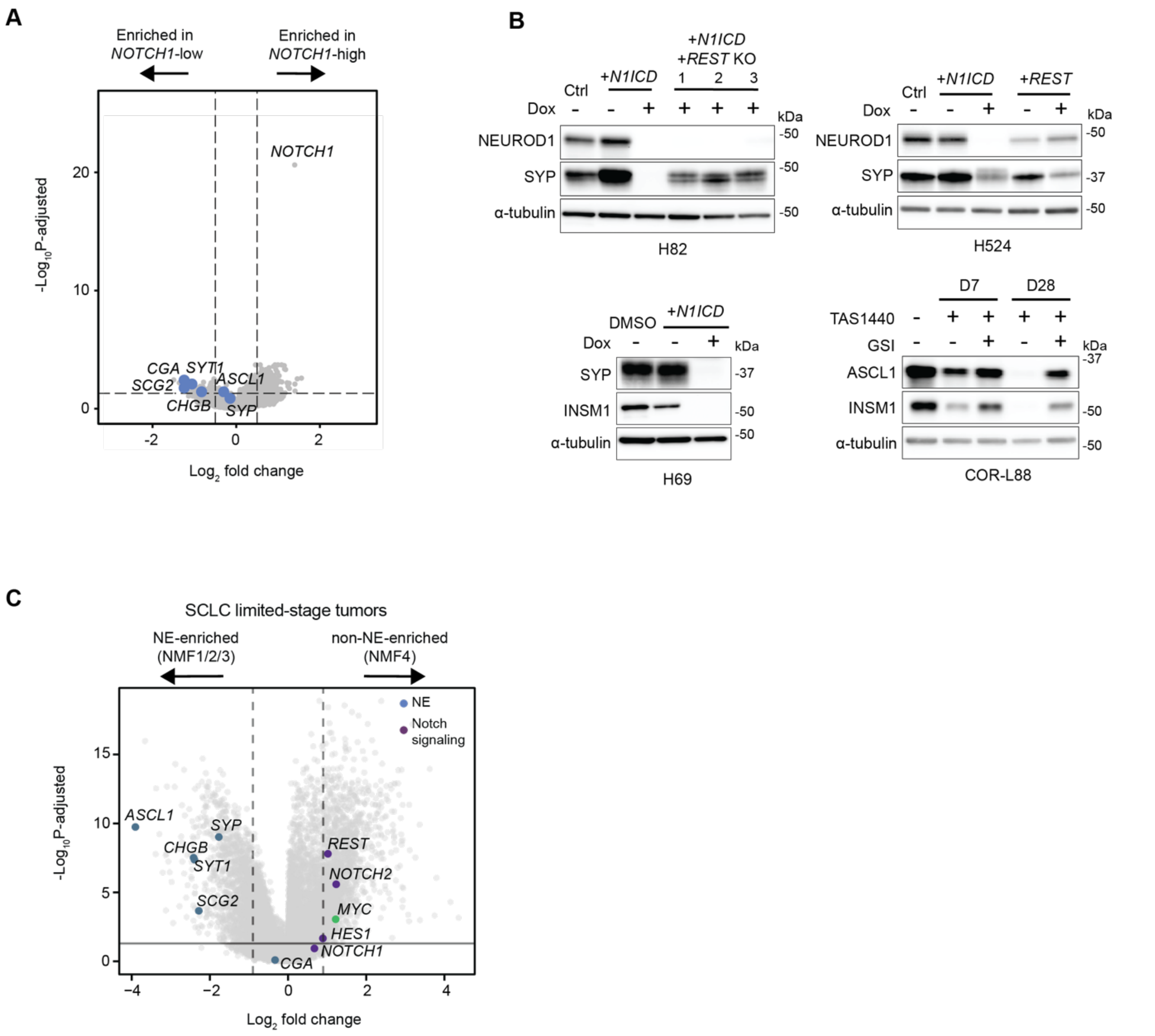

Supplemental Figure 6. NOTCH1 and NE to non-NE differentiation in IMpower133 and pre-clinical SCLC models. (A) Volcano plot showing differentially expressed NE genes between high and low *NOTCH1* expressing tumors in the NE-enriched (NMF1/2/3) subset of IMpower133. **(B)** Immunoblot analysis of indicated proteins in SCLC cell lines. The a-tubulin blots shown for H82 and COR-L88 cell lines in the panel also appear in Figure 4D and Figure 4J, respectively. **(C)** Volcano plot showing the comparison of differentially expressed Notch signaling, NE genes, and MYC in NE-enriched tumors and non-NE-enriched tumors in a combined cohort of limited-stage SCLC tumors. NE, neuroendocrine; Dox, doxycycline; KO, knockout.

Supplemental Figure 7

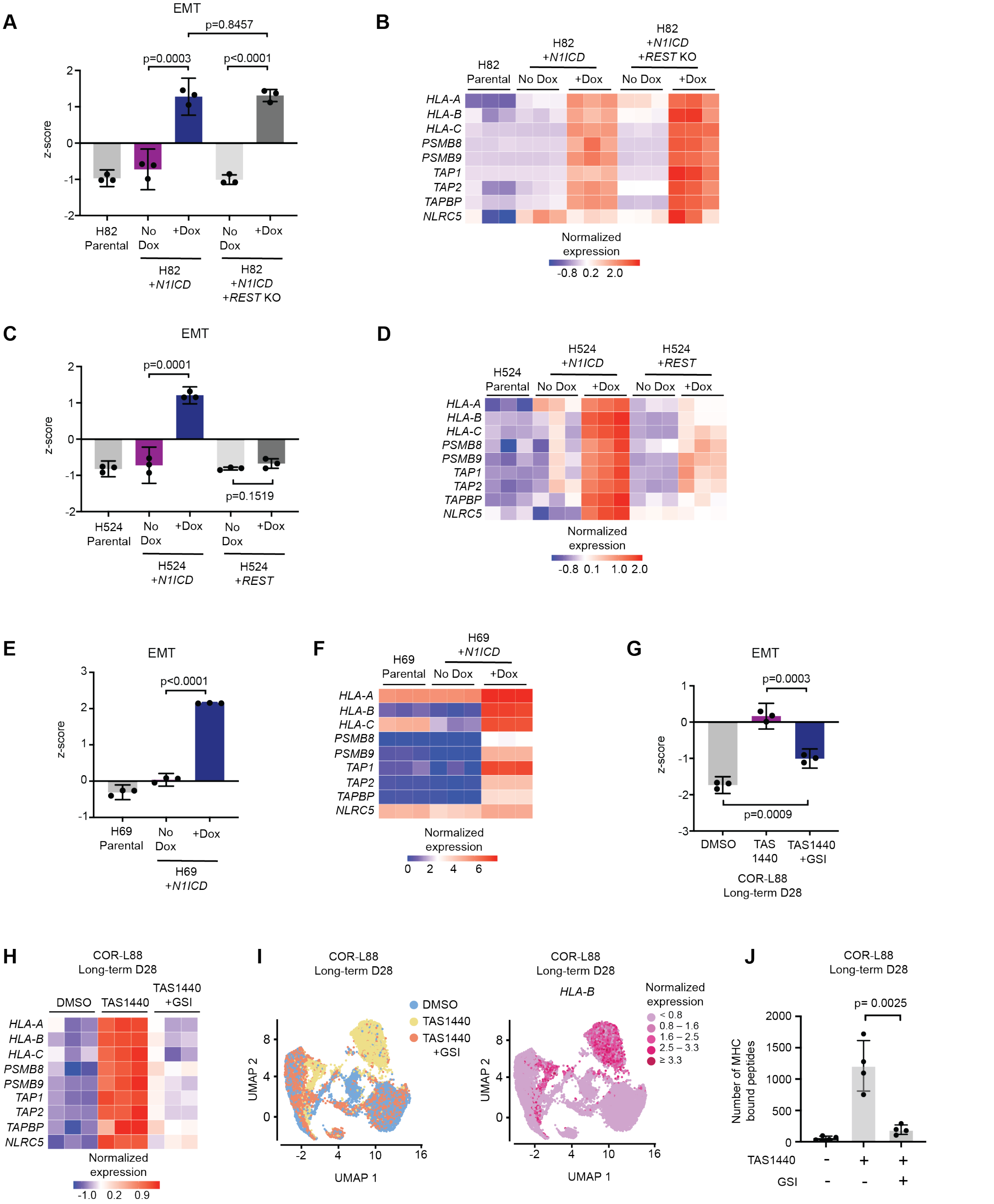

Supplemental Figure 7. Validation of the role of NOTCH1 in upregulating MHC Class I and antigen presentation in SCLC. (A) EMT signature (z-score) in long-term (56 days) *N1ICD* overexpressed H82 cells with or without *REST* KO. **(B)** Heatmap reporting normalized mRNA

expression of APM genes for long-term (56 days) *N1ICD* overexpression in H82 cells with or without *REST*. **(C)** EMT signature (z-score) in long-term (56 days) *N1ICD* and *REST* overexpression in H524 cells. **(D)** Heatmap reporting normalized mRNA expression of APM genes for long-term (56 days) *N1ICD* and *REST* overexpression in H524 cells. **(E)** EMT signature (z-score) in long-term (>56 days) overexpression of *N1ICD* in H69 cells. **(F)** Heatmap reporting normalized mRNA expression of APM genes for long-term (>56 days) overexpression of *N1ICD* in H69 cells. **(G-J)** Long-term (28 days) treatment of COR-L88 cells with DMSO, TAS1440, and TAS1440 plus GSI (BMS-708163, 2 μM) as indicated. **(G)** EMT signature (z-score). **(H)** Heatmap reporting normalized mRNA expression of APM genes. **(I)** Combined scRNA-seq UMAP plots and normalized *HLA-B* expression across sample clusters. **(J)** MHC Class I immunopeptidome mass spectrometry (n=3 independent experiments) analysis. P-values were calculated using an unpaired two-tailed Student's t-test. P-values <0.05 were considered significant. NOTCH1-ICD, NOTCH1 intracellular domain; APM, antigen presentation machinery; Dox, doxycycline; GSI, γ-secretase inhibitor; KO, knockout; EMT, epithelial-mesenchymal transition.

Supplemental Figure 8

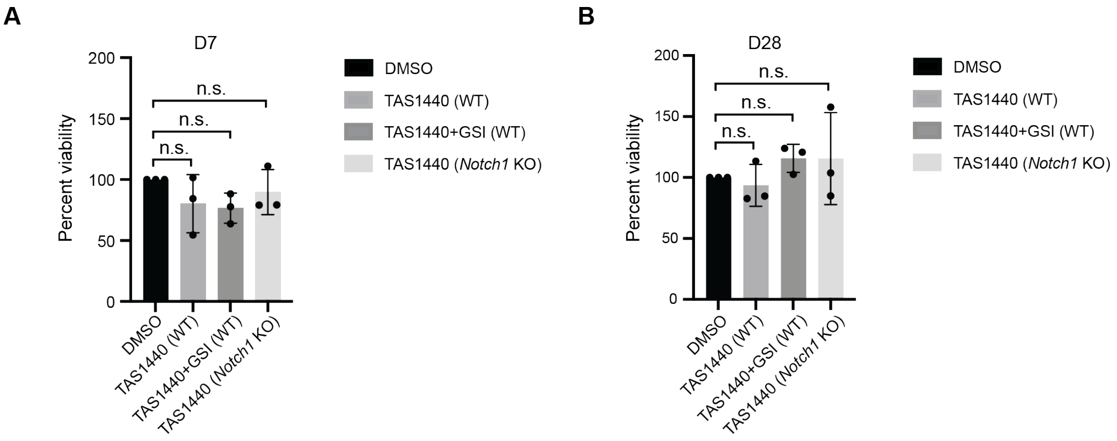

Supplemental Figure 8. Notch activation does not affect the viability of KP1 SCLC syngeneic mouse cells. KP1 cells growth at day 7 **(A)** and day 28 **(B)** with DMSO and TAS1440 (100 nM) treatment with or without GSI (DBZ, 10 μM). Data representative of n=3 independent experiments and data are represented as mean ± SD. P-values were calculated using an unpaired two-tailed Student's t-test. P-values <0.05 were considered significant.

Supplemental Figure 9

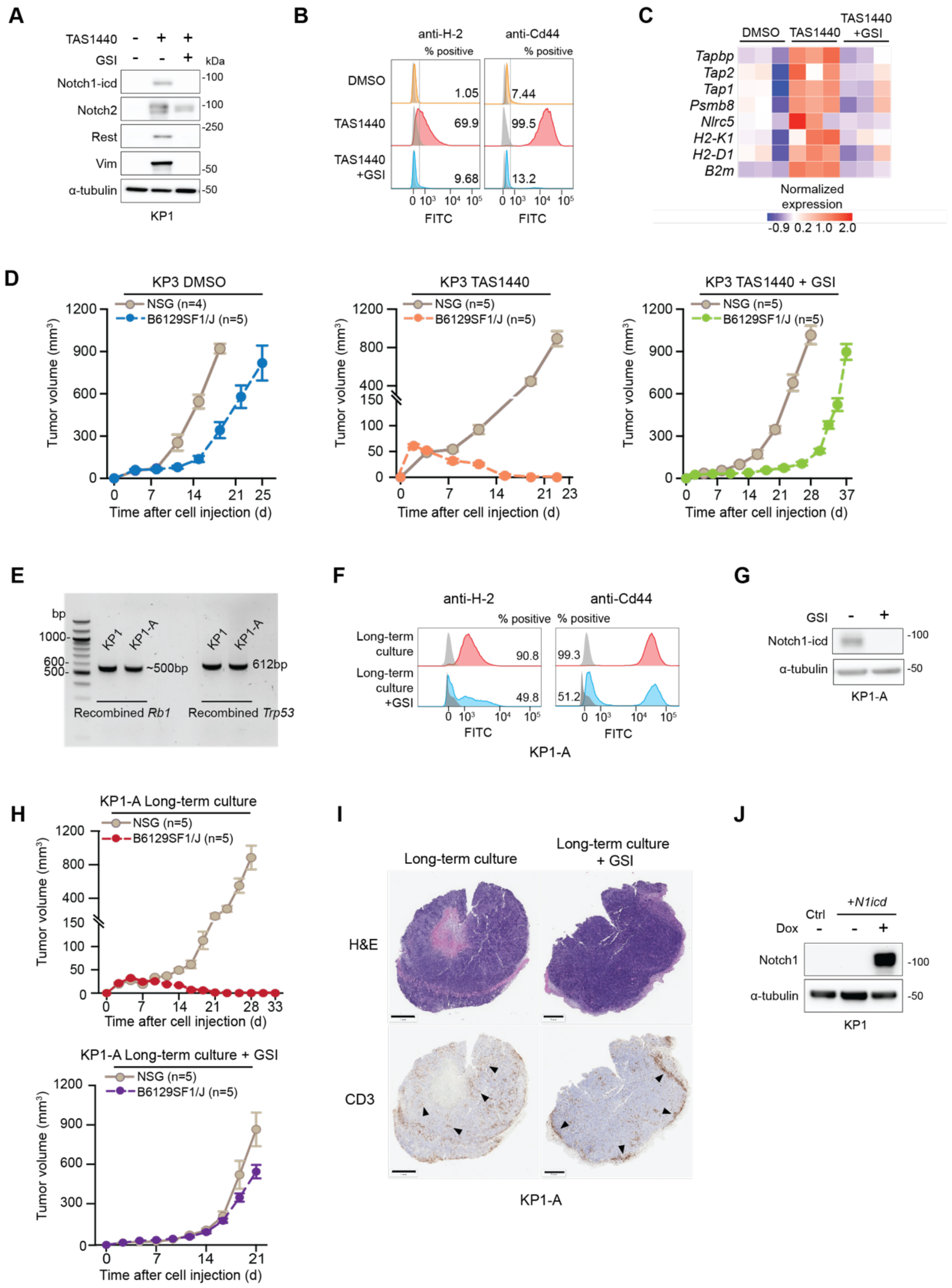

Supplemental Figure 9. Active Notch signaling is required to reverse immune suppression in SCLC. (A-C) Mouse KP1 cells treated long-term (>28 days) *ex vivo* with DMSO, TAS1440, and TAS1440 plus GSI (DBZ, 10 μM) **(A)** Immunoblot analysis of indicated proteins. **(B)** Flow

cytometry histograms of indicated markers. Data representative of n=3 independent experiments. **(C)** Heatmap reporting normalized mRNA expression of APM genes. **(D)** Mouse KP3 cells treated long-term (>28 days) *ex vivo* with DMSO, TAS1440, and TAS1440 plus GSI (DBZ, 10 μM) and were inoculated subcutaneously into the mice. Tumor growth curves of KP3 allografts in B6129SF1/J immunocompetent and NSG immunocompromised mice. **(E)** Validation of Cre-recombinase deletion of *Rb1* and *Trp53* in KP1 and KP1-A (KP1 adherent) SCLC cells. **(F-I)** Mouse KP1-A cells cultured long-term (>28 days) *ex vivo* with and without GSI (DBZ, 20 μM). **(F)** Flow cytometry histograms of indicated markers. **(G)** Immunoblot analysis of indicated proteins. **(H)** Tumor growth curves of KP1-A allografts in B6129SF1/J immunocompetent and NSG immunocompromised mice (data representative of n=2 independent experiments). **(I)** H&E (upper panel) and CD3+ T cell immunohistochemistry (lower panel) of KP1-A allograft tumors. Arrowheads point to T cell clusters. Scale bar represents 1 mm. **(J)** Immunoblot analysis of indicated proteins. For flow cytometry, shaded gray histograms represent unstained controls for each condition. Positive cells have an H2 or Cd44 signal higher than the referenced gray vertical line. Error bars on tumor growth curves from **(D, H)** represents S.E.M. Notch1-icd (N1icd), Notch1 intracellular domain; GSI, γ-secretase inhibitor.; Ctrl, control; Dox, doxycycline.

Supplemental Figure 10

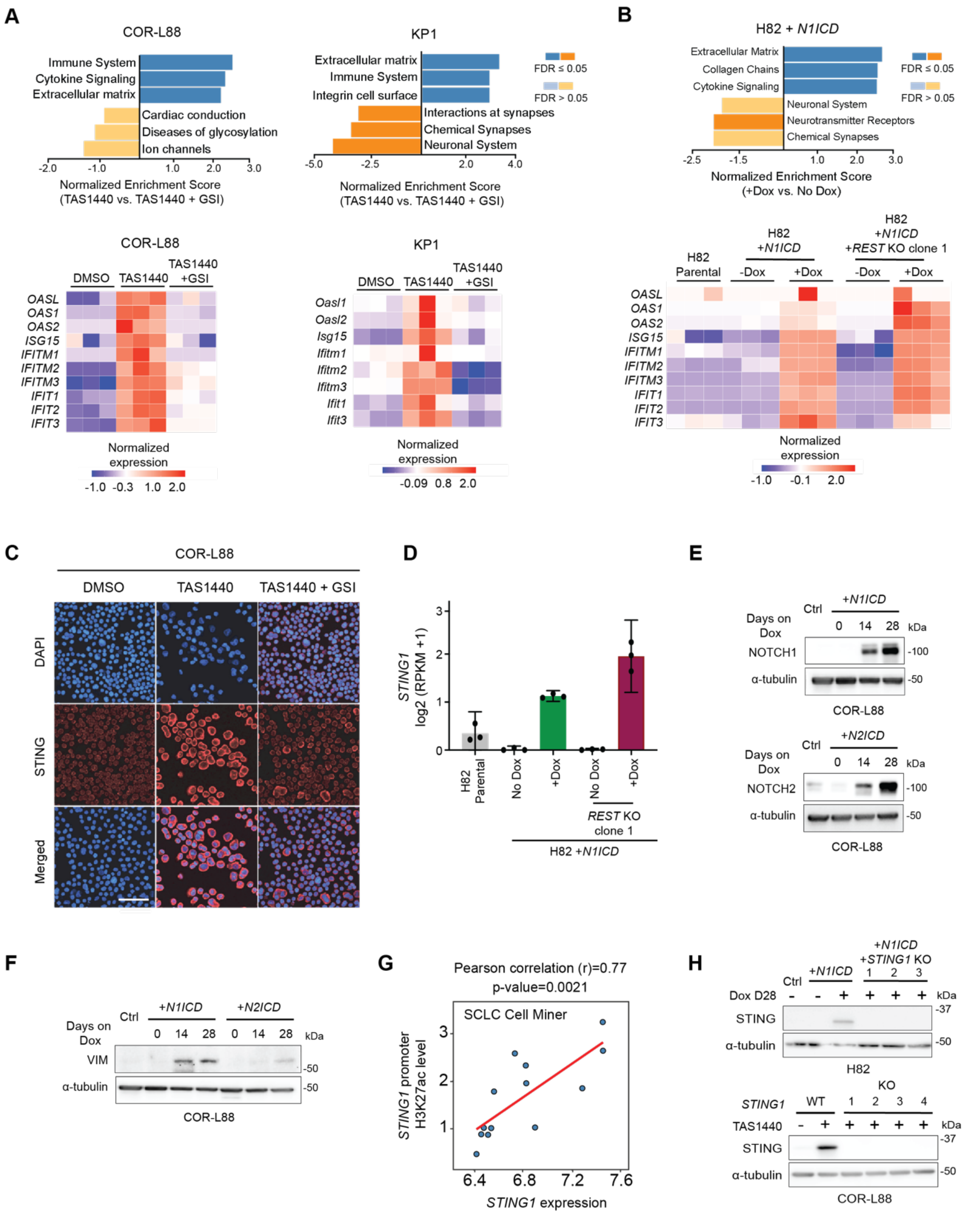

Supplemental Figure 10. NOTCH1 regulates SCLC intrinsic immunity through epigenetic modulation of STING. (A) Gene set enrichment analyses between TAS1440 and TAS1440 plus GSI treated COR-L88 and KP1 cells (upper panel) and heatmaps reporting normalized mRNA

expression of IFN signaling genes (lower panel). **(B)** Gene set enrichment analyses in H82 cells with and without *N1ICD* overexpression (upper panel) and heatmaps reporting normalized mRNA expression of IFN signaling genes (lower panel) in H82 parental cells, H82 cells with and without *N1ICD* overexpression, and H82 *REST* KO cells with and without *N1ICD* overexpression. **(C)** Representative immunofluorescence staining of STING in COR-L88 cells long-term (28 days) treated with DMSO, TAS1440, and TAS1440 plus GSI (data representative of n=2 independent experiments). Scale bar represents 50 µm. **(D)** mRNA *STING1* expression in H82 parental cells, H82 cells with and without *N1ICD* overexpression, and H82 *REST* KO cells with and without *N1ICD* overexpression. **(E, F)** Immunoblot analysis of indicated proteins in COR-L88 cells after NOTCH1-ICD or human NOTCH2-ICD overexpression**. (G)** SCLC CellMiner web interface correlation plot of *STING1* expression with *STING1* promoter level based on H3K27ac ChIP-seq across SCLC cell lines. (**H**) Immunoblot analysis of indicated proteins. Three single-cell *STING1* KO clones for H82 cells and four single-cell *STING1* KO clones for COR-L88 cells are shown. N1ICD, NOTCH1 intracellular domain; GSI, γ-secretase inhibitor.; Ctrl, control; Dox, doxycycline; KO, knockout.

Supplemental Figure 11

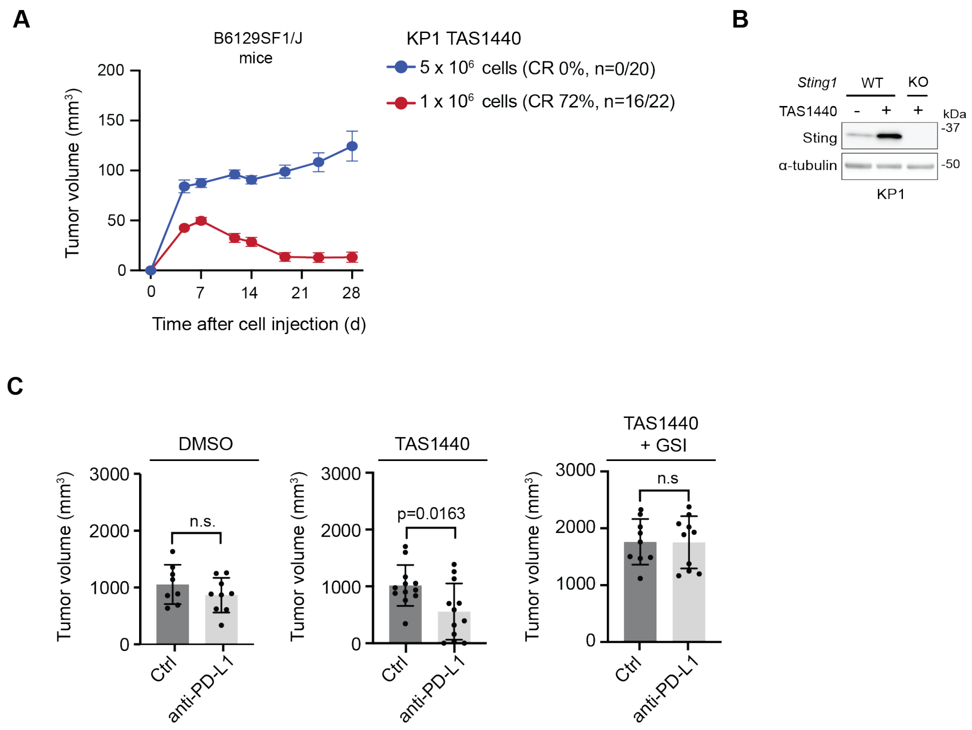

Supplemental Figure 11. Notch-driven SCLC tumors respond to anti-PD-L1 therapy. (A) Tumor growth curves of KP1 SCLC allografts treated *ex vivo* with TAS1440 with indicated cell numbers in B6129SF1 immunocompetent mice. Number of mice with complete tumor regressions within overall cohort size are shown (n=number of mice with complete tumor regressions/total number of mice). Error bars on growth curves represent S.E.M. combined from five ($1x10^6$ cells cohort) or two ($5x10^6$ cells cohort) independent experiments. **(B)** Immunoblot analysis of indicated proteins. **(C)** Endpoint tumor volumes of KP1 SCLC allografts treated ex vivo with DMSO, TAS1440, and TAS1440 plus GSI followed by in vivo treatment with anti-PD-L1 in B6129SF1 immunocompetent mice. Data show individual mice with mean ± S.D., analyzed by unpaired two-tailed Student's t-test. P-values <0.05 were considered significant. ns, no significance; GSI, γ-secretase inhibitor.

Supplemental Figure 12

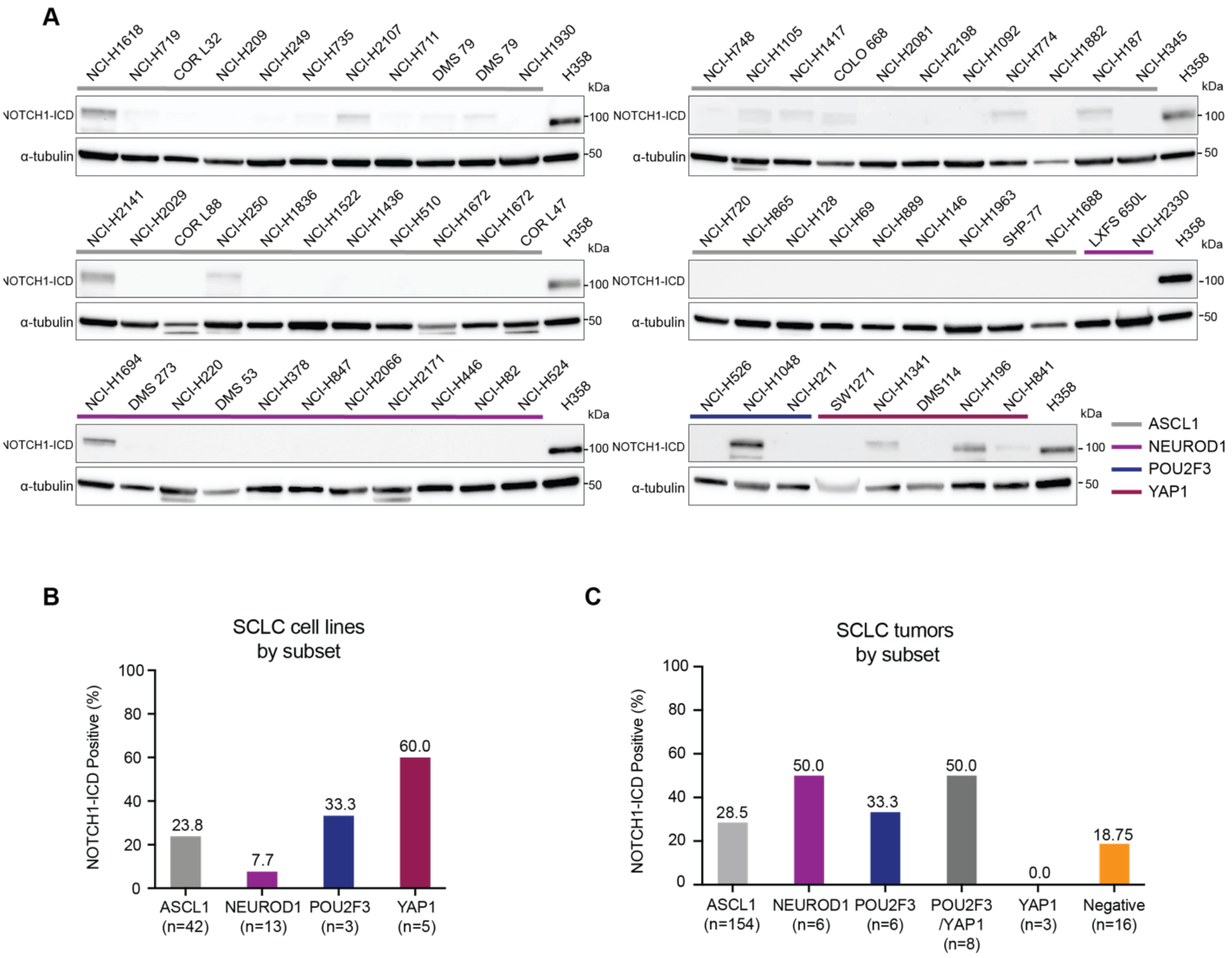

Supplemental Figure 12. NOTCH1-ICD expression across SCLC cell lines and human tumors. (A) Immunoblot analysis of NOTCH1-ICD across 63 SCLC cell lines stratified by subtype (ASCL1, NEUROD1, POU2F3, and YAP1). **(B)** Percentage of SCLC cell lines with NOTCH1-ICD expression stratified by subtype based on immunoblot analysis. **(C)** Percentage of SCLC human tumors with expression of NOTCH1-ICD by IHC stratified by subtype. NOTCH1-ICD, NOTCH1 intracellular domain.

Supplemental Figure 13

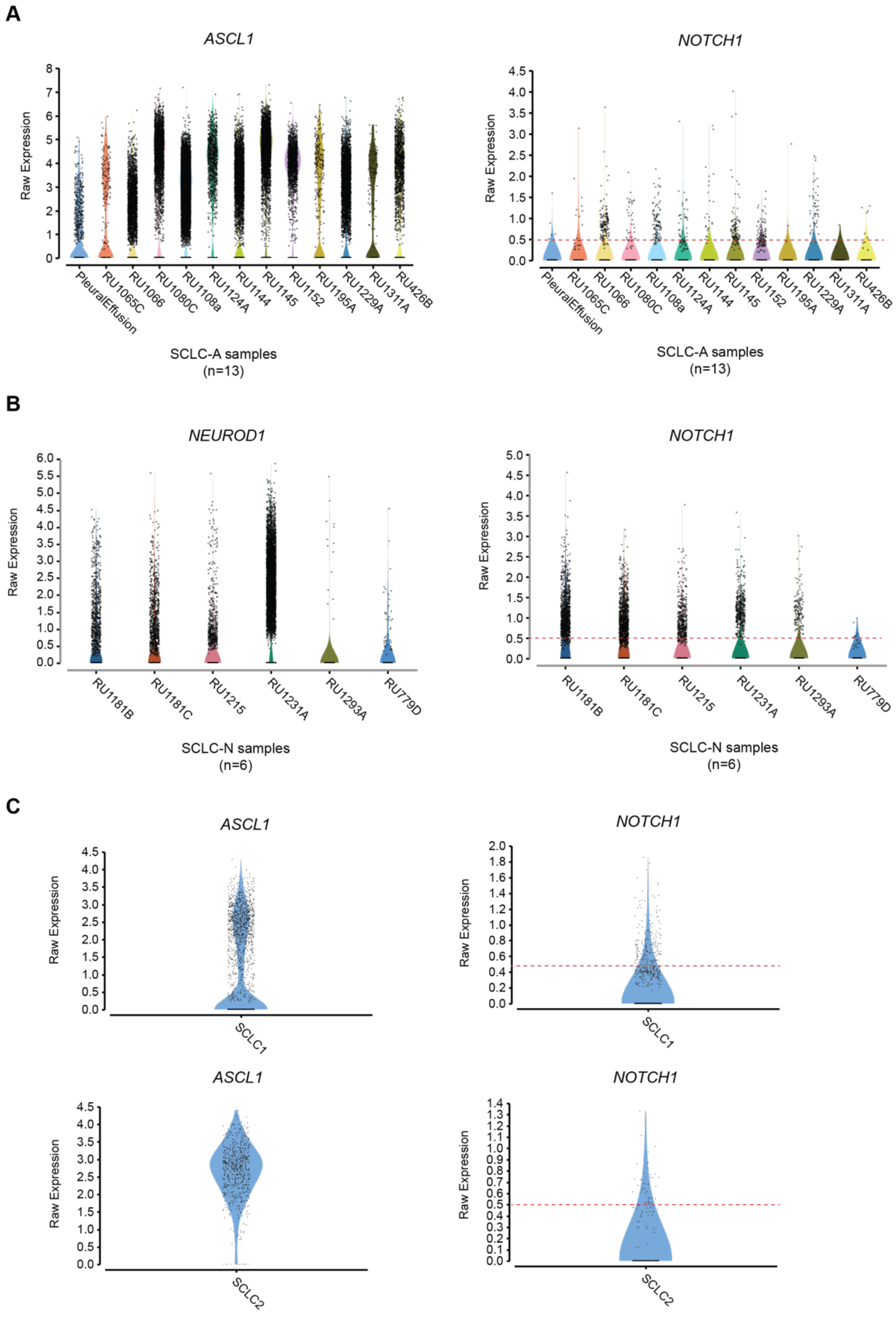

Supplemental Figure 13. Evidence of intra-tumor heterogeneity of *NOTCH1* expression by single-cell RNA sequencing. Single cell RNA-seq expression of *ASCL1, NEUROD1,* and

NOTCH1 in cohorts of **(A)** 13 *ASCL1*-enriched, **(B)** 6 *NEUROD1*-enriched, and **(C)** 2 *ASCL1*-enriched SCLC patient tumors.

Supplemental Figure 14

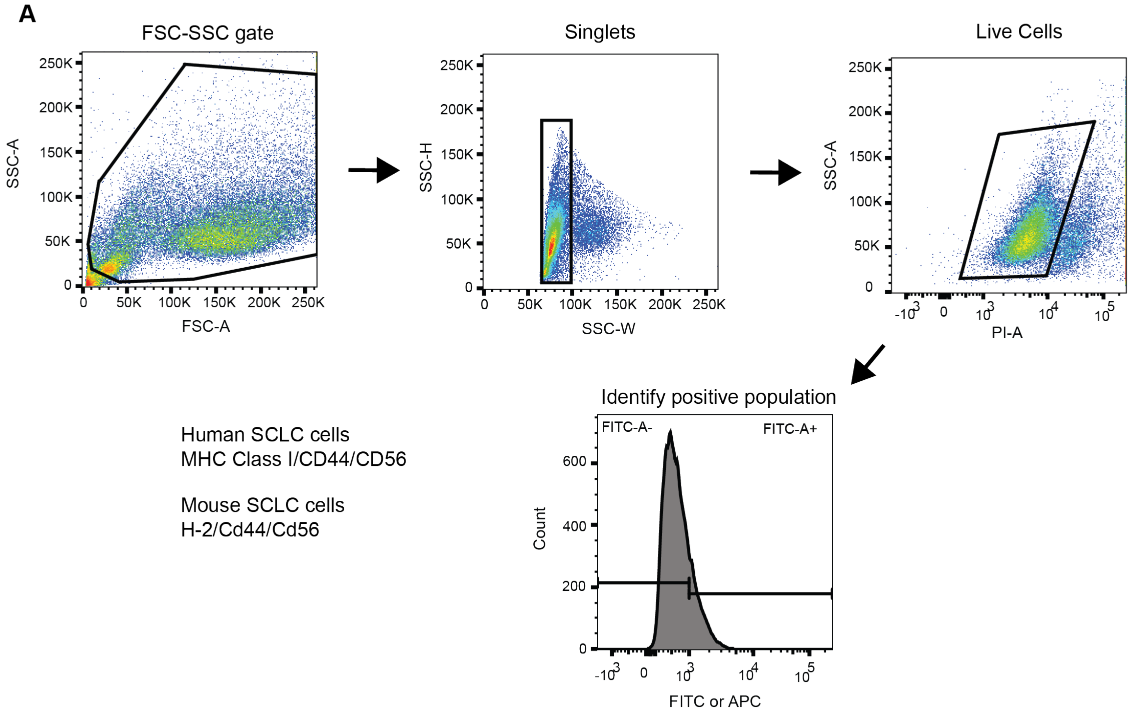

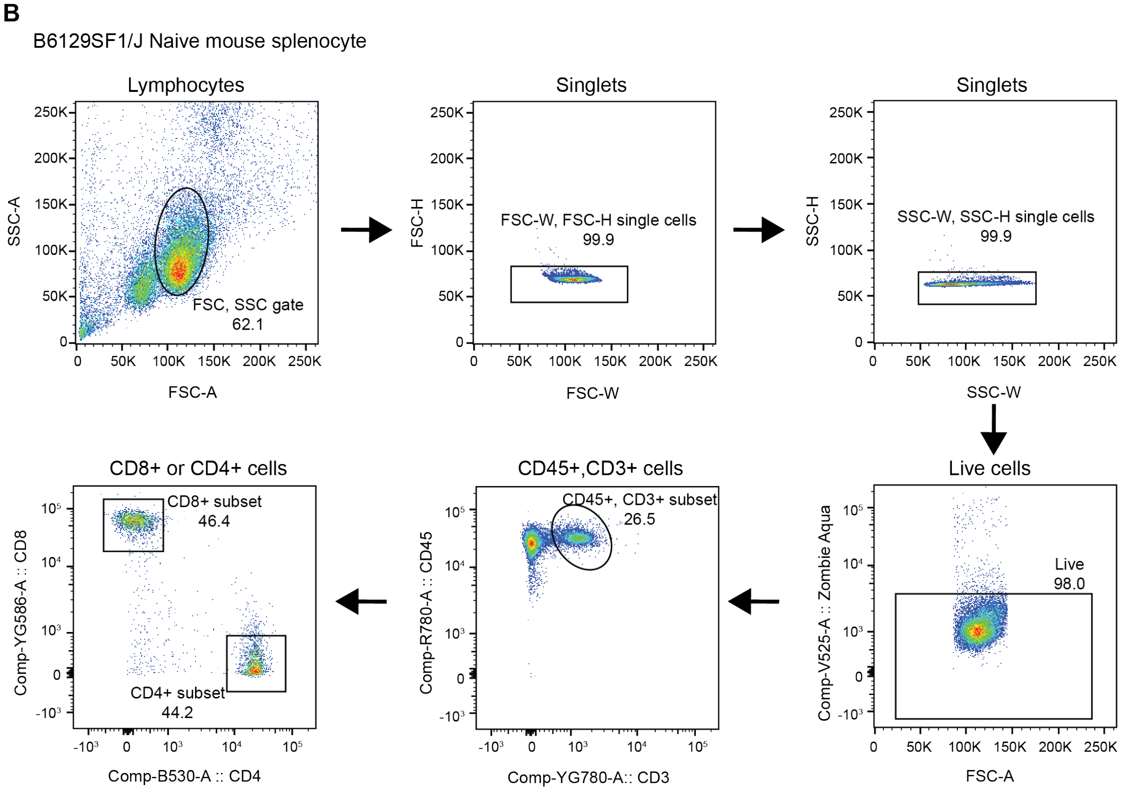

Supplemental Figure 14. Representative flow cytometric gating strategy used for in vitro and in vivo analyses. (A) Cells were gated based on FSC/SSC profiles and then further gated for singlets and PI-negative populations. For all in vitro models results, the *x*-axis represents

fluorescence intensity, and the *y*-axis represents normalized mode. **(B)** Splenocytes isolated from mice were analyzed by flow cytometry. Lymphocytes were gated based on FSC/SSC profiles and selected for singlets, $CD45^+CD3^+$ T cells, and then further gated for either $CD4^+$ and/or $CD8^+$ T cells. The gating strategy was used in vivo T cell depletion experiment.

Supplemental Methods

IMpower133 clinical trial analysis

Details on the design and analysis of RNA-seq data from the IMpower133 clinical trial have been previously reported (1). The IMpower133 NE (NMF1/2/3) and non-NE (NMF4) subsets were defined as in Nabet et al. (1). Tumors were classified as ASCL1, NEUROD1, POU2F3, or YAP1 based on the single highest gene expression as defined in Rudin et al.(2). Classification of IMpower133 tumors as SCLC-A, SCLC-N, SCLC-I, SCLC-P was based on a 1300 gene set as defined in Gay et al. (3). Long-term survivors (LTS) and non-LTS patients were defined as previously reported (4). Differential gene expression between NE (NMF1/2/3) and non-NE (NMF4) was performed using the R package limma with a focus on Notch signaling and neuroendocrine genes. GSEA was performed using previously published gene sets (5) and the QuSAGE package as previously reported (1). For Kaplan-Meier analyses, samples were dichotomized by the median within each subset as listed. An unbiased generalized random forest model (6, 7) was used as a method of nonparametric statistical estimation to identify which of the 32 genes of the Hallmark Notch signaling gene set may be predictive of overall survival with atezolizumab over placebo.

Cell lines

Human SCLC cell lines H82, H524, H446, and H69 were obtained from the American Type Culture Collection. Human SCLC cell line COR-L88 was a gift from Haobin Chen (Washington University). *Rb1/Trp53* mutant mouse SCLC KP1 and KP3 syngeneic cell lines were a gift from Julien Sage (Stanford University). H69, H82, H524, COR-L88, H446, KP1, and KP3 cell lines were cultured in RPMI-1640 (Corning #MT10040CM) supplemented with 10% fetal bovine serum (Gemini Bio #100-106) and 100 U mL^{-1} penicillin and 100 μg mL^{-1} streptomycin (Gibco #15140122). All cell lines were cultured in humidified incubators using 5% CO_2 at 37 °C. All cell

lines were regularly tested to be mycoplasma-negative (Lonza #LT07-318) and were authenticated by STR profiling (Laragen Inc.).

Lentiviral constructs and lentivirus production

Cells were virally transduced with human NOTCH1-ICD (pLIX-hN1ICD, Addgene plasmid #91897), human NOTCH2-ICD, human REST, or mouse Notch1-icd plasmids. The human NOTCH2-ICD, human REST, and mouse Notch1-icd plasmids were generated using the Gateway cloning method (Thermo Fisher Scientific, #11791020). Viral transduction was performed in the presence of polybrene (5-10 µg/mL, Sigma-Aldrich #TR-1003-G) and cells were centrifuged at 1200 x g for 4 h at 30 °C followed by removal of virus and polybrene. After 72 h, cells underwent selection with puromycin (4 µg/mL, Gibco #A1113803) or blasticidin (8 µg/mL, Gibco #A1113903) for 5 days. Cells were subsequently maintained on puromycin (1 µg/mL) or blasticidin (4 µg/mL). Doxycycline (1 µg/mL, Sigma-Aldrich #D9891-1G) was used induce each doxycycline-inducible vector. For CRISPR-Cas9 system, a single target sequences for CRISPR interference were designed using the sgRNA designer (https://portals.broadinstitute.org/gppx/crispick/public) and subcloned into the lentiCas9-Blast (Addgene #83480) and selected with blasticidin (4-8 µg/mL) for 5 days.

In vitro and ex vivo experiments

H82, H524, H446, H69, and KP1 cell lines were used for human or mouse NOTCH1-ICD overexpression, COR-L88 cell line was used for human NOTCH2-ICD overexpression, and H524 cell line was used for human *REST* overexpression. For all overexpression experiments, adherent cells were collected and analyzed after long-term culture (≥28 days) after doxycycline administration. Adherent cells were defined as cells that cannot be washed off the cell culture plate and require the use of trypsin to recover them. For LSD1 inhibition experiments, 100 nM of TAS1440 was used in COR-L88, KP1, and KP3 cell lines to induce Notch signaling and a GSI (2

μM BMS-708163, NCI Development Therapeutics Program #NSC 761217 for COR-L88 or 10 μM DBZ, Cayman chemical #14627 for KP1 and KP3) was used to block Notch signaling in the presence of TAS1440. For experiments with COR-L88, all cells were collected 28 days after treatment with DMSO, TAS1440, and TAS1440 plus GSI. KP1 and KP3 cells treated in long-term culture (>28 days) with DMSO, TAS1440, and TAS1440 plus GSI were used for all in vitro and ex vivo experiments.

Cell viability assays

Cells were counted on day 0 and plated in 6-well plates at 500,000 cells/mL in 2 mL of media. TAS1440 (100 nM), with or without GSI (DBZ, 10 μM), was added every 3–4 days with fresh media replacement, and cells were passaged at a 1:2 ratio. On the day of viability measurement, 150 μL of cells with media from each group was collected and transferred to a 96-well plate, followed by the addition of 50 μL of CellTiter-Glo reagent (Promega #G7573). Luminescence was then measured according to the manufacturer's instructions.

gDNA isolation and PCR

Cellular gDNA was extracted using a DNeasy Blood and Tissue kit (Qiagen #69504) and amplified by PCR with Taq DNA Polymerase (New England Biolabs #M0273S) using the gene-specific primers listed. Primers used to detect *Rb1* recombination were: 5' delta: 5′-CTCTAGATCCTCTCATTCTTCCC-3′ and 3' delta: 5′-GCAGGAGGCAAAAATCCACATAAC-3′ with ~500bp expected size. Primers used to detect *Trp53* recombination were: deltaA 5′-CACAAAAACAGGTTAAACCCAG-3′, deltaD 5′-GAAGACAGAAAAGGGGAGGG-3′ with 612bp expected size. PCR conditions for both primers were 3 min at 94 °C; 30 cycles of 30 sec at 94 °C, 30 sec at 58 °C, and 50 sec at 72 °C; and 5 min at 72 °C and held at 4 °C. PCR products were run on 1.5% agarose gels containing SYBR™ Safe DNA Gel Stain (ThermoFisher Scientific #S33102), and images were acquired using a ChemiDoc imager (Biorad).

Bulk RNA sequencing and analysis

RNA was extracted from cells using an AllPrep DNA/RNA extraction kit according to the manufacturer's protocol (Qiagen #80204). Samples were pooled and sequenced on NovaSeq 6000 SP using Illumina Stranded mRNA Prep and paired-end sequencing. Samples were trimmed for adapters and low-quality bases using Cutadapt before alignment with the reference genome (hg19) and the annotated transcripts using STAR. Gene expression quantification analysis was performed using STAR/RSEM tools. Read counts for each gene between samples were normalized using TMM method implemented in edgeR and then transformed to RPKM. Differential gene expression was conducted using limma voom. GSEA was subsequently performed, and enrichment scores were generated for Reactome pathways. Pathways with FDR less than 0.05 were considered significantly enriched. EMT was determined using single sample GSEA "Hallmark_Epithelial_Mesenchymal" values with z-scores calculated across all models.

For limited-stage SCLC patient RNA-seq data, we applied NMF (non-negative matrix factorization) using the top 5000 most variable genes from the IMpower133 SCLC dataset(1) on a combined cohort of 131 samples from two previously published studies (8, 9). NMF clustering identified k=4 as the most stable subset number using the cophenetic correlation. Subsequently, we stratified patients into NE (NMF1/2/3) and non-NE (NMF4) subsets and performed differential expression analysis between these two groups using "Limma R package" followed by generation of volcano plots.

Single-cell sequencing and analysis

Single cells were collected for the COR-L88 cell line by trypsinization. Subsequently, cell viability was assessed using a LunaFX7 automated fluorescent cell counter. Single-cell libraries were prepared using the 10x Genomics Chromium Next GEM Single Cell 5'v2 (Dual Index) kit, following the standard manufacturer's protocols, with a single capture lane allocated per sample. The target recovery was set at 8000 cells per lane. Cells were partitioned, followed by reverse

transcription of mRNA, and library preparation was carried out according to the user guide provided for the Chromium Connect system. Paired end sequencing was performed using NovaSeq 6000 SP 100-cycle. Base calling was performed using RTA 3.4.4, demultiplexing was performed using cellranger v7.1.0 (Bcl2fastq 2.20.0), and alignment was performed using cellranger v7.1.0 (STAR 2.7.2a). Sequenced reads were aligned to the 10x Genomics provided human reference sequence. H5 files from the CORL-88 experiment were processed, explored, and visualized using Cellenics community instance (https://scp.biomage.net/) hosted by Biomage (https://biomage.net/) using standard parameters. Cellenics was also used to re-analyze publicly available single-cell RNA seq data.

Chromatin Immunoprecipitation (ChIP) sequencing

ChIP was carried out following manufacturer's protocol (Active Motif #53008) for cross-linking, cell lysis, sonication, immunoprecipitation with H3K27ac antibody (Abcam #ab4729) until washing with magnetic beads. The beads were washed once with 0.8 mL Buffer 1, with 0.8 mL Buffer 2, and with 0.8 mL Tagmentation buffer (10 mM Tris pH 8.0, 5 mM $MgCl_2$, 0.1% TritonX-100). The beads were then resuspended in 20 µL of Tagmentation buffer, and 2 µL of Tagment DNA Enzyme from the Nextera DNA Sample Prep Kit (Illumina #FC-131-1024) and incubated at 37 °C for 30 min. Beads were then washed with 0.8 mL of Tagmentation buffer and once with 0.8 mL Buffer 2. The protocol was continued with reverse-crosslinking and DNA elution. The DNA was purified with ChIP DNA clean & concentrator (Zymo Research #D4014). Library was constructed following manufacturer's protocol (Illumina) and sequenced on NextSeq 500 using single-end sequencing. Raw FASTQ files were trimmed with the Trimmomatic software to remove low quality reads. Trimmed FASTQ files were subsequently checked for quality using FastQC. Trimmed reads with high quality were then aligned to the hg19 genome using the BWA aligner. Aligned (unsorted) reads were sorted using *samtools* followed by duplicate marking using picard-

tools. For viewing in the genome browser, scaled bigwig coverage tracks were generated for each alignment file using the scale option of the BAMscale tool.

ELISA

Conditioned media from cells at basal conditions was collected after 48 h and analyzed using Quantikine ELISA kit (R&D Systems) for human CXCL10 (#DIP100) or mouse IFNγ (#DY466-05).

Immunopeptidome

Cell pellets were lysed in 2 mL of HLA lysis buffer (50 mM Hepes, pH 7.4, 150 mM NaCl, 1 mM $CaCl_2$, 1 mM $MgCl_2$, 1% Triton X-100, 50 mM n-Octyl b-D-pyranoside and protease inhibitors) followed by pulse sonication to disintegrate the DNA. HLA peptides were eluted from HLA immune-complexes using mild acid-elution (0.5% TFA). The eluates were desalted using Sep-Pak Vac 1cc C18 Cartridges (Waters, Taunton, MA). The peptides were eluted from the C18 columns using a step gradient. The samples were then subjected to nanoflow liquid chromatography (ThermoFisher Scientific #Thermo Easy nLC 1000) coupled to high resolution tandem MS (Fusion, Thermo Scientific). MS scans were performed in the Orbitrap analyzer at a resolution of 60,000 with an ion accumulation target set at 1e6 and a max IT set at 50 ms over a mass range of 375-1575 m/z. A quadrupole isolation window of 1.6 m/z was used for MS/MS analysis. Acquired MS/MS spectra were searched against a human uniprot protein database using PEAKS Studio X plus software (Bioinformatics Software, Inc). A 1% false discovery rate (FDR) was calculated using a decoy database search approach.

Immunoblotting

Cells were lysed in RIPA buffer (Millipore #20-188) supplemented with protease inhibitor (Sigma-Aldrich #11836153001) and phosphatase inhibitors (Sigma-Aldrich #04906837001).

Protein concentration was measured using the Bio-Rad Detergent Compatible (DC) Protein Assay Kit (Bio-Rad #5000116). The antibodies used were cleaved NOTCH1 (CST #4147, clone D3B8; dilution 1:1000), NOTCH1 (CST #3608, clone D1E11; dilution 1:1000), NOTCH2 (CST #5732, clone D76A6; dilution 1:1000), REST (Sigma-Aldrich #07-579, polyclonal; dilution 1:1000), NEUROD1 (CST #4373, clone D35G2; dilution 1:1000), SYP (CST #36406, clone D8F6H; dilution 1:1000), STING (CST #13647, clone D2P2F; dilution 1:1000), p-STING (CST #50907, clone E9A9K; dilution 1:500 or 1:1000), TBK1 (CST #3504, clone D1B4; dilution 1:1000), p-TBK1 (CST #5483, clone D52C2; dilution 1:1000), IRF3 (CST #4302, clone D83B9; dilution 1:1000), p-IRF3 (CST #4947, clone 4D4G; dilution 1:1000), Vimentin (CST #5741, clone D21H3; dilution 1:1000), AXL (CST #8661, clone C89E7, R&D #AF854-SP; dilution 1:1000), ASCL1 (sc-374104, clone D7; dilution 1:750), INSM1 (sc-271408, clone A8; dilution 1:1000), and α-tubulin (Sigma-Aldrich #T9026, clone DM1A; dilution 1:15000). Secondary antibodies were from donkey anti-rabbit IgG-HRP (Cytiva #NA934), sheep anti-mouse IgG-HRP (Cytiva #NA931), and mouse anti-goat IgG-HRP (SCBT #sc-2354).

Immunofluorescence microscopy

Suspension cells were fixed using 2% paraformaldehyde (PFA) (Electron Microscopy Sciences #18713-S) and washed tree times with phosphate-buffered saline (PBS) (ThermoFisher Scientific #10010-072). Cells were then spotted onto slides by cytospin at 500 rpm for 4 min (Thermo Shandon Cytospin3). After cytospin, slides were quickly dried, placed in PBS for 15 min and subsequently permeabilized with cold 70% ethanol overnight. On the day of staining, slides were washed with PBS for 15 min and waterproof circles were drawn around the samples using a Pap Pen (Research Products International Corp. #195505.) to hold small volumes of solutions. Cells were blocked with 5% bovine serum albumin (BSA; Sigma-Aldrich #A9647-100G) in PBS containing 0.5% Tween-20 (Sigma-Aldrich #P9416-100ml) and 0.1% Triton X-100 (Sigma-Aldrich #T9284-100ml) (PBS-TT) for 1 h. After a 5 min wash with PBS, cells were incubated with primary

antibodies in 1% BSA in PBS-TT for 2 h at room temperature. The antibody used was STING (CST #13647, clone D2P2F; dilution 1:500). After 3 washes with PBS, 5 min each, the cells were incubated with secondary antibodies in 1% BSA in PBS-TT (Alexa 568–conjugated anti-mouse IgG, Invitrogen #A11004; dilution 1:500, Alexa 488–conjugated anti-rabbit IgG, Invitrogen #A11034; dilution 1:500) for 1 h at room temperature. After a 5 min wash with PBS, cells were counterstained with DAPI (4μg/mL in PBS) (ThermoFisher Scientific #D1306) for 10 min, washed once with PBS, mounted with DAPI-containing mounting medium (Vectashield, Vector Laboratories #H-1200) and sealed with nail polish. Fluorescence images were captured using a Zeiss LSM 780 confocal microscope.

Flow cytometry

Fresh tumor tissue was resected and stored in the MACS tissue storage solution (Miltenyi #130-100-008), dissociated with the mouse Tumor Dissociation Kit (Miltenyi #130-096-730) and mechanically separated using gentleMACS™ dissociators (Miltenyi #130-093-235) according to manufacturer's protocol. Multiparameter flow cytometric analysis was performed on dissociated cells. Cells were incubated with Zombie Aqua fixable viability kit (Biolegend #423101, #423102), Fc receptors were blocked prior to antibody staining using Fc receptor blocking agent, mouse (Miltenyi Biotec #130-092-575) and incubated for 20 min at 4 °C with antibodies for surface staining. For intracellular staining, cells were fixed and permeabilized using eBioscience™ Foxp3 /transcription factor staining buffer set (eBioscience #00-5523-00) according to the manufacturer's instructions, then stained with intracellular antibodies. The antibodies used were CD45 (Biolegend #103116, clone 30-F11), CD3 (Biolegend #100320, clone 145-2C11), CD4 (Biolegend #100406, clone GK1.5, Biolegend #116003, clone RM4-4), CD8 (Biolegend #100708, clone 53-6.7), CD69 (Biolegend #104539, clone H1.2F3), IFNγ (Biolegend #505810, clone XMG1.2), and Ki-67 (eBioscience #46-5698-82, clone SolA15). For detection of IFNγ, in vitro stimulation for 4 h using Cell Activation Cocktail with Brefeldin A (Biolegend #423303) was

performed. For flow cytometric analysis of cell surface markers in vitro, cells were resuspended in 50 uL PBS containing 1% BSA and stained with either human anti-HLA-A,B,C (Biolegend #311404, clone W6/32), anti-CD44 (Biolegend #338804, clone BJ18), or mouse anti-H-2K (Biolegend #125508, clone M1/42), anti-Cd44 (Biolegend #103022, clone IM7) for 30 min at 4 °C, washed by PBS containing 1% BSA. Samples were analyzed on LSR Fortessa (BD Biosciences) or MACSQuant Analyzer (Miltenyi Biotec) and data were analyzed using FlowJo software version 10.8.1. Flow cytometry gating strategies are shown in Supplemental Figure 14.

Functional *in vitro* T cell assay

Splenocytes from OT-I TCR transgenic mice were prepared by mechanical dissociation of the spleen tissue through a 40 μm nylon cell strainer (BD #21008-949). Red blood cells were depleted by incubating the splenocytes in RBC lysis reagent (Abcam #ab204733) according to the manufacturers' instruction. Splenocytes were cultured in RPMI-1640, 1X-GlutaMax, 2-Me (50 μM), supplemented with 10% fetal bovine serum and 100 U mL^{-1} penicillin and 100 μg mL^{-1} streptomycin in 24-well plates in the presence of 0.5 μg/mL OVA257-264 peptide (SIINFEKL; Sigma-Aldrich #S7951). After 3 days of culture, T cells were incubated with mouse recombinant IL-2 (100 U/mL) for 2 days, and then isolated with an EasySep™ mouse $CD8^+$ T cell Isolation Kit (Stemcell #19853) according to the manufacturer's instructions. KP1 luciferase expressing cells were ex vivo treated and were pulsed with OVA257-264 peptide for 4 h and then co-cultured with mouse $CD8^+$ OT-1 cells at a range of different effector to target (E:T) ratios. Assays were set up in quadruplicate for each condition. Viability of target cells was measured using a luminometer (Perkin Elmer Envision 2104 Plate-reader) after the addition of D-luciferin (ThermoFisher Scientific #L2916). For the crystal violet assay, target cells were plated in 12-well plates and pulsed with OVA257-264 peptide one day prior to co-culture. The following day, cells were co-cultured with CD8+ OT-I T cells at a range of E:T ratios. After 3 days of co-culture, wells were gently washed with PBS, fixed with methanol, and stained with 0.1% crystal violet solution. After

drying, plates were scanned using an Amersham Typhoon (Cytiva) and stained colony areas were quantified using ImageQuant TL software.

Mice

Seven-week-old B6129SF1/J (F1 hybrid strain #101043) female mice and OT-I transgenic male and female mice C57BL/6-Tg (TcraTcrb)1100Mjb/J (strain #003831) were obtained from Jackson Laboratories. Seven-week-old male and female NSG mice were obtained from the CCR Animal Research Program and used at roughly equal numbers. All mice were housed in accredited facilities on a 12 h light/dark cycle with free access to food and water under pathogen-free conditions.

In vivo studies

For the tumor growth experiment in NSG and B6129SF1/J mice, ex vivo treated KP1 or kP3 cells ($1x10^6$ cells for all groups), or *N1icd*-overexpressed KP1 cells were inoculated subcutaneously into the flank of NSG mice or B6129SF1/J mice in 100 µL of PBS with a 1:1 mixture of Matrigel (BD #356237). Mice were euthanized at a tumor burden endpoint of 1000 mm^3. The tumor size was measured three times a week using a digital caliper. Tumor volumes were calculated as length x $width^2$ x 0.5. For single-cell RNA sequencing, flow cytometry, IHC, and CODEX analyses, ex vivo treated KP1 cells ($5x10^6$ cells for all groups) were inoculated subcutaneously into the flank of B6129SF1/J mice and tumors were harvested 11 days after inoculation. For the *Notch1* KO tumor growth experiment, ex vivo treated KP1 WT or *Notch1* KO cells ($0.5x10^6$ cells for all groups) were inoculated subcutaneously into the flank of B6129SF1/J mice. For the STING agonist/ICB experiment, $0.5x10^6$ cells for KP1 DMSO and KP1 TAS1440 plus GSI groups and $5x10^6$ cells for the KP1 TAS1440 group were used. B6129SF1/J mice were then randomized at 100 mm^3 tumor volume and subjected to the following treatments: Ctrl, anti-PD-L1 (300 µg, Bioxcell #BE0101, clone 10F.9G2, intraperitoneal, once a week for 3 weeks),

MSA-2 (80 mg/kg b.w.; STING agonist obtained from NCI Development Therapeutics Program #NSC828583; oral gavage in 0.5% Hydroxypropyl methylcellulose solution, three times with 4 days between each dose), or a combination of anti-PD-L1 and MSA-2. Mice were euthanized at a tumor burden endpoint of 1500 mm^3 or 70 days post drugs dosing, whichever came first. For the rechallenge experiment, $5x10^6$ KP1 TAS1440 were inoculated subcutaneously into the B6129SF1/J mice with complete responses to anti-PD-L1 and MSA-2 combination treatment. For the T cell depletion experiment, anti-CD8a (250µg, Bioxcell #BE0117, clone YTS 169.4), anti-CD4 (250µg, Bioxcell #BE0003-1, clone GK1.5), or IgG2b isotype control (Bioxcell #BE0090) was injected intraperitoneally into B6129SF1/J mice 3 days before inoculation with KP1 TAS1440 cells ($1x10^6$ cells for all groups). Antibody injections for T cell depletion were continued every 3 days until the end of the experiment. T cell depletion was verified in mouse splenocytes by flow cytometry.

Immunohistochemistry

Human SCLC tumor microarrays (TMAs), LC703a, LC802c, and LC818c, were purchased from U.S. Biomax Inc. (Rockville, MD). TMA sections were stained by immunohistochemistry using the fully automated BOND IHC staining instruments (Leica Microsystem) according to the manufacturer's instructions. Briefly, sections were deparaffinized, pre-treated for heat-induced Ag retrieval (BOND ER2 protocol) in a pressure chamber (Pascal; Dako, Carpinteria, CA) with pH 9 EDTA buffer (Dako), and then incubated with hydrogen peroxide followed by the anti-cleaved NOTCH1 (CST #4147, clone D3B8; diluted 1:50) for 15 min at room temperature. Thereafter, sections were incubated for 8 min at RT using an Envision+Rb detection system (Dako). The substrate used for detection was 3.3′-diaminobenzidine (DAB) incubated for 10 min. Sections were then counterstained with hematoxylin for 5 min and covered in slides. Normal skin samples were included as positive controls.

Paraffin blocks for T cell IHC were sectioned at 5 μm and mounted onto positively charged slides. CD3 and CD8a staining was performed using a Leica Bond RX autostainer. Working dilutions for primary antibodies were CD3 (Bio-Rad #MCA1477, clone CD3-12; dilution 1:100) for 60 min and CD8a (eBioscience #14-0195-82, clone 4SM16; dilution 1:50) for 30 min. Antibody detection was accomplished using Biotinylated Rabbit anti-Rat (Vector Labs #BA-4001; dilution 1:100) followed by the Bond Polymer Refine Kit (Leica Biosystems #DS9800) with the post-primary reagent removed from Leica's default staining protocol. Antigen retrieval conditions for CD3 and CD8a were citrate buffer for 20 min at 100 °C. After staining slides were dehydrated and mounted for subsequent digital imaging.

Primary antibody was followed with Biotinylated Rabbit anti-Rat (Vector Labs #BA-4001; dilution 1:100) and ABC reagent (Vector Labs #PK-6100) per the manufacturer instructions. Staining was visualized with DAB, then counterstained with hematoxylin prior to dehydration and mounting for digital imaging.

Co-detection by indexing (CODEX) immunostaining

A multiplex immune fluorescence cytometric imaging technology for the simultaneously measurement of ~20-50 targets in a single tissue section, was carried out for immune cell profiling and spatial location for 3 samples (KP1 DMSO, KP1 TAS1440, and KP1 TAS1440 plus GSI) sectioned onto positively charged slides. OCT fresh frozen tissue samples were sectioned onto positively charged slides according to Akoya's recommendation. The slides were stained with CD3 antibody (Akoya Biosciences #4550109, clone 17A2; Akoya clone AKYP0035). Fixation, staining, and the assay was performed according to the manufacturer's recommendations and imaged on Phenocycler Fusion. The generated qptiff was loaded into HALO 2D digital pathology analysis software (IndicaLabs) for analysis, using Highplex FL module for single cell analysis and density heatmap and infiltration analysis.

References

1. Nabet BY, Hamidi H, Lee MC, Banchereau R, Morris S, Adler L, et al. Immune heterogeneity in small-cell lung cancer and vulnerability to immune checkpoint blockade. *Cancer cell.* 2024.
2. Rudin CM, Poirier JT, Byers LA, Dive C, Dowlati A, George J, et al. Molecular subtypes of small cell lung cancer: a synthesis of human and mouse model data. *Nature reviews Cancer.* 2019;19(5):289-97.
3. Gay CM, Stewart CA, Park EM, Diao L, Groves SM, Heeke S, et al. Patterns of transcription factor programs and immune pathway activation define four major subtypes of SCLC with distinct therapeutic vulnerabilities. *Cancer cell.* 2021;39(3):346-60.e7.
4. Liu SV, Mok TSK, Nabet BY, Mansfield AS, De Boer R, Losonczy G, et al. Clinical and molecular characterization of long-term survivors with extensive-stage small cell lung cancer treated with first-line atezolizumab plus carboplatin and etoposide. *Lung cancer (Amsterdam, Netherlands).* 2023;186:107418.
5. Bagaev A, Kotlov N, Nomie K, Svekolkin V, Gafurov A, Isaeva O, et al. Conserved pan-cancer microenvironment subtypes predict response to immunotherapy. *Cancer cell.* 2021;39(6):845-65 e7.
6. Feuerriegel S, Frauen D, Melnychuk V, Schweisthal J, Hess K, Curth A, et al. Causal machine learning for predicting treatment outcomes. *Nature medicine.* 2024;30(4):958-68.
7. Athey S, Tibshirani J, and Wager S. Generalized random forests. *The Annals of Statistics.* 2019;47(2):1148-78, 31.
8. George J, Lim JS, Jang SJ, Cun Y, Ozretic L, Kong G, et al. Comprehensive genomic profiles of small cell lung cancer. *Nature.* 2015;524(7563):47-53.
9. Liu Q, Zhang J, Guo C, Wang M, Wang C, Yan Y, et al. Proteogenomic characterization of small cell lung cancer identifies biological insights and subtype-specific therapeutic strategies. *Cell.* 2024;187(1):184-203 e28.
