# Supplementary material for: NOTCH1 reverses immune suppression in small cell lung cancer through reactivation of STING
Source: J Clin Invest. 2025 Jul 8;135(18):e185423. doi: 10.1172/JCI185423 (PMC12435836; doi:10.1172/JCI185423)

Full unedited image for Figure 4D

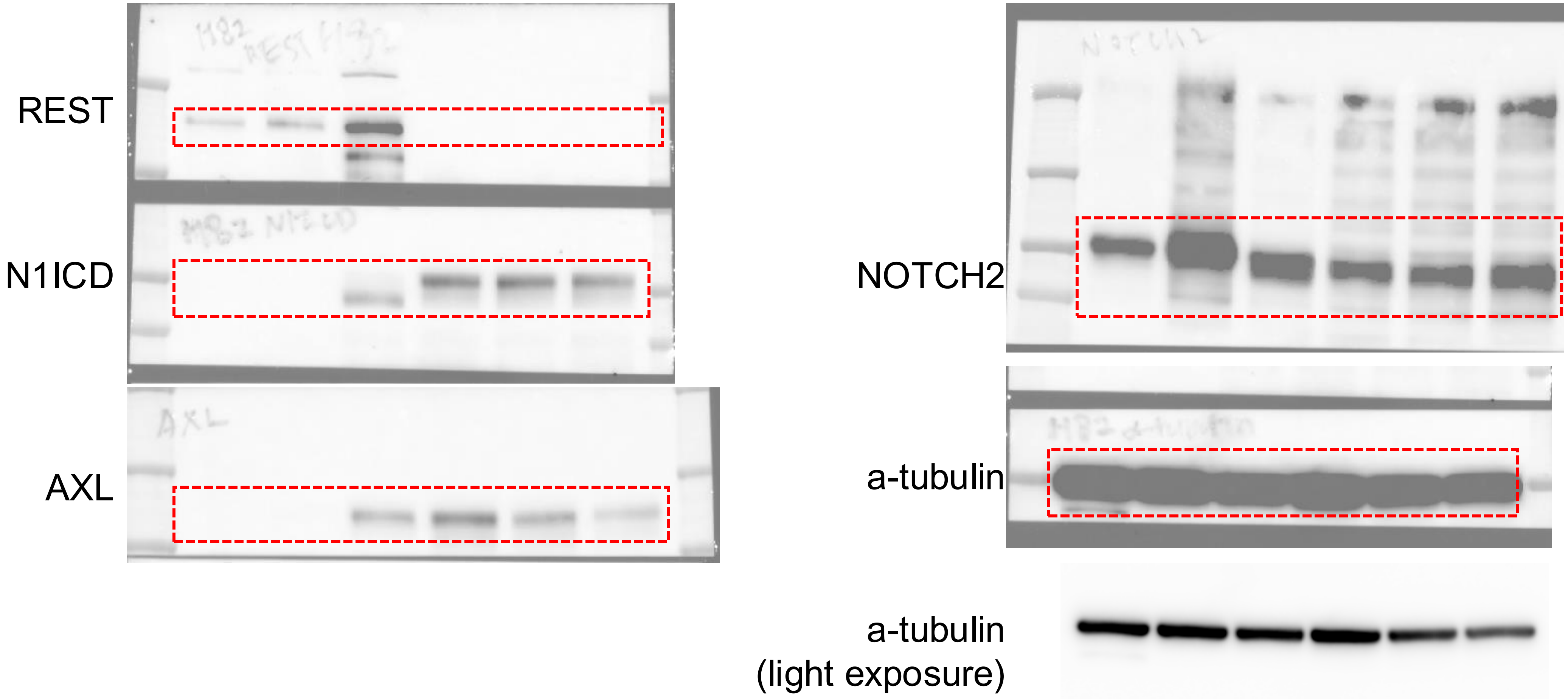

Full unedited image for Figure 4F

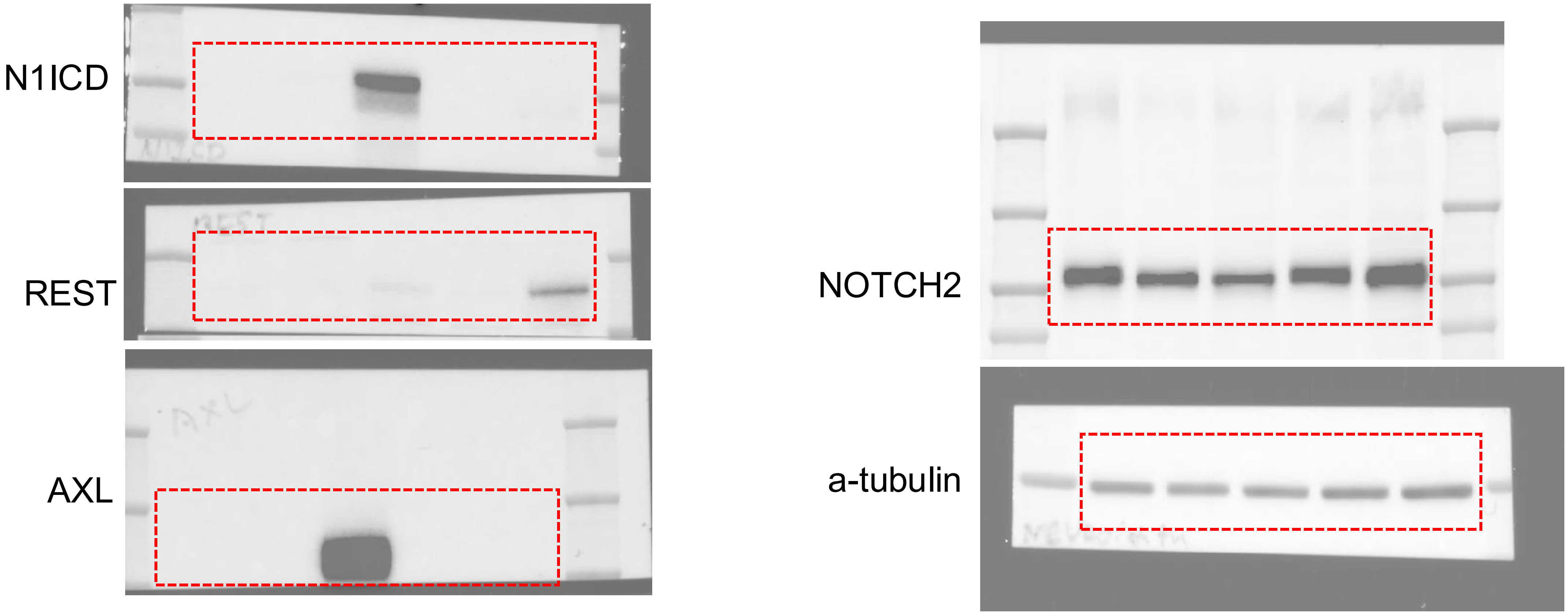

Full unedited image for Figure 4H

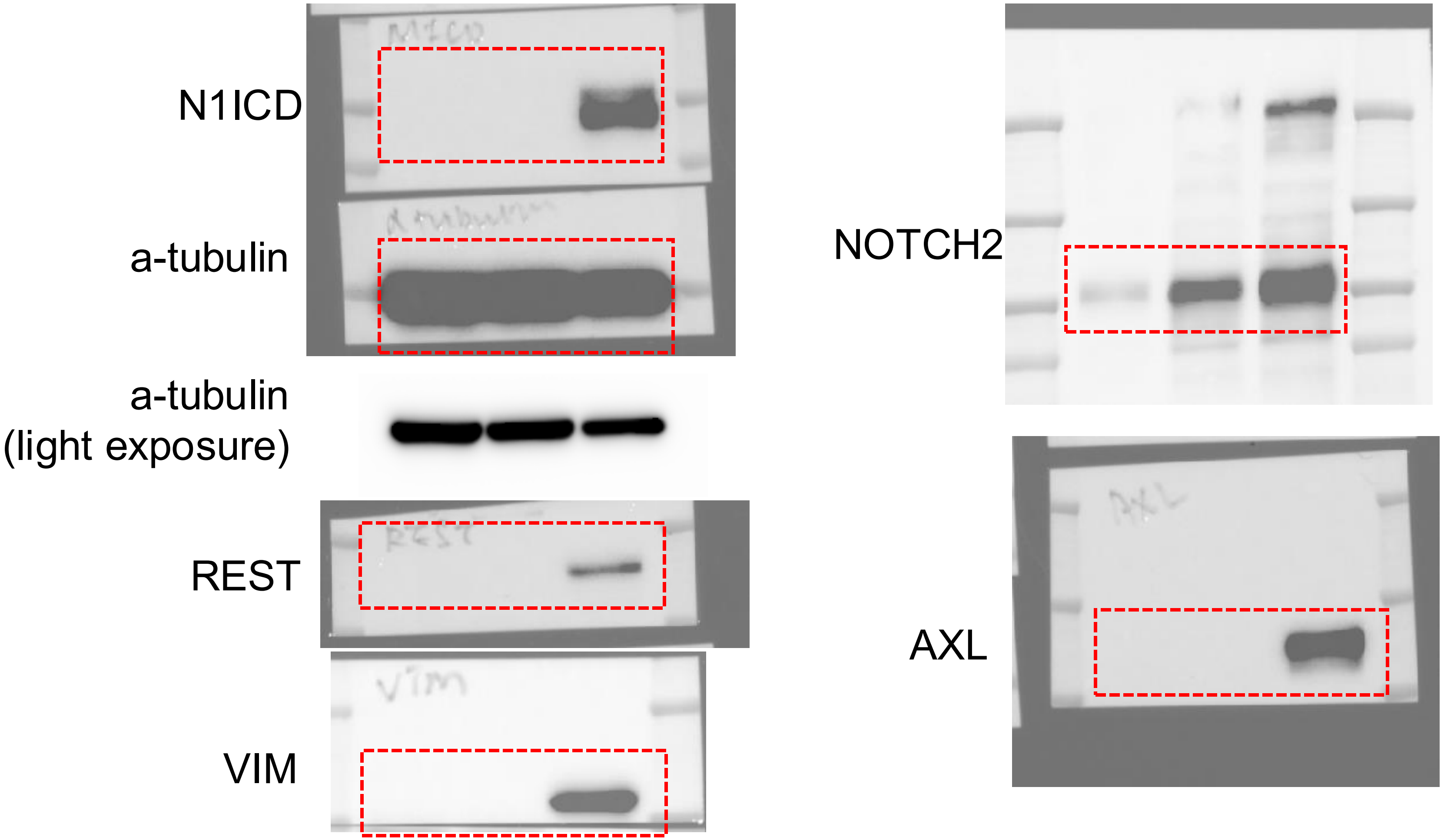

Full unedited image for Figure 4J

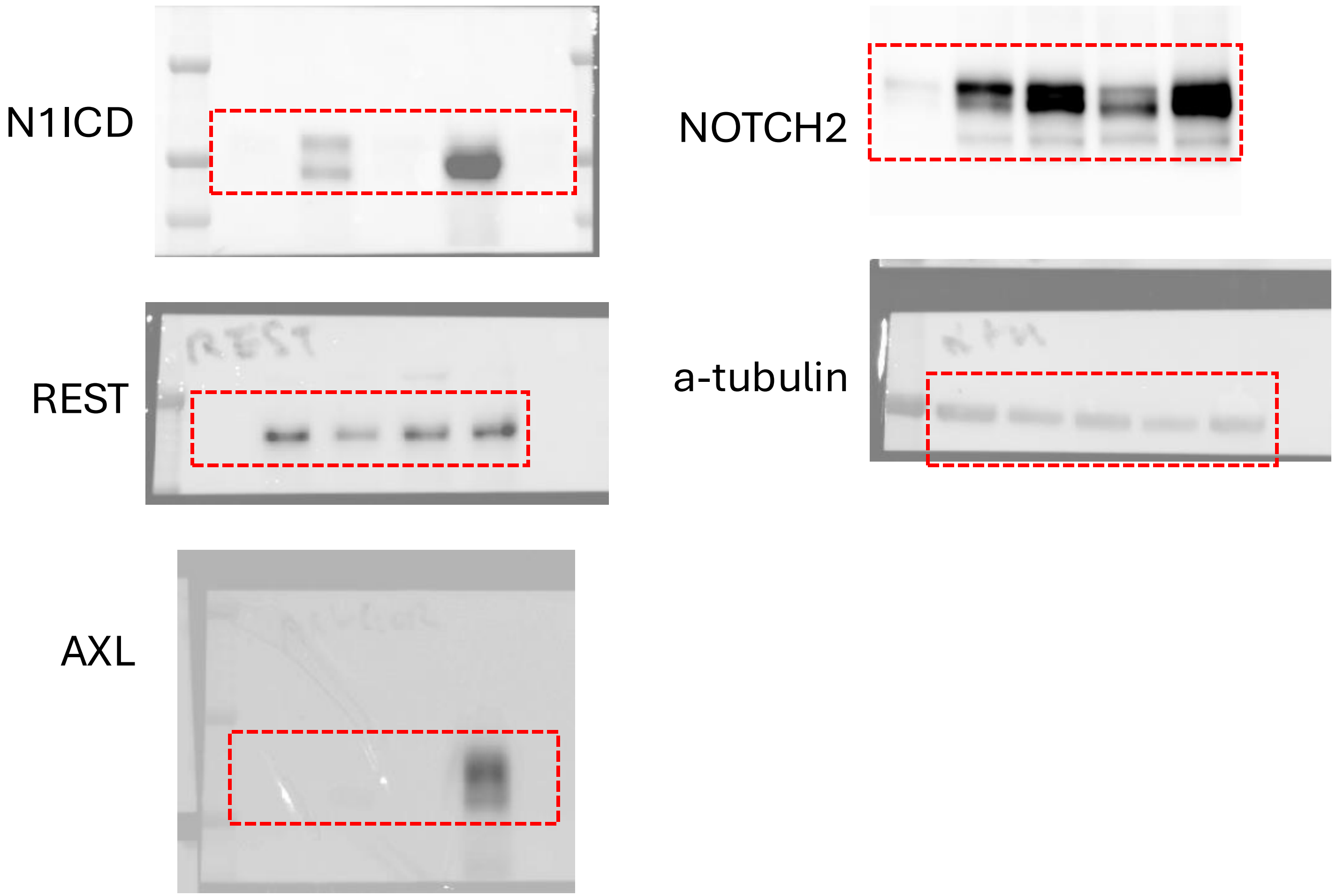

Full unedited image for Figure 4N

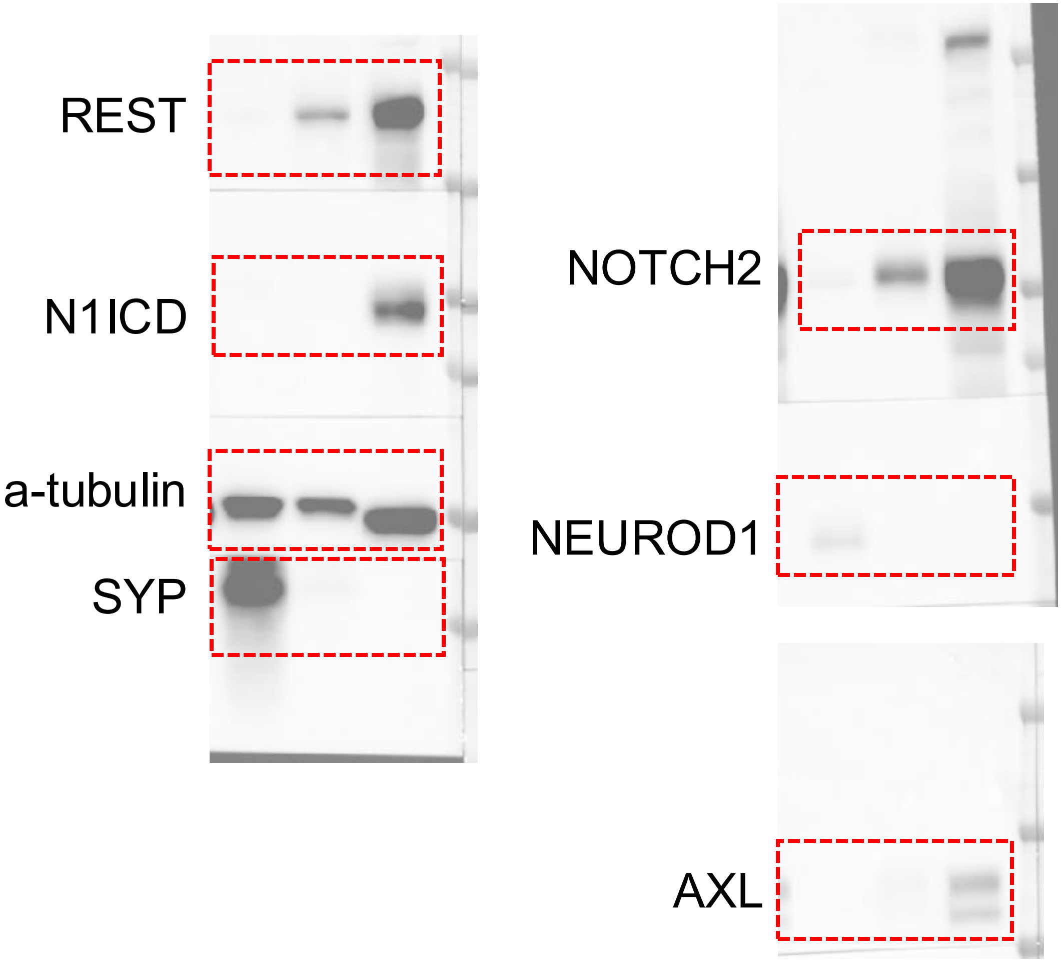

Full unedited image for Figure 6A

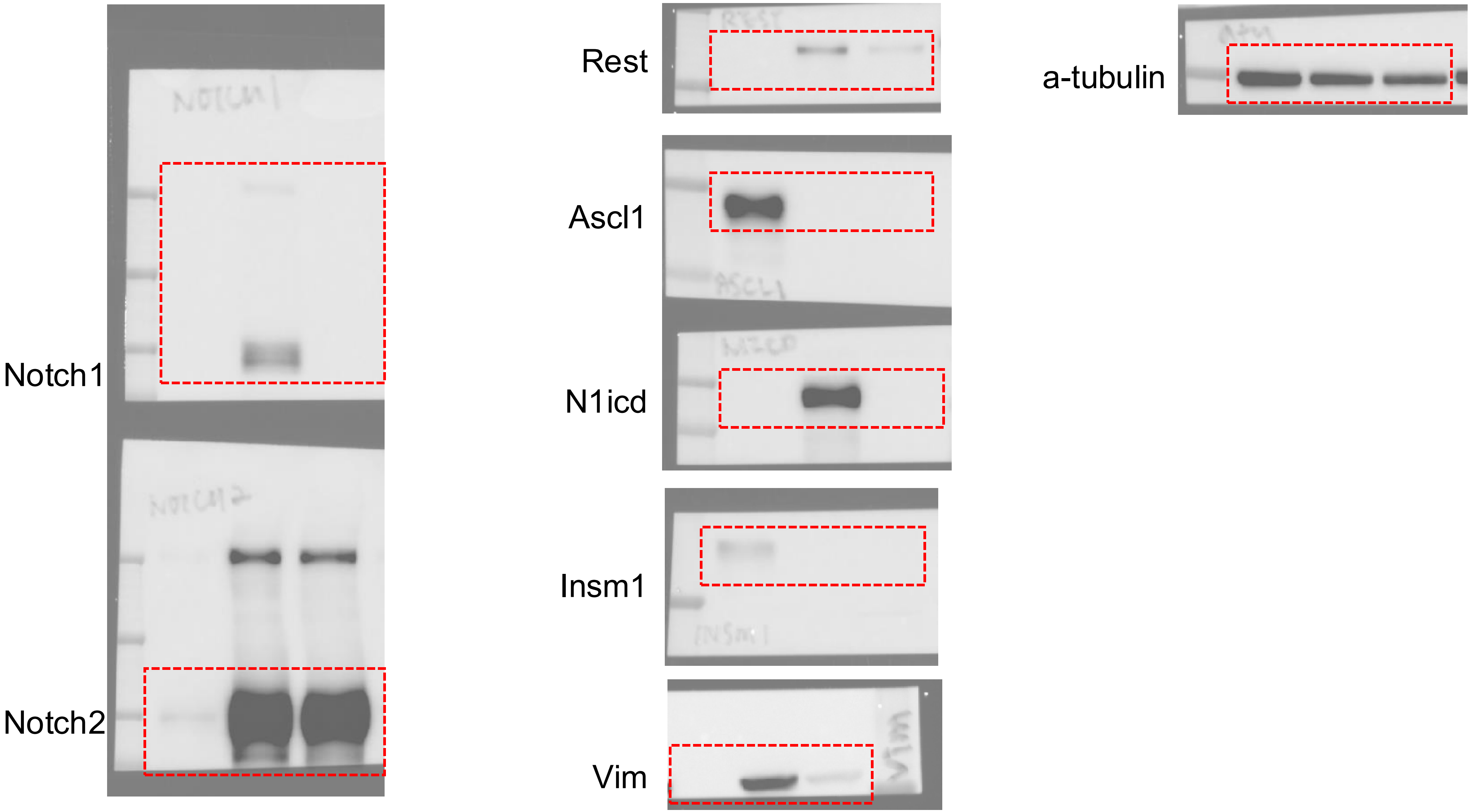

Full unedited image for Figure 7A, C, D, F

STING

a-tubulin

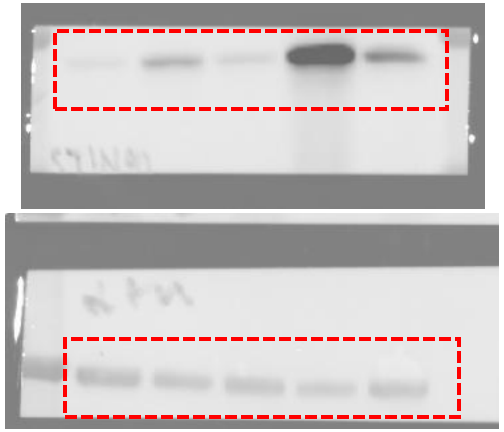

Figure 7A

STING

a-tubulin

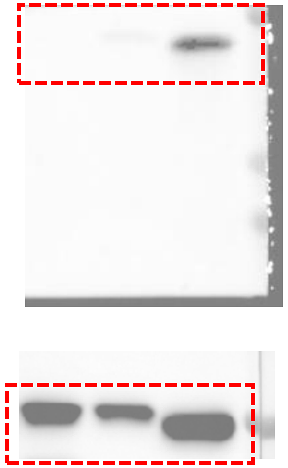

Figure 7C

STING

a-tubulin

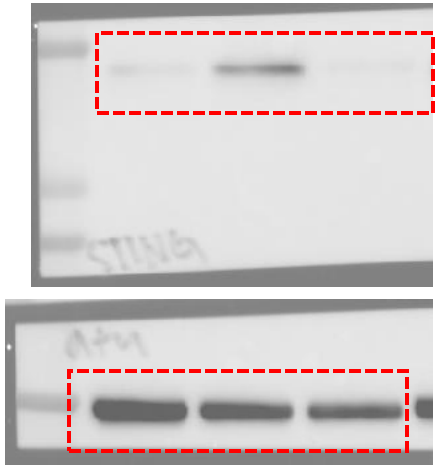

Figure 7D

STING

a-tubulin

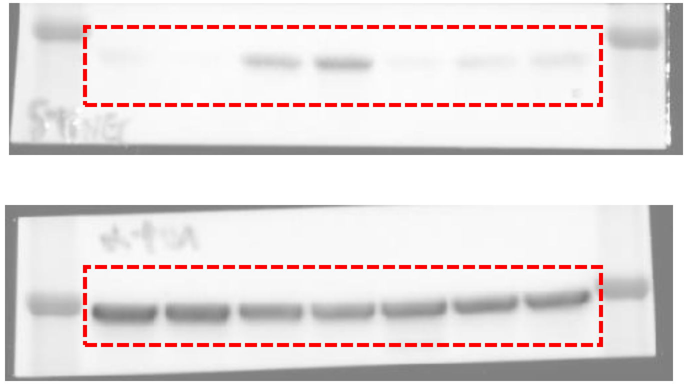

Figure 7F

Full unedited image for Figure 7J

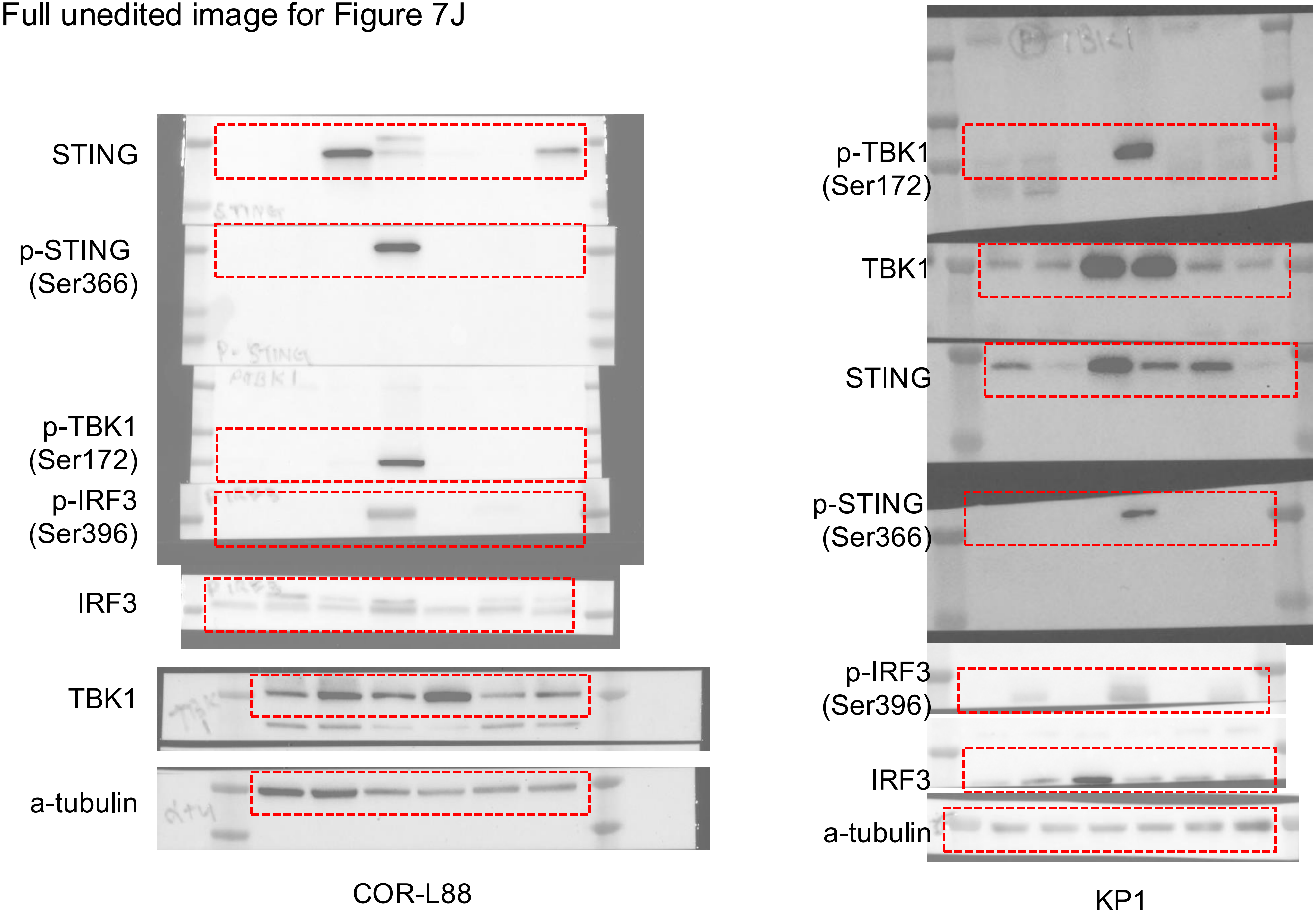

Full unedited image for Supplemental Figure 6B_1

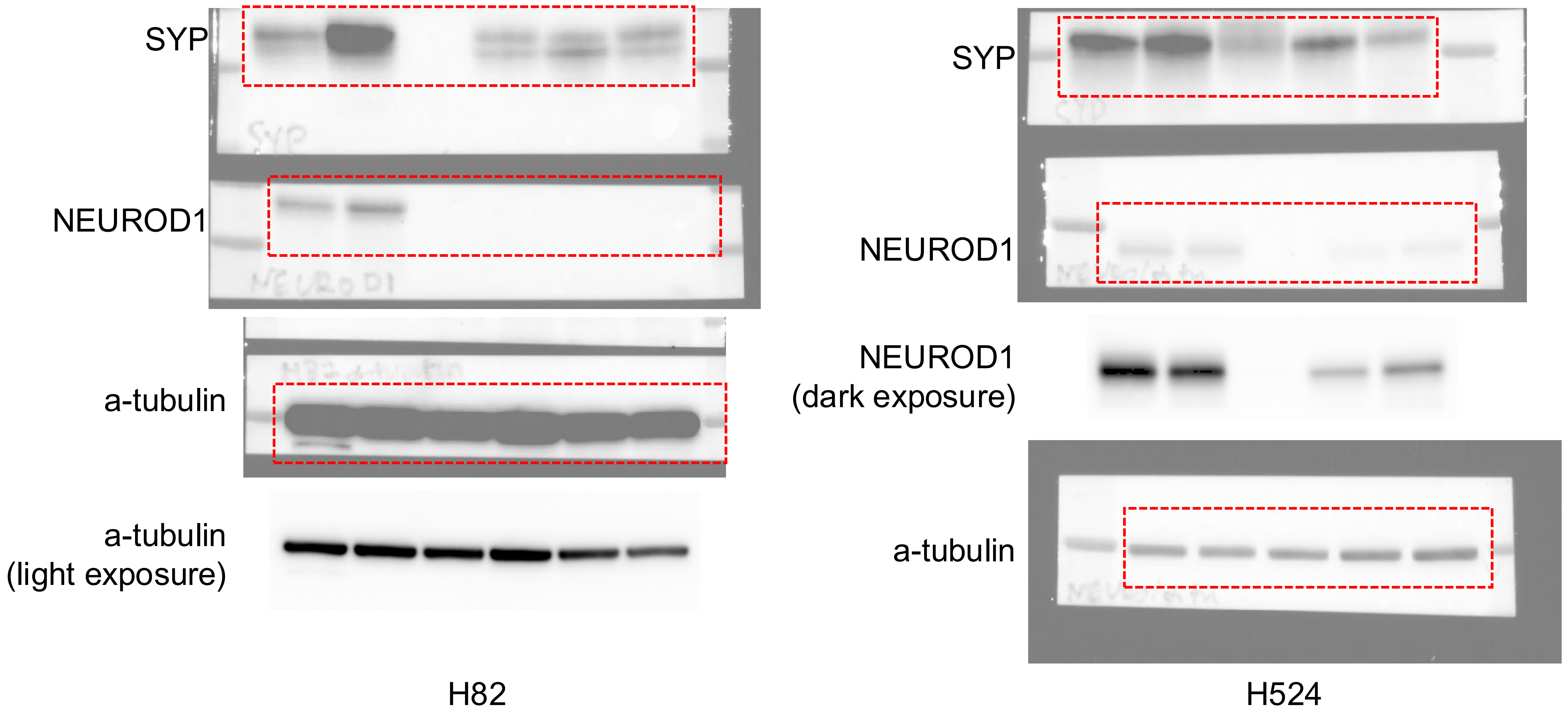

Full unedited image for Supplemental Figure 6B_2

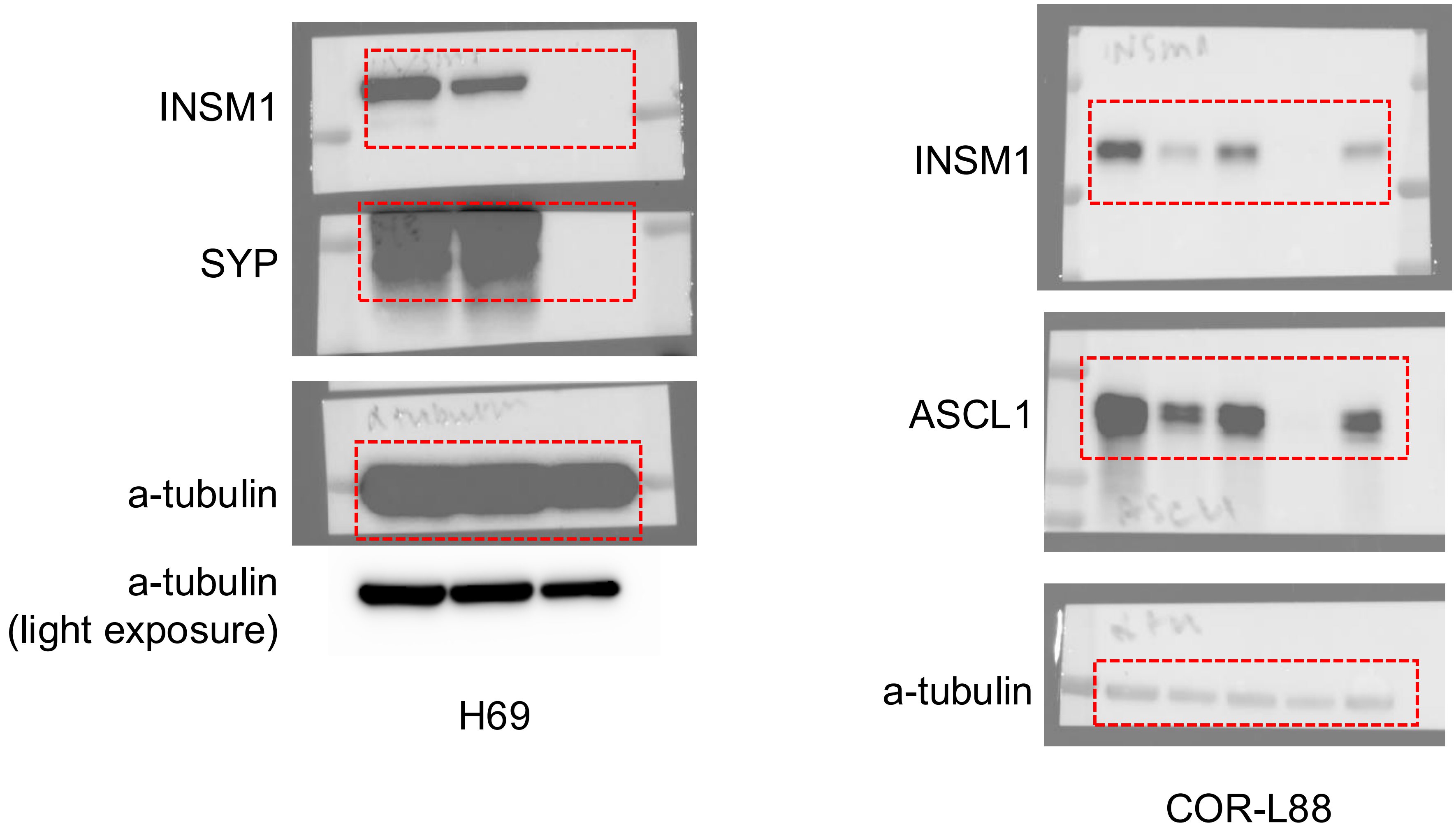

Full unedited image for Supplemental Figure 9A, E

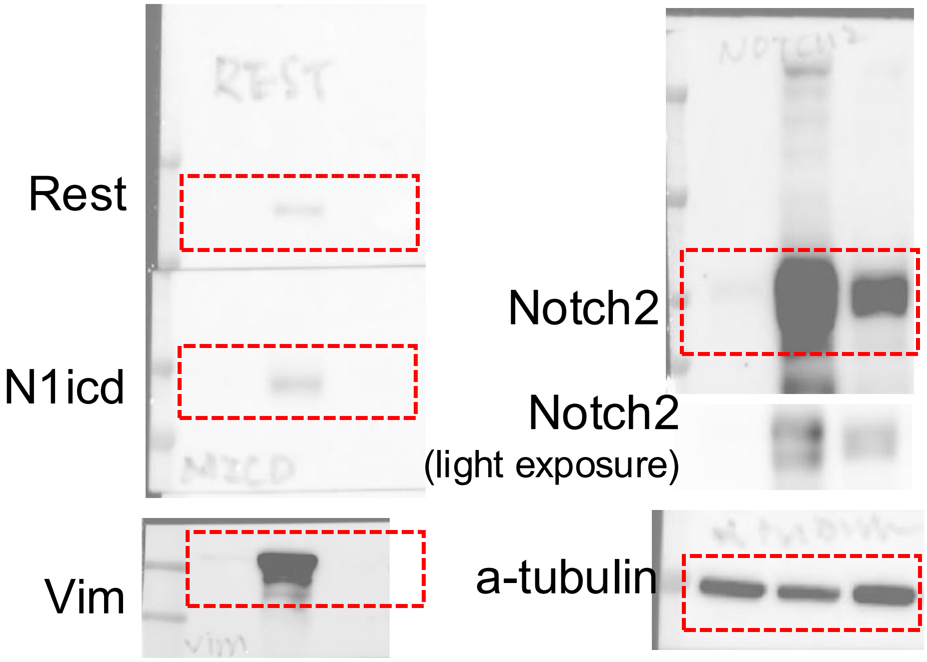

Figure S9A

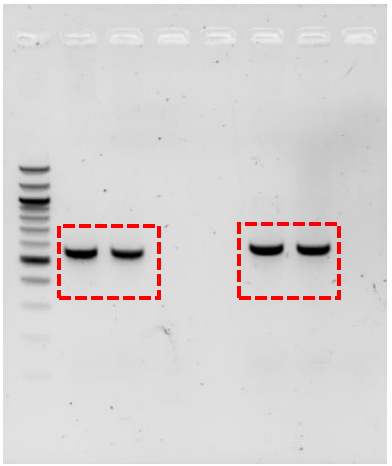

Recombined *Rb1* (left)

Recombined *Trp53* (right)

Figure S9E

Full unedited image for Supplemental Figure 9G, J

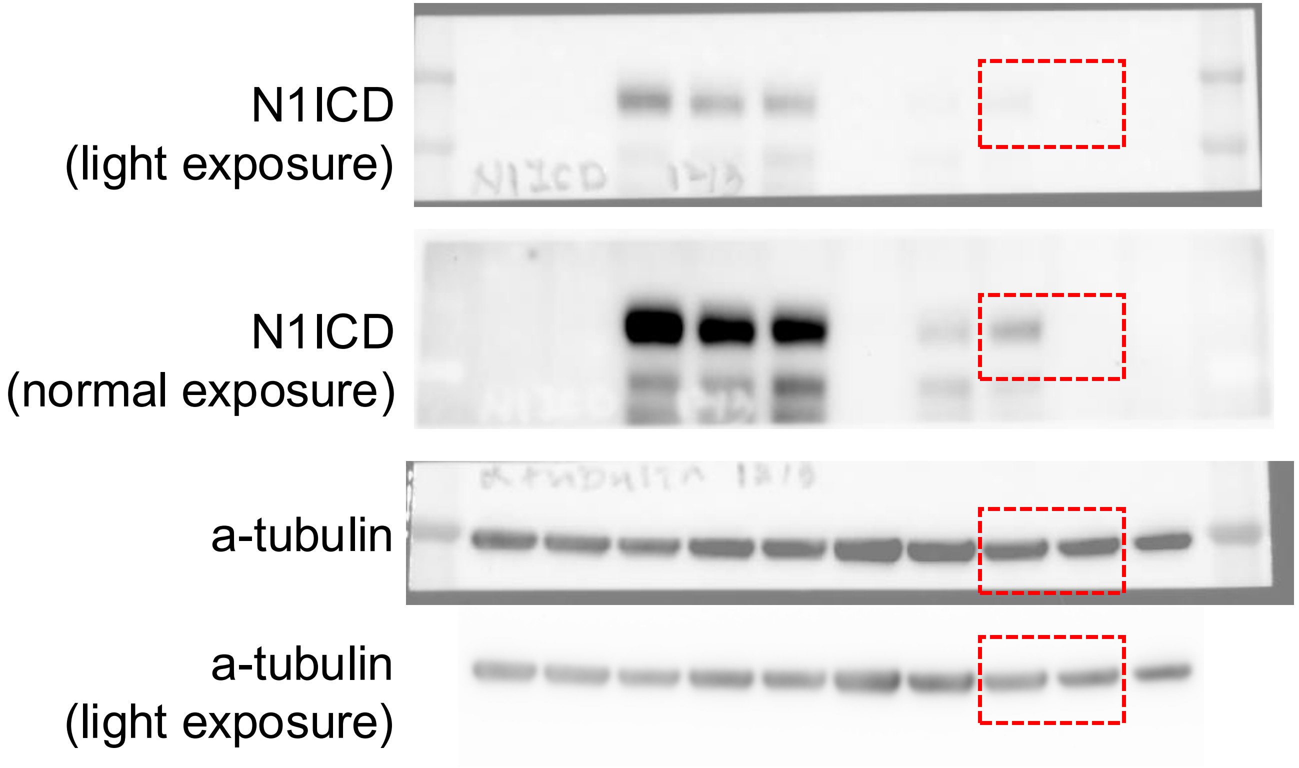

Figure S9G

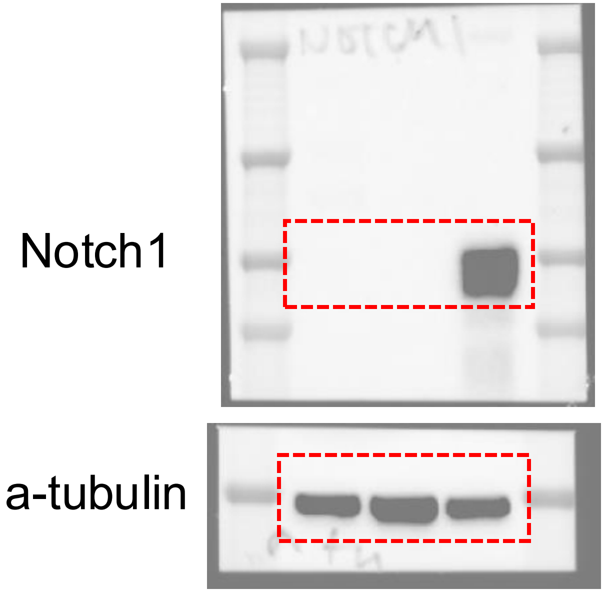

Figure S9J

Full unedited image for Supplemental Figure 10E, F

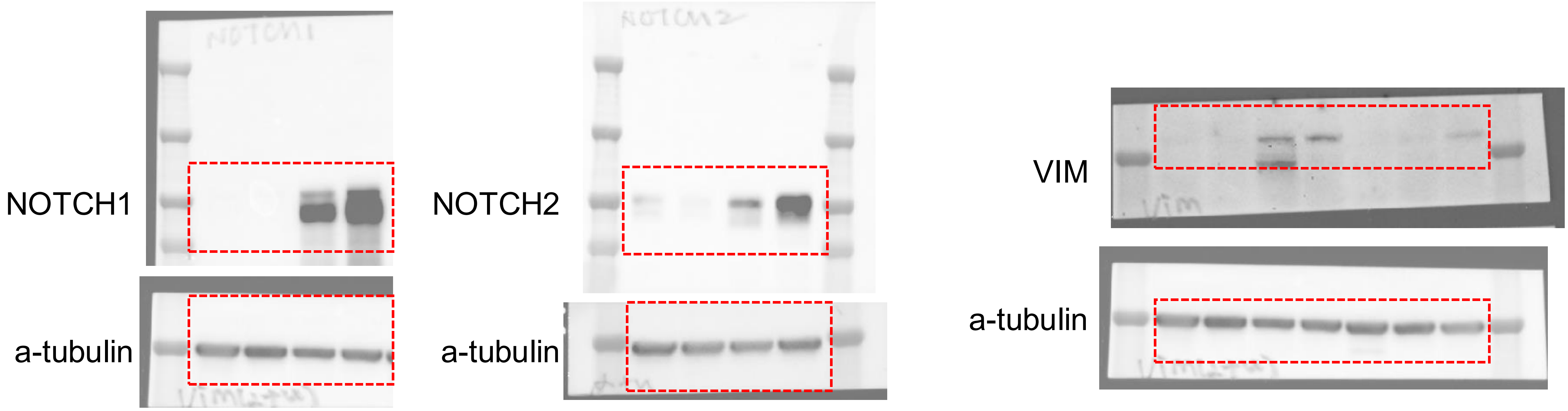

Figure S10E

Figure S10F

Full unedited image for Supplemental Figure 10H

STING

a-tubulin

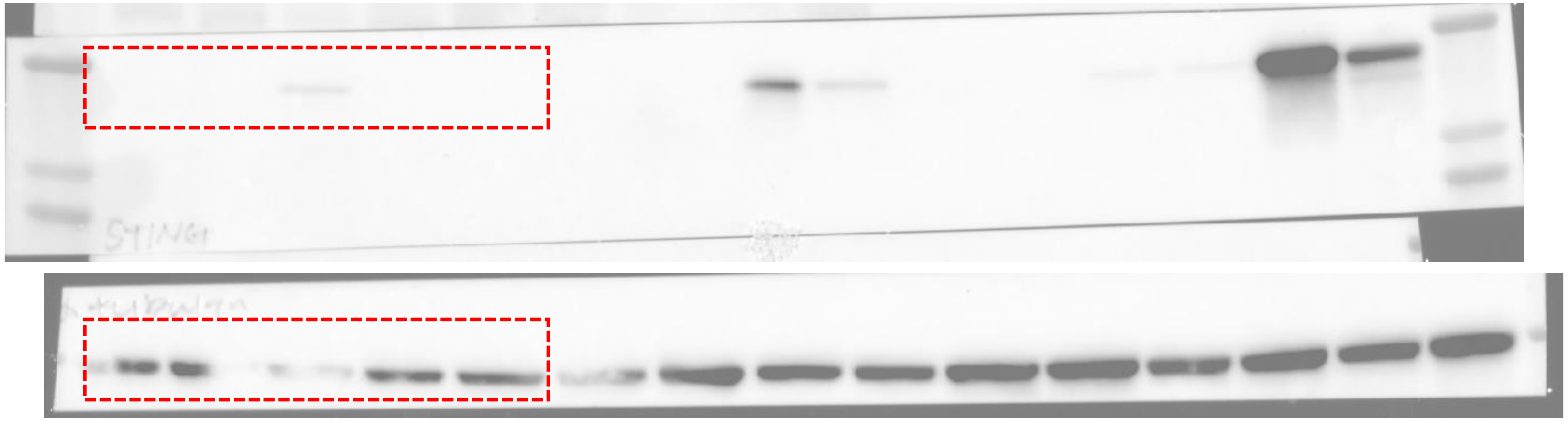

H82

STING

a-tubulin

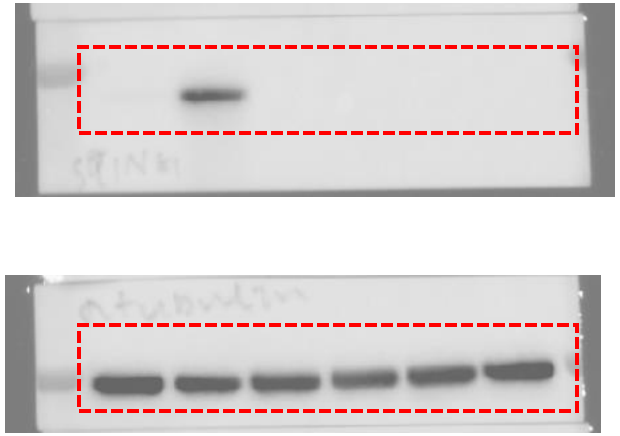

COR-L88

Full unedited image for Supplemental Figure 11B

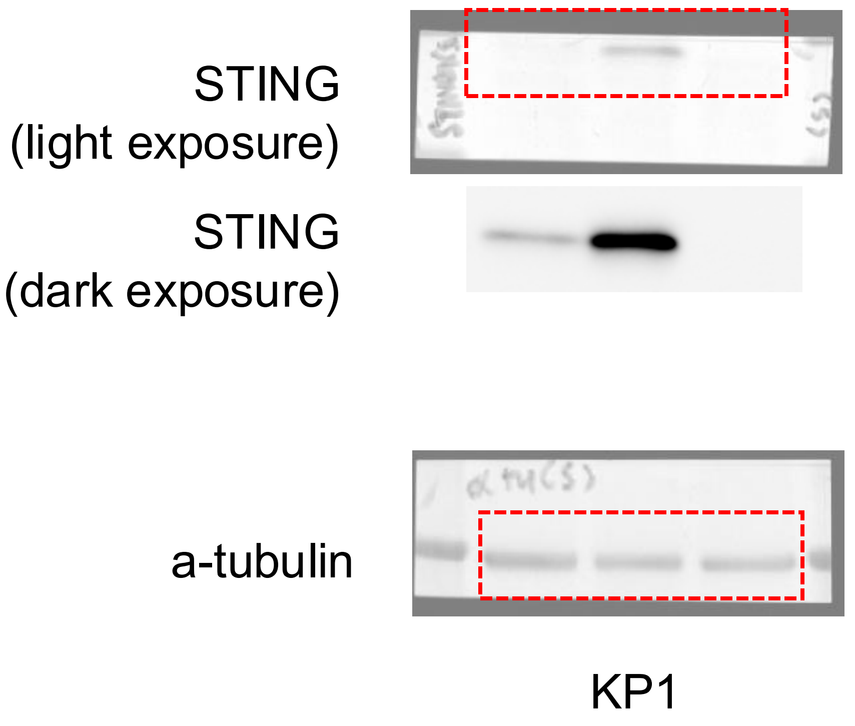

Full unedited image for Supplemental Figure 12

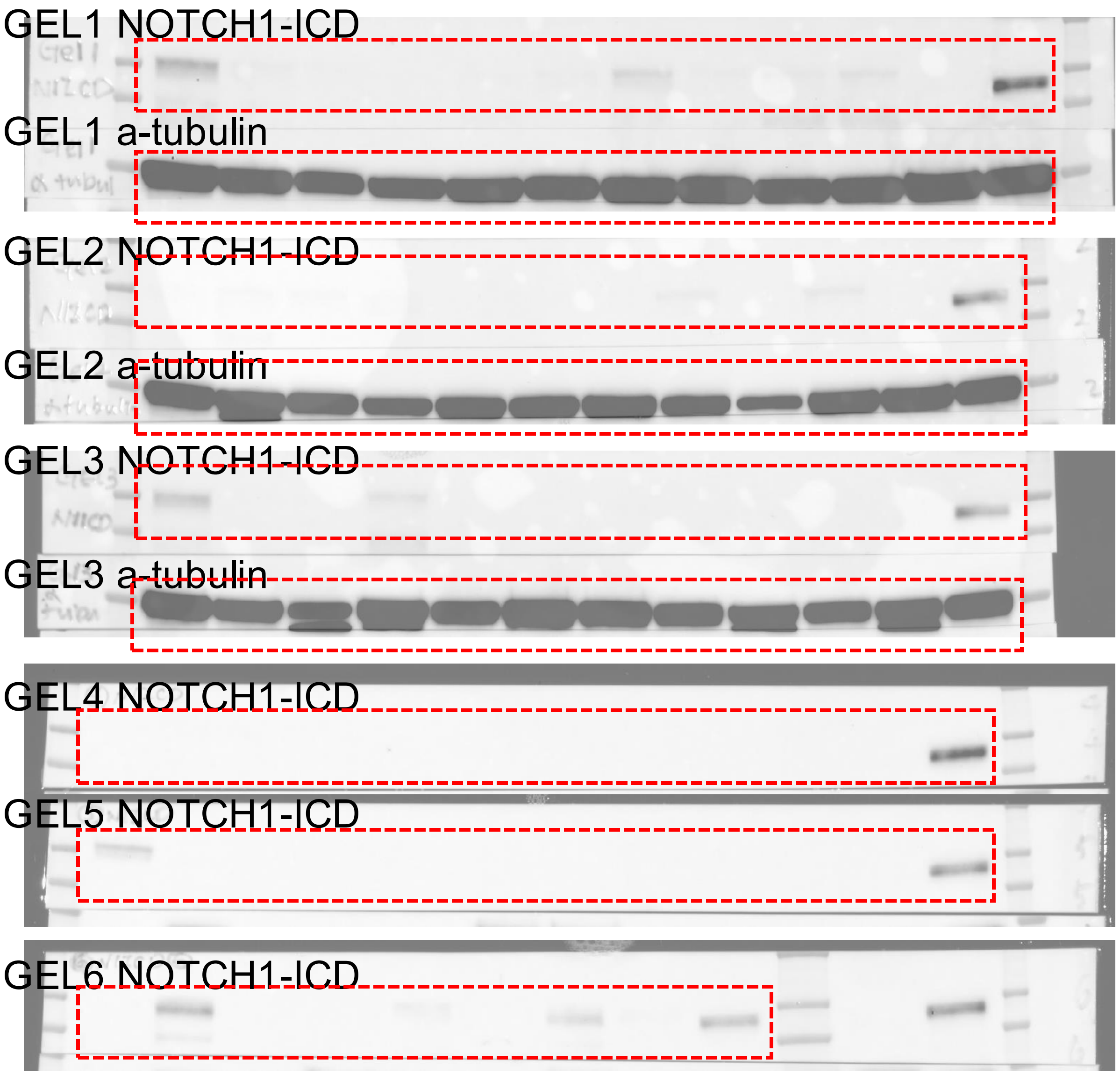

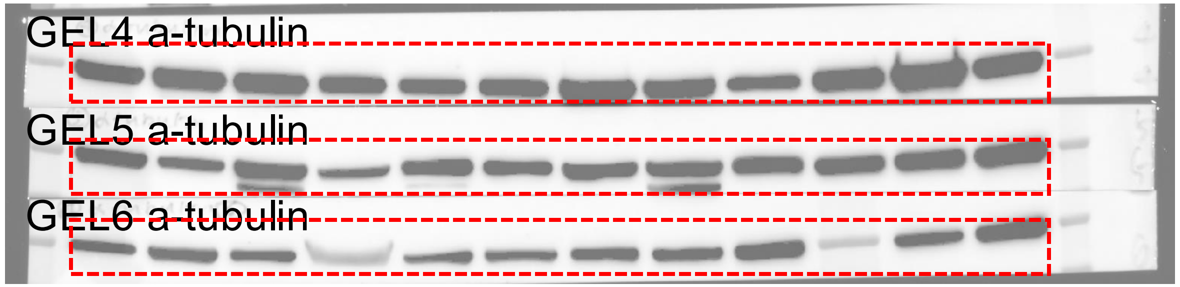

Supplemental Figure 12

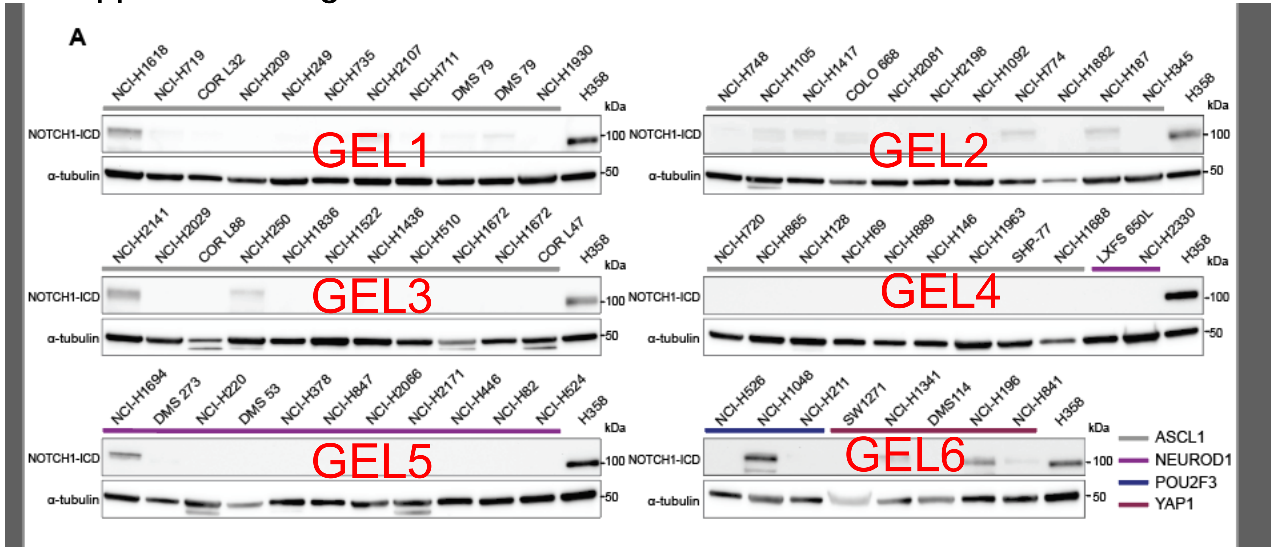

Supplement: Unedited blot and gel images [file jci-135-185423-s307.pdf]
